# Carbamothioate-mediated Selectfluor™ oxidation for the synthesis of benzenesulfonyl fluorides in chalcone derivatives†

**DOI:** 10.1039/d5ra03427b

**Published:** 2025-06-27

**Authors:** Boye Jiang, Juan Zhang, Boning Wang, Airui Zhou, Yan Zou, Qingguo Meng, Qingjie Zhao, Xiaoyun Chai, Conghao Gai

**Affiliations:** a Organic Chemistry Group, School of Pharmacy, Naval Medical University No. 325 Guo-he Road Shanghai 200433 P. R. China c.gai2@outlook.com; b School of Pharmacy, Yantai University Yantai P. R. China 264000

## Abstract

A carbamothioate-mediated method for synthesizing benzenesulfonyl fluorides *via* Selectfluor™ oxidation is reported, enabling compatibility with α,β-unsaturated chalcone derivatives without Michael addition side reactions. This approach offers moderate yields, broad functional group tolerance, and applications in covalent drug discovery, facilitating the incorporation of sulfonyl fluoride warheads into complex electrophilic scaffolds.

## Abstract 2

Novel carbamothioate-mediated sulfonyl fluoride synthesis enables chalcone functionalization without Michael addition side reactions.

## Graphical abstract

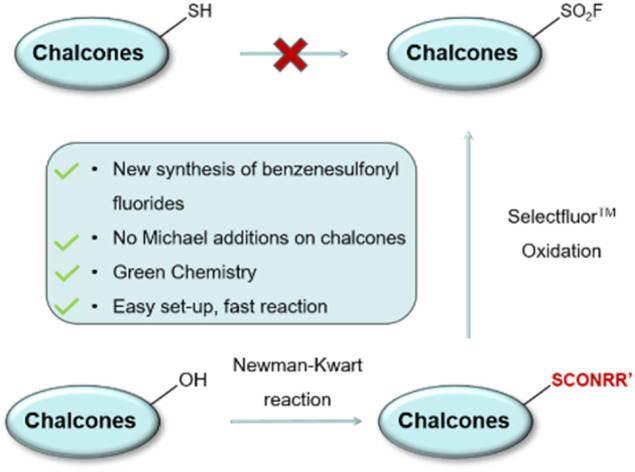

## Introduction

Sulfonyl fluorides can be prepared through various methods.^1–4^ Among the most effective approaches is the coupling of sulfonyl groups with fluorination agents, such as nucleophilic fluorination using reactive sulfonyl chlorides—though their high reactivity presents a key limitation.^4^ Alternatively, newer methods employ stable starting materials, including disulfides/thiols, sodium sulfonates, and aryl bromides, enabling the synthesis of sulfonyl fluorides under mild conditions with high yields.^5–8^ While traditional thiol oxidation remains a highly efficient route to benzenesulfonyl fluorides, the incorporation of this pharmacophore into anti-inflammatory chalcone derivatives has rarely been reported. This is due to the inherent challenge posed by the Michael acceptor motif in chalcones, which promotes inter- or intramolecular nucleophilic addition with thiol groups during oxidation. Herein, we firstly developed a method to introduce the –SO_2_F group into α,β-unsaturated carbonyl systems without side reactions. This breakthrough holds significant potential for covalent drug discovery and medicinal chemistry, paving the way for novel dual-covalent therapeutics.

## Results and discussion

Thiophenol exhibits nucleophilic behaviour, reacting readily with α,β-unsaturated carbonyl systems. We propose that this challenge can be addressed through a mild post-functionalisation strategy, which offers enhanced compatibility with sensitive electrophilic functionalities. By protecting thiophenol as a carbamate, its nucleophilicity is significantly attenuated, thereby suppressing undesired side reactions such as Michael addition. Accordingly, we investigated the carbamothioate-mediated synthesis of benzenesulfonyl fluorides.

**Scheme 1:**
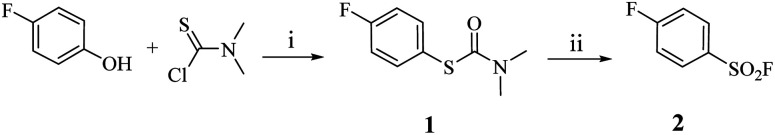
Reaction conditions: (i) NEt_3_, THF, r.t., 6 h; then diphenyl ether, 220 °C, microwave irradiation, 2 h, 86%; (ii) Selectfluor™, acetonitrile, H_2_O, Selectfluor™, 90 °C, 6 h, 40%; KF, acetonitrile, H_2_O, KF, 90 °C, 6 h, n/a; KHF_2_, cyanuric chloride, TMAC, acetonitrile, 60 °C, 12 h; then acetone, KHF_2_, r.t., 12 h, n/a.

The Newman–Kwart rearrangement was employed to convert the phenolic oxygen of 4-fluorophenol into the thiocarbamate intermediate 1. Subsequent fluorination was explored using various reported fluorinating agents;^9–12^ however, only Selectfluor™ afforded moderate conversion of 1 to the desired sulfonyl fluoride 2 (Scheme 1). Given the well-established fluorination and oxidative potency of Selectfluor™, we hypothesised its efficacy in promoting this transformation. Further investigation focused on optimising reaction time and Selectfluor™ loading (Table 1). The reaction time was systematically evaluated at 2, 4, 6, and 8 hours intervals, revealing that the highest yield of 2 was achieved within just 2 hours. Prolonging the reaction duration did not result in a statistically significant improvement in yield.

**Table 1:** Yield evaluation of compound 2 prepared with different amounts of Selectfluor™ and varying reaction times

Entry	Selectfluor™ amounts (eq.)	Reaction times (h)	Isolated yield (%)
1	2.5	2	22
2	6.5	2	40
3	10.5	2	41
4	4.5	2	40
5	4.5	4	40
6	4.5	6	37
7	4.5	8	42

Having established the optimal reaction time, we proceeded to evaluate the influence of Selectfluor™ stoichiometry on the reaction efficiency. The equivalents of Selectfluor™ were systematically varied (2.5, 4.5, 6.5, and 10.5 equiv.), revealing a clear dependence of both yield and purity on the oxidant loading. At 2.5 equiv., the reaction afforded a suboptimal yield alongside significant impurities, likely due to incomplete oxidation of intermediate 1.

Notably, the yield of 2 reached a maximum at 4.5 equiv. of Selectfluor™, suggesting that this stoichiometry provides sufficient oxidative capacity for efficient conversion. Intriguingly, further increasing the Selectfluor™ loading (up to 10.5 equiv.) did not improve the yield, implying that the reaction is not limited by oxidant availability beyond this threshold. This observation may indicate either a kinetic barrier or competing decomposition pathways at higher oxidant concentrations.

**Scheme 2:**
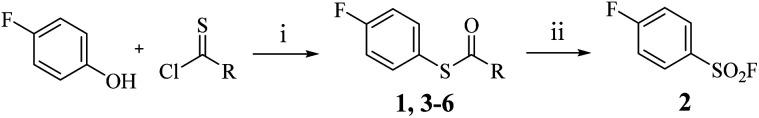
Reaction conditions: (i) NEt_3_, THF, r.t., 6 h; then diphenyl ether, 220 °C, microwave irradiation, 2 h; (ii) acetonitrile, H_2_O, Selectfluor™, 90 °C, 2 h.

The influence of the carbamate moiety on reaction selectivity was next investigated (Scheme 2 and Table 2). While the yields of thiocarbamate intermediates 1 and 3–6 showed no significant variation across five different amino-terminated thioacyl chlorides in the initial step with 4-fluorophenol, pronounced differences emerged during the oxidative fluorination to sulfonyl fluoride 2. Notably, the *N*-methyl-*N*-ethyl amino-terminated intermediate 3 resulted in a markedly diminished yield of 2 (40%, Table 2). This suggests that steric hindrance from the *N*-ethyl group may impede the oxidative fluorination step. In contrast, the morpholine-derived intermediate 6 maintained near-identical efficiency to the parent system (83%, Table 2), implying that the conformational constraint of the morpholine ring either minimally affects the transition state or may even facilitate the oxidation through favourable orbital alignment. These observations highlight the delicate balance between steric and electronic effects in determining the success of this transformation, where both the bulkiness and conformational flexibility of the carbamate moiety play critical roles (Table 2).

**Table 2:** Yield evaluation of compound 2 prepared with different thiocarbamate intermediates

Entry	R	Intermediates	Yield (%)	
			i	ii
1	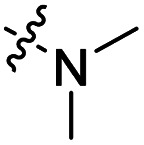	1	86	40
2	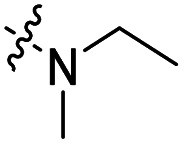	3	84	27
3	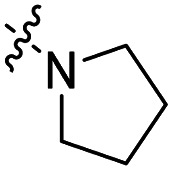	4	85	34
4	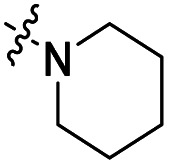	5	86	36
5	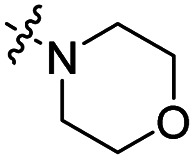	6	83	40

**Scheme 3:**
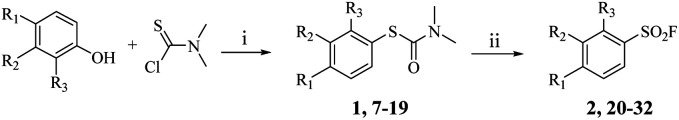
Reaction conditions: (i) NEt_3_, THF, r.t., 6 h; then diphenyl ether, 220 °C, microwave irradiation, 2 h; (ii) acetonitrile, H_2_O, Selectfluor™, 90 °C, 2 h.

The reaction demonstrated excellent functional group tolerance, accommodating both electron-donating and electron-withdrawing substituents (Scheme 3 and Table 3). As anticipated, the Newman–Kwart reaction efficiently converted a range of substituted phenols into the corresponding carbamate thioester intermediates in consistently high yields (Table 3, step i). Notably, the previously observed challenges in the sulfonyl fluorination step were substantially alleviated with certain substrates. In particular, the *para*-Cl-substituted derivative 25 and the dichloride analogue 28 afforded exceptional yields (∼90%), suggesting that inductive halogen effects may be at play. However, the strong electron-withdrawing groups such as cyano and nitro groups in compounds 16 and 17 significantly deactivated the oxidation fluorination process-possibly by reducing the reactivity of the thiocarbamate intermediate through conjugated effects.

**Table 3:** Study of method applicability

Entry	R_1_	R_2_	R_3_	Compd.	Yield (%)	Compd.	Yield (%)
1	–F	–H	–H	1	86	2	40
2	–CH_3_	–H	–H	7	85	20	68
3	–H	–CH_3_	–H	8	76	21	79
4	–H	–H	–CH_3_	9	76	22	64
5	–CH(CH_3_)_2_	–H	–H	10	88	23	74
6	–C(CH_3_)_3_	–H	–H	11	77	24	77
7	–Cl	–H	–H	12	79	25	90
8	–H	–Cl	–H	13	78	26	57
9	–H	–H	–Cl	14	86	27	68
10	–Cl	–H	–Cl	15	86	28	89
11	–CN	–H	–H	16	75	29	73
12	–NO_2_	–H	–H	17	81	30	79
13	–OCH_3_	–H	–H	18	80	31	72
14	–COOCH_3_	–H	–H	19	78	32	81

Nevertheless, the extension of this methodology to heteroaromatic systems proved problematic. Attempts to synthesise thiocarbamate intermediates from pyridine-based substrates were unsuccessful, precluding further exploration of the fluorination step. This limitation likely stems from the diminished nucleophilicity of the heteroaromatic hydroxyl group, compounded by potential coordination effects between the nitrogen lone pair and the thioacyl chloride reagent. Future studies might explore alternative activation strategies to overcome this constraint, particularly for medicinally relevant heterocyclic scaffolds.

**Fig. 1:**
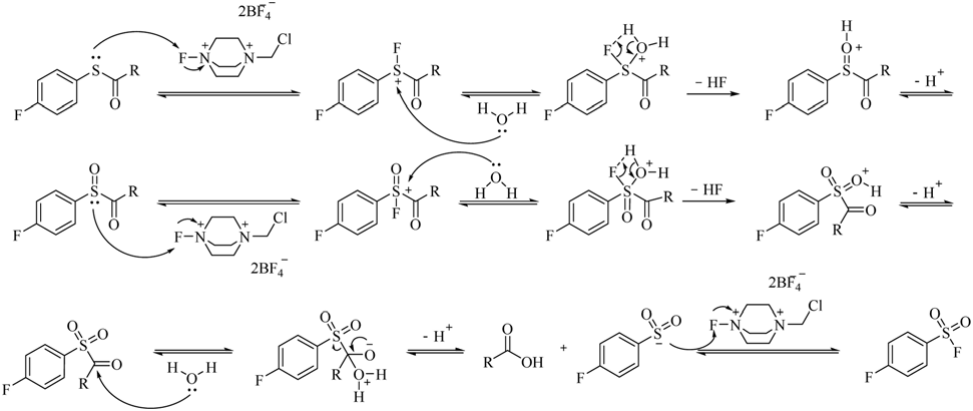
Reaction mechanism of Selectfluor™ oxidizing and fluorinating carbamothioates.

The proposed mechanism for the Selectfluor™-mediated oxidation of carbamothioates is outlined in Fig. 1, with supporting computational evidence provided in Fig. S1.† The transformation is initiated by nucleophilic attack of the carbamothioate sulphur lone pair on the electrophilic fluorine centre of Selectfluor™, forming a reactive S–F intermediate. This transient species is subsequently hydrolysed by water, which yields a sulfinylmethanamide intermediate. A second equivalent of Selectfluor™ then oxidises this intermediate to the sulfonyl stage, with the liberated HF rendering the reaction medium acidic—a feature that may facilitate subsequent steps by protonation of leaving groups.

Critical to the mechanism is the final oxidative fluorination, wherein the sulfonyl sulphur undergoes nucleophilic attack on a third equivalent of Selectfluor™, ultimately affording the sulfonyl fluoride product. Notably, the carbonyl carbon of the carbamate moiety is concomitantly attacked by water, leading to cleavage of the C–S bond *via* a tetrahedral intermediate and liberation of the sulfonyl group. This step-wise progression, which supported by DFT calculations (Fig. S1†), accounts for the observed stoichiometry (3 equiv. Selectfluor™ consumed per equivalent of product) and rationalises the acidic byproducts (2 equiv. HF) that accumulate during the reaction.

The computational data further corroborate the feasibility of this pathway, revealing low energy barriers for the key fluorination and hydrolysis steps. Intriguingly, the proposed mechanism bears resemblance to established sulfoxide-to-sulfonyl fluoride conversions, yet diverges in its initial activation through S–F bond formation rather than direct oxygen transfer. This distinction may explain the system's tolerance for diverse carbamate substituents, as the mechanism avoids high-energy intermediates that might otherwise be sensitive to steric or electronic effects.

The synthetic utility of this methodology was demonstrated through the preparation of covalent anti-inflammatory inhibitors C1–C6 from an in-house library. These compounds feature an α,β-unsaturated carbonyl scaffold, a structural motif that typically poses challenges in conventional thiophenol oxidation protocols due to propensity for Michael addition side reactions, often leading to insoluble polymeric byproducts.

**Scheme 4:**
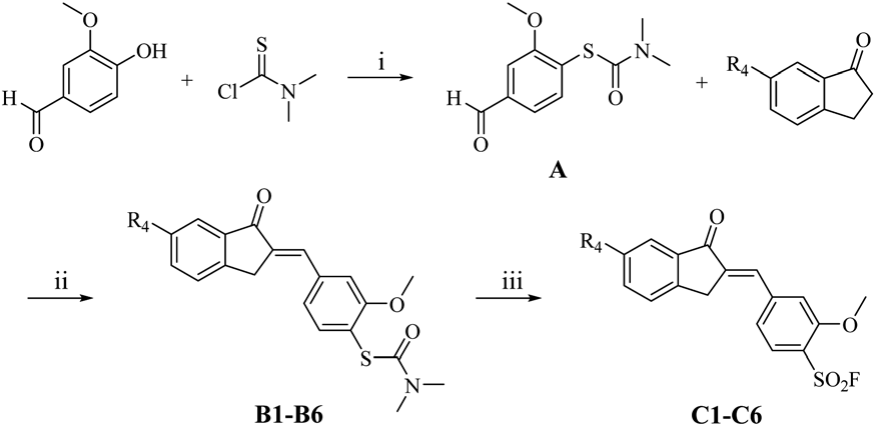
Reaction conditions: (i) NEt_3_, THF, r.t., 6 h; then diphenyl ether, 220 °C, microwave irradiation, 2 h; (ii) 20% NaOH (aq.), EtOH, r.t., 1 h; (iii) acetonitrile, H_2_O, Selectfluor™, 90 °C, 2 h.

Crucially, our carbamothioate-mediated approach proved exceptionally mild, enabling the synthesis of C1–C5 in good yields (Scheme 4 and Table 4) while preserving the integrity of the reactive enone system. This chemoselectivity is particularly noteworthy given the dual reactivity of the system: while the nucleophilic thiol group could potentially engage in intramolecular Michael addition with the α,β-unsaturated ketone, the carbamate protection effectively suppresses this pathway. Structural confirmation was obtained through X-ray crystallography of C1 (Fig. S2†), which unambiguously established the (*E*)-configuration of the double bond – a finding that underscores the method's ability to maintain delicate stereochemical integrity during sulfonyl fluoride formation. The successful application to these pharmaceutically relevant scaffolds highlights the potential of this methodology in targeted covalent inhibitor development, particularly for systems where traditional thiol oxidation strategies fail due to competing conjugate addition or polymerization.

**Table 4:** The synthesis pathway of C1–C5 and yields

Entry	R_4_	Compd.	Yield (%)	
			ii	iii
1	F	C1	90	60
2	CH_3_	C2	89	57
3	OCH_3_	C3	61	67
4	H	C4	98	60
5	OH	C5	71	21
6	NH_2_	C6	80	n/a

**Scheme 5:**
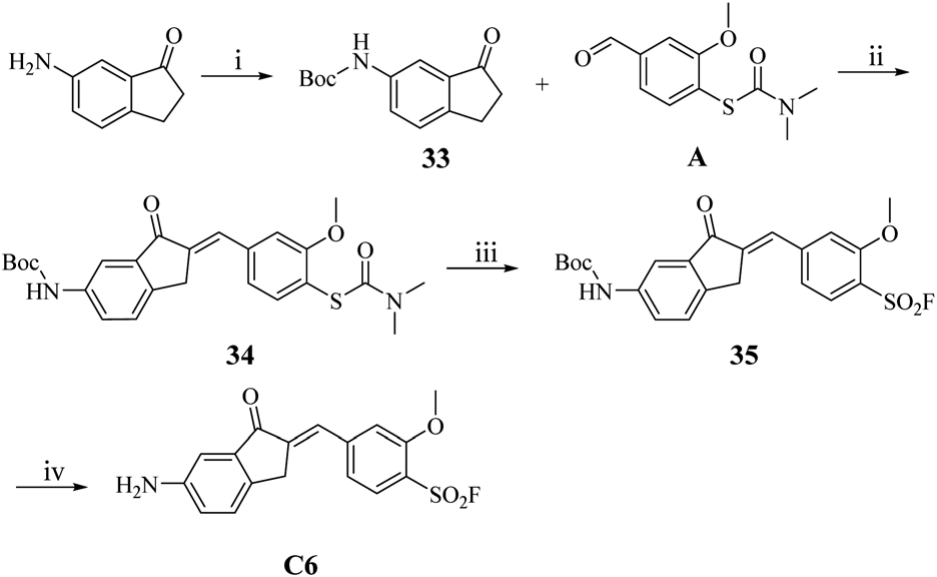
The synthesis route of compound C6. Reaction conditions: (i) DCM, (Boc)_2_O, r.t.; (ii) 20% NaOH (aq.), EtOH, r.t., 1 h; (iii) acetonitrile, H_2_O, Selectfluor™, 90 °C, 2 h; (iv) DCM, TFA, r.t.

However, the synthesis of C6 proved unsuccessful under standard conditions, likely due to the base-sensitive nature of the amino intermediate B6 under the acidic reaction environment. To circumvent this limitation, we implemented a protecting group strategy wherein the amino functionality was first protected as its Boc derivative using (Boc)_2_O prior to the aldol condensation step (Scheme 5). The resulting Boc-protected intermediate 34 underwent smooth sulfonyl fluoridation to afford the benzenesulfonyl fluoride derivative 35, which was subsequently deprotected to yield the target compound C6 in 86.8% yield.

## Conclusions

This study has developed a versatile carbamothioate-mediated synthesis of benzenesulfonyl fluorides *via* Selectfluor™ oxidation that demonstrates excellent compatibility with chalcone scaffolds. This method enables direct conversion of carbamate-protected thiophenols to sulfonyl fluorides while effectively suppressing undesired Michael additions to α,β-unsaturated carbonyl systems.

Of particular significance for medicinal chemists, this approach provides a robust platform for introducing sulfonyl fluoride warheads into complex drug-like molecules without compromising other electrophilic functionalities. The exceptional chemoselectivity, combined with operational simplicity and moderate yields, makes this methodology particularly valuable for developing novel covalent inhibitors. This advancement opens new possibilities for rational design of multi-warhead covalent drugs, addressing a growing need in targeted therapeutic development.

## Author contributions

C. Gai: conceptualization, methodology; B. Jiang, B. Wang, J. Zhang and A. Zhou: data curation; B. Jiang, J. Zhang and C. Gai: writing – original draft preparation; Y. Zou and Q. Meng: writing – reviewing and editing; Q. Zhao and X. Chai: supervision.

## Conflicts of interest

There are no conflicts to declare.

## Supplementary Material

RA-015-D5RA03427B-s001

RA-015-D5RA03427B-s002

## Data Availability

The data supporting this article, including the experimental procedures, characterization data, and copies of NMR spectra, have been included as part of the ESI.† Crystallographic data for C1 has been deposited at the CCDC under no. 2430014 and can be obtained from https://www.ccdc.cam.ac.uk.
