# Supplementary material for: Carbamothioate-mediated Selectfluor™ oxidation for the synthesis of benzenesulfonyl fluorides in chalcone derivatives
Source: RSC Adv. 2025 Jun 27;15(27):22018–22. doi: 10.1039/d5ra03427b (PMC12203147; doi:10.1039/d5ra03427b)
Supplement: RA-015-D5RA03427B-s001 [file RA-015-D5RA03427B-s001.pdf]

Supplementary Information (SI) for RSC Advances.
This journal is © The Royal Society of Chemistry 2025

Supplementary information

Content

General Information for the Synthesis 2
General method i 2
General method ii 2
General method iii 2
Analytical Equipment 13
NMR, HRMS, HPLC spectra 17

General Information for the Synthesis

General method i

In a 100 mL dry flask containing phenols (1 eq.), TEA (2 eq.) and THF were added, and the mixture was stirred at 0 °C under the argon atmosphere. Then, thionyl chloride (1.5 eq.) was added to the reaction mixture, which was vigorously stirred at room temperature for 6 h. The reaction mixture was diluted by H_2O and extracted with DCM. The organic layer was washed with brine, dried over anhydrous Na_2SO_4, and concentrated under vacuum to provide the crude product. The solution of crude product in diphenyl ether was transferred to a microwave reaction tube, sealed, and heated in a microwave reactor at 220 °C for 120 min. The reaction mixture was purified using silica gel chromatography to obtain corresponding intermediate dimethylcarbamothioates.

General method ii

To a solution of intermediate dimethylcarbamothioates (1 eq.) in acetonitrile were added an appropriate amount of water and Selectfluorm™ (4.5 eq.), and the mixture was stirred at 90 ℃ for 60 min. The solvent was diluted by DCM, then filtered and collected the filtrate. The filtrate dried over anhydrous Na_2SO_4, and concentrated under vacuum to provide the crude product, which was directly subjected to flash column chromatography eluted to give corresponding pure compound sulfonyl fluorides.

General method iii

To a stirred solution of intermediate dimethylcarbamothioates (1 eq.) and corresponding 1-indanones (1.5 eq.) in EtOH was slowly added 20% NaOH aqueous solution at room temperature for 60 min. The reaction mixture was diluted by appropriate H_2O and added 1 M/L HCl aqueous solution to adjust pH to 3-5. Then the mixture was extracted with DCM. The organic layer was washed with brine, dried over anhydrous Na_2SO_4, and concentrated under vacuum to provide the crude product. The crude product was purified by silica gel column chromatography to give corresponding intermediate *S*-(2-methoxy-4-((1-oxo-1, 3-dihydro-2*H*-inden-2-ylidene)methyl)phenyl) dimethylcarbamothioates.

Intermediate **B1-B6** and **34** were synthesized according to the procedure described for General method iii.

S-(4-((6-fluoro-1-oxo-1, 3-dihydro-2*H*-inden-2-ylidene)methyl)-2-methoxyphenyl) dimethylcarbamothioate (**B1)**

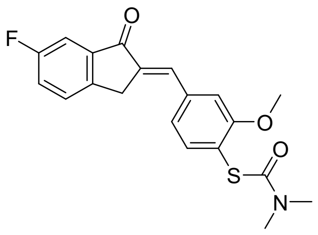

Yellow solid; yield 90%; m. p.: 109.6~111.4 °C; UV λ_{max}(CH_2Cl_2/nm) 330; IR ν_{max}/cm^{-1} 1623(C=C); ^{1}H NMR (300 MHz, $CDCl_3$) δ 7.66 (s, 1H), 7.58-7.55 (m, 1H), 7.54-7.51 (m, 1H), 7.37-7.29 (m, 3H), 7.17 (d, *J* = 0.99 Hz, 1H), 4.02 (s, 2H), 3.95 (s, 3H), 3.14 (s, 3H), 3.04 (s, 3H); ^{13}C NMR (101 MHz, DMSO-d_6) δ 193.06, 164.56, 163.56, 161.12, 160.23, 146.47, 139.35 (d, *J* = 7.4 Hz), 138.23, 136.95, 133.43, 129.20, 129.12, 123.08, 119.17, 114.52, 110.04, 109.82, 56.54, 36.99, 31.85; ^{19}F NMR (300 MHz, $CDCl_3$) δ -113.71 (s, 1F); LCMS (ESI) calcd. for $C_{20}H_{20}FNO_3S$ $[M+H]^+$: 372.1, found: 371.9.

S-(2-methoxy-4-((6-methyl-1-oxo-1, 3-dihydro-2*H*-inden-2-ylidene)methyl)phenyl) dimethylcarbamothioate (**B2)**

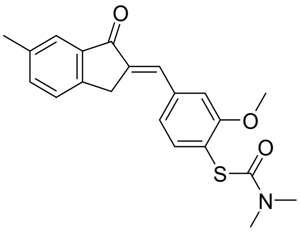

Yellow solid; yield 89%; m. p.: 190.1~191.4 °C; ^{1}H NMR (500 MHz, DMSO-d_6) δ 7.59 (d, *J* = 7.5 Hz, 1H), 7.57 (s, 1H), 7.56 (s, 1H), 7.49 (d, *J* = 7.5 Hz, 1H), 7.47-7.46 (m, 1H), 7.42-7.40 (m, 1H), 7.39-7.38 (m, 1H), 4.13 (s, 2H), 3.89 (s, 3H), 3.06 (s, 3H), 2.92 (s, 3H), 2.41 (s, 3H); ^{13}C NMR (101 MHz, DMSO-d_6) δ 193.77, 158.18, 149.28, 145.38, 141.44, 138.46, 138.21, 131.36, 129.79, 127.24, 123.50, 122.42, 119.87, 119.64, 116.20, 106.59, 57.64, 31.34; LCMS (ESI) calcd. for $C_{21}H_{22}NO_3S$ $[M+H]^+$: 368.1, found: 367.9.

S-(2-methoxy-4-((6-methoxy-1-oxo-1, 3-dihydro-2*H*-inden-2-ylidene)methyl)phenyl) dimethylcarbamothioate (**B3)**

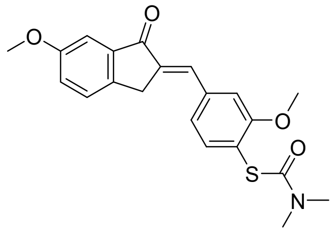

Yellow solid; yield 61%; m. p.: 186.1~187.4 °C; ^{1}H NMR (400 MHz, $CDCl_3$) δ 7.62 (s, 1H), 7.54 (d, *J* = 8.0 Hz, 1H), 7.43 (d, *J* = 8.4 Hz, 1H), 7.34 (d, *J* = 2.5 Hz, 1H), 7.32-7.29 (m, 1H), 7.20 (dd, *J* = 2.5, 8.4 Hz, 1H), 7.18 (s, 1H), 3.96 (s, 2H), 3.94 (s, 3H), 3.87 (s, 3H), 3.14 (s, 3H), 3.03 (s, 3H); ^{13}C NMR (101 MHz, $CDCl_3$) δ 194.13, 165.74, 160.07, 159.68, 142.38, 139.14, 138.45, 138.14, 136.62, 133.23, 126.98, 124.11, 122.68, 118.85, 113.65, 105.85, 56.22, 55.68, 37.06, 31.69; LCMS (ESI) calcd. for $C_{21}H_{22}NO_4S$ $[M+H]^+$: 384.1, found: 383.9.

S-(2-methoxy-4-((1-oxo-1, 3-dihydro-2*H*-inden-2-ylidene)methyl)phenyl) dimethyl-carbamothioate (**B4)**

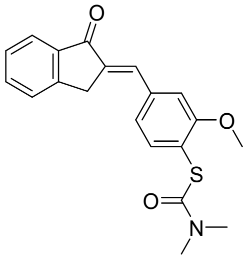

Yellow solid; yield 98%; m. p.: 163.1~164.2 °C; ^{1}H NMR (500 MHz, DMSO-d_6) δ 7.82 (d, *J* = 7.6 Hz, 1H), 7.76-7.73 (m, 1H), 7.71 (d, *J* = 7.6 Hz, 1H), 7.59 (t, *J* = 1.5 Hz, 1H), 7.52 (d, *J* = 8.0 Hz, 2H), 7.50-7.48 (m, 1H), 7.42 (dd, *J* = 8.0, 1.5 Hz, 1H), 4.21 (s, 2H), 3.90 (s, 3H), 3.08 (s, 3H), 2.92 (s, 3H); ^{13}C NMR (101 MHz, $CDCl_3$) δ 194.18, 165.74, 160.09, 149.54, 138.43, 138.15, 137.92, 135.79, 134.81, 133.36, 127.79, 126.25, 124.50, 122.65, 118.91, 113.70, 56.23, 37.06, 32.36; LCMS (ESI) calcd. for $C_{20}H_{20}NO_3S$ $[M+H]^+$: 354.1, found: 353.9.

S-(4-((6-hydroxy-1-oxo-1, 3-dihydro-2*H*-inden-2-ylidene)methyl)-2-methoxyphenyl) dimethylcarbamothioate (**B5)**

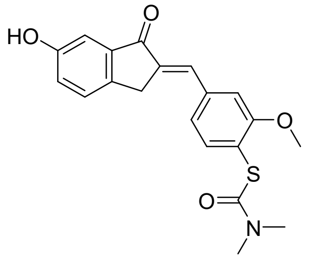

Yellow solid; yield 71%; m. p.: 277.0~278.4 °C; ^{1}H NMR (400 MHz, DMSO-d_6) δ 9.87 (s, 1H), 7.52 (s, 1H), 7.46 (m, 2H), 7.41 (s, 1H), 7.15 (dd, *J*=2.4, 8.2 Hz, 1H), 7.11 (d, *J*=2.4 Hz, 1H), 4.03 (s, 2H), 3.89 (s, 3H), 3.70 (s, 3H), 2.93 (s, 3H); ^{13}C NMR (101 MHz, DMSO-d_6) δ 193.70, 164.69, 160.21, 157.74, 141.18, 138.88, 138.39, 138.18, 137.59, 132.30, 127.79, 124.06, 122.91, 118.79, 114.23, 108.76, 56.46, 36.98, 31.58; LCMS (ESI) calcd. for $C_{20}H_{20}NO_4S$ $[M+H]^+$: 370.1, found: 369.9.

(*E*)-S-(4-((6-amino-1-oxo-1, 3-dihydro-2*H*-inden-2-ylidene)methyl)-2-methoxyphenyl) dimethylcarbamothioate (**B6**)

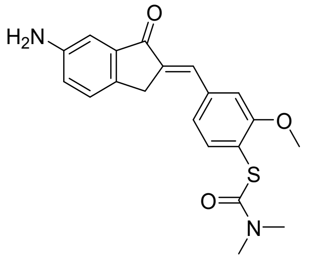

Yellow solid; yield 80%; m. p.: 201.3~202.6 °C; ^{1}H NMR (600 MHz, DMSO-d_6) δ 7.57 (d, *J* = 8.8 Hz, 1H), 7.54 (s, 1H), 7.49 (d, *J* = 7.9 Hz, 1H), 7.44 (s, 1H), 7.38 (d, *J* = 7.8 Hz, 1H), 7.35 (d, *J* = 6.3 Hz, 2H), 4.09 (s, 2H), 3.88 (s, 3H), 3.07 (s, 3H), 2.91 (s, 3H). ^{13}C NMR (151 MHz, DMSO-d_6) δ 193.53, 164.62, 160.25, 138.56, 138.33, 138.25, 137.30, 132.68, 127.93, 126.61, 123.05, 118.92, 114.38, 112.49, 56.54, 37.01, 31.82. LCMS (ESI) calcd. for $C_{20}H_{20}NO_4S$ $[M+H]^+$: 369.1, found: 368.9.

Tert-butyl (*E*)-(2-(4-((dimethylcarbamoyl)thio)-3-methoxybenzylidene)-3-oxo-2, 3-dihydro-1*H*-inden-5-yl)carbamate (**34**)

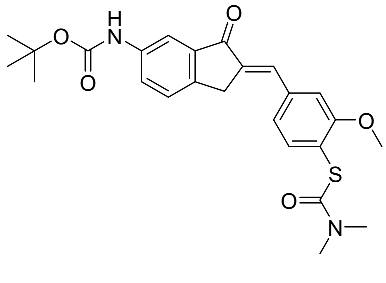

Yellow solid; yield 93%; m. p.: 243.9~244.1 °C; ^{1}H NMR (400 MHz, DMSO-d_6) δ 9.53 (s, 1H), 7.99 (s, 1H), 7.71 (d, J = 7.7 Hz, 1H), 7.54 (s, 1H), 7.52-7.47 (m, 2H), 7.39 (s, 1H), 7.34 (d, J = 7.7 Hz, 1H), 4.06 (s, 2H), 4.06 (s, 3H), 3.08 (s, 3H), 2.95 (s, 3H), 1.52 (s, 9H); ^{13}C NMR (101 MHz, DMSO-d_6) δ 193.61, 164.77, 160.19, 153.24, 143.85, 139.88, 138.32, 138.11, 137.09, 132.54, 126.90, 125.98, 122.91, 118.95, 114.06, 112.47, 79.63, 79.30, 78.97, 56.41, 36.99, 31.78, 28.61; LCMS (ESI) calcd. for $C_{25}H_{29}N_2O_5S$ [M+H]$^+$: 469.2, found: 468.8.

Compounds **C1-C5** and **35** were synthesized according to the procedure described for General method ii.

4-((6-fluoro-1-oxo-1, 3-dihydro-2*H*-inden-2-ylidene)methyl)-2-methoxybenzene-sulfonyl fluoride **(C1)**

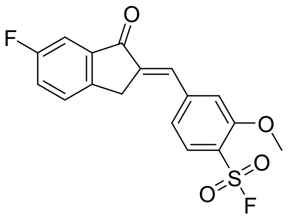

White solid; yield 60%; m. p.: 260.1~265.5 °C; UV λ_{max}(CH_2Cl_2/nm)310; IR ν_{max}/cm^{-1} 1414, 1206 (SO_2); ^{1}H NMR(300 MHz, $CDCl_3$) δ 7.94 (d, J = 8.3 Hz, 1H), 7.57 (s, 1H), 7.52-7.47 (m, 2H), 7.35-7.28 (m, 2H), 7.21 (s, 1H), 4.02 (s, 3H), 3.97 (s, 2H); ^{13}C NMR (101 MHz, DMSO-d_6) δ 192.92, 158.18, 146.65, 146.63, 144.85, 140.35, 138.98 (d, J = 7.5 Hz), 131.39, 129.27, 129.19, 123.46 (d, J = 24 Hz), 122.56, 116.51, 110.28, 110.06, 57.70, 31.73; ^{19}F NMR (300 MHz, $CDCl_3$) δ 59.21 (s, 1F), -112.90 (s, 1F); HRMS (ESI) calcd. for $C_{17}H_{13}F_2O_4S$ [M+H]$^+$: 351.0497, found: 351.0487.

2-methoxy-4-((6-methyl-1-oxo-1, 3-dihydro-2H-inden-2-ylidene)methyl)benzene-sulfonyl fluoride **(C2)**

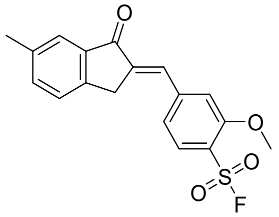

White solid; yield 57%; m. p.: 261.6~262.7°C; ^{1}H NMR (400 MHz, DMSO-d_6) δ 8.01 (d, J = 8.3 Hz, 1H), 7.79 (s, 1H), 7.64 (s, 1H), 7.62-7.60 (m, 2H), 7.59 (s, 1H), 5.76 (s, 1H), 4.18 (s, 2H), 4.10 (s, 3H), 2.41 (s, 3H); ^{13}C NMR (101 MHz, DMSO-d_6) δ 193.78, 160.26, 147.97, 138.38, 138.26, 137.81, 137.19, 136.67, 132.56, 126.91, 124.10, 123.14 (d, J = 20 Hz), 114.37, 56.55, 55.40, 37.01, 31.97, 21.19; ^{19}F NMR (400 MHz, DMSO-d_6) δ 59.94 (s, 1F); HRMS (ESI) calcd. for $C_{18}H_{16}FO_4S$ $[M+H]^+$: 347.0748, found: 347.0748.

2-methoxy-4-((6-methoxy-1-oxo-1, 3-dihydro-2*H*-inden-2-ylidene)methyl)benzene-sulfonyl fluoride (**C3)**

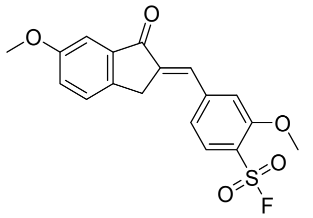

White solid; yield 67%; m. p.: 227.6~228.0°C; ^{1}H NMR (400 MHz, DMSO-d_6) δ 8.00 (d, J =8.4 Hz, 1H), 7.78 (s, 1H), 7.63-7.59 (m, 3H), 7.33 (dd, J =2.2, 8.4 Hz, 1H), 7.28 (d, J =2.2 Hz, 1H), 4.14 (s, 2H), 4.09 (s, 3H), 3.85 (s, 3H); ^{13}C NMR (101 MHz, DMSO-d_6) δ 193.44, 159.79, 158.18, 145.10, 143.39, 140.85, 138.52, 131.39, 130.68, 128.10, 124.65, 122.52, 116.39, 106.26, 57.68, 56.07, 31.49; ^{19}F NMR (400 MHz, DMSO-d_6) δ 59.95 (s, 1F); HRMS (ESI) calcd. for $C_{18}H_{16}FO_5S$ $[M+H]^+$: 363.0697, found: 363.0697.

2-methoxy-4-((1-oxo-1, 3-dihydro-2*H*-inden-2-ylidene)methyl)benzenesulfonyl fluoride (**C4)**

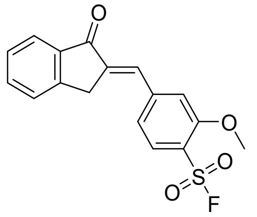

Yellow solid; yield 60%; m. p.: 232.3~233.5°C; ^{1}H NMR (500 MHz, DMSO-d_6) δ 8.02 (d, J = 8.0 Hz, 1H), 7.84 (d, J = 8.0 Hz, 1H), 7.80 (s, 1H), 7.78-7.75 (m, 1H), 7.71 (d, J = 7.6 Hz, 1H), 7.65-7.63 (m, 2H), 7.52 (t, J = 7.6 Hz, 1H), 4.25 (s, 2H), 4.11 (s, 3H); ^{13}C NMR (101 MHz, DMSO-d_6) δ 193.65, 158.19, 150.73, 145.08, 140.14,

137.25, 136.03, 131.40, 130.76, 128.43, 127.25, 124.53(d, J = 24 Hz), 122.54, 116.41, 68.06, 57.70, 32.20; ^{19}F NMR (400 MHz, DMSO-d_6) δ 59.94 (s, 1F); HRMS (ESI) calcd. for $C_{17}H_{14}FO_4S$ $[M+H]^+$: 333.0591, found: 333.0583.

4-((6-hydroxy-1-oxo-1, 3-dihydro-2*H*-inden-2-ylidene)methyl)-2-methoxybenzene-sulfonyl fluoride (**C5)**

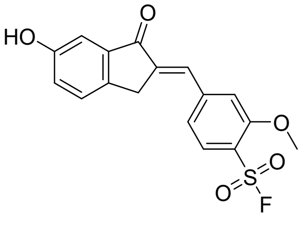

Yellow solid; yield 21%; m. p.: 246.8~247.1 °C; ^{1}H NMR (400 MHz, DMSO-d_6) δ 9.92 (s, 1H), 7.98 (d, J =8.3 Hz, 1H), 7.75-7.73 (m, 1H), 7.60-7.57 (m, 1H), 7.48 (d, J =8.3 Hz, 1H), 7.26 (d, J =8.2 Hz, 1H), 7.17 (dd, J =2.2, 8.2 Hz, 1H), 7.10 (d, J =2.2 Hz, 1H), 4.09 (s, 3H), 4.07 (s, 2H); ^{13}C NMR (101 MHz, DMSO-d_6) δ 193.50, 158.14, 157.80, 145.12, 144.87, 141.44, 141.00, 138.49, 131.34, 130.51, 127.96, 124.57, 122.42, 116.36, 108.79, 57.62, 31.42; ^{19}F NMR (400 MHz, DMSO-d_6) δ 59.95 (s, 1F); HRMS (ESI) calcd. for $C_{17}H_{14}FO_5S$ $[M+H]^+$: 349.0540, found: 349.0527.

Tert-butyl (*E*)-(2-(4-(fluorosulfonyl)-3-methoxybenzylidene)-3-oxo-2, 3-dihydro-1*H*-inden-5-yl)carbamate (**35)**

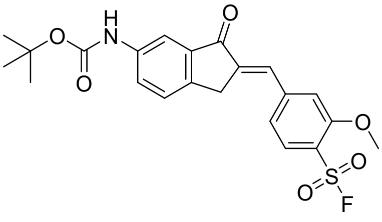

White solid; yield 14.1%; m. p.: 231.2~232.2 °C; ^{1}H NMR (400 MHz, DMSO-d_6) δ 9.66 (s, 1H), 8.05-7.96 (m, 2H), 7.78-7.70 (m, 2H), 7.63-7.54 (m, 2H), 4.10 (s, 3H), 3.65 (s, 1H), 1.50 (s, 9H), 1.23 (s, 2H); ^{13}C NMR (101 MHz, DMSO-d_6) δ 193.59, 158.20, 153.31, 145.14, 144.35, 140.71, 139.99, 137.74, 131.39, 130.63, 127.32, 126.47, 122.57, 116.31, 112.39, 57.68, 31.61, 29.49, 28.59; ^{19}F NMR (400 MHz, DMSO-d_6) δ 59.93 (s, 1F); LCMS (ESI) calcd. for $C_{22}H_{23}FNO_6S$ $[M+H]^+$: 448.1, found: 447.8.

Compound tert-butyl (3-oxo-2, 3-dihydro-1*H*-inden-5-yl)carbamate (**33**)

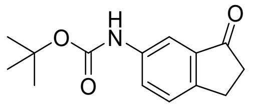

200 mg (1.36 mmol) 6-amino-2,3-dihydro-1*H*-inden-1-one were dissolved in 5 mL dichloromethane, stirred for 10 min in ice bath, then 327 mg (1.49 mmol) of di-tert-butyl dicarbonate are added and stirred for a further 16 h at RT. The mixture was added to water, extracted with dichloromethane, the combined organic phases were washed with sat. sodium chloride solution and the solvents removed on a rotary evaporator. The crude product was purified by silica gel column chromatography. Yield 208 mg (61.9% of theory) as a yellow solid. ^{1}H NMR (400 MHz, $CDCl_3$) δ 7.77 (d, *J* = 8.0 Hz, 1H), 7.61 (s, 1H), 7.40 (d, *J* = 8.0 Hz, 1H), 6.75 (s, 1H), 3.10-3.06 (m, 2H), 2.71-2.68 (m, 2H), 1.52 (s, 9H). ^{13}C NMR (101 MHz, $CDCl_3$) δ 206.73, 152.78, 149.67, 138.04, 127.03, 125.84, 112.84, 77.07, 36.76, 28.32, 25.27. LCMS (ESI) calcd. for $C_{14}H_{18}NO_3$ $[M+H]^+$: 248.1, found: 247.8.

Compound (*E*)-4-((6-amino-1-oxo-1, 3-dihydro-2*H*-inden-2-ylidene)methyl)-2-methoxybenzenesulfonyl fluoride (**C6**)

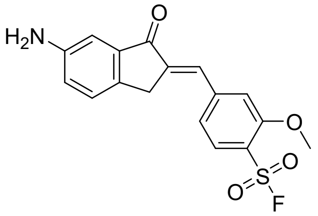

A suspension of **35** (46 mg, 0.11 mmol) in DCM (6 ml) was cooled down to 0°C before adding slowly TFA (30 mg, 0.76 mmol) and stirred at RT for 1 h. The reaction mixture was diluted with DCM. An aqueous solution of 1M/L NaOH was added, followed by an extraction with DCM. Then the combined organic layer was dried over Na_2SO_4, filtered and concentrated at reduced pressure. The crude product was purified by silica gel column chromatography. Yield 33 mg (86.8% of theory) as a yellow solid. m. p.: 241.7~242.3 °C; ^{1}H NMR (400 MHz, DMSO-d_6) δ 7.98 (d, *J* = 8.3 Hz, 1H), 7.74 (s, 1H), 7.57 (d, *J* = 8.3 Hz, 1H), 7.52 (s, 1H), 7.33 (d, *J* = 8.2 Hz, 1H), 6.99 (dd, *J* = 2.1, 8.2 Hz, 1H), 6.92 (d, *J* = 1.9 Hz, 1H), 5.44 (s, 2H), 4.09 (s, 3H), 4.01 (s, 2H); ^{13}C NMR (101 MHz, DMSO-d_6) δ 193.78, 158.18, 149.28, 145.38, 141.44, 138.46, 138.21, 131.36, 129.79, 127.24, 123.50, 122.42, 119.75 (d, *J* = 23 Hz), 116.20, 106.58, 57.63,

31.34. ^{19}F NMR (400 MHz, DMSO-d_6) δ 59.97 (s, 1F); HRMS (ESI) calcd. for $C_{17}H_{15}FNO_4S$ [M+H]$^+$ 348.0700, found: 348.0722.

Intermediate **A** and compounds **3~19** were synthesized according to the procedure described for General method i.

S-(4-formyl-2-methoxyphenyl) dimethylcarbamothioate (**A**)

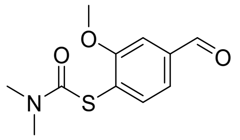

White solid; yield 73%; m. p.: 187.1~194.4 °C; UV λ_{max}(CH_2Cl_2/nm) 233; IR v_{max}/cm^{-1} 1665(C=O); ^1H NMR (300 MHz, $CDCl_3$;) δ 9.99 (s, 1H), 7.69 (d, 1H, *J*= 7.6 Hz), 7.48-7.45 (m, 2H), 3.94 (s, 3H), 3.14 (s, 3H), 3.03 (s, 3H); LCMS (ESI) calcd. for $C_{11}H_{14}NO_3S$ [M+H]$^+$: 240.1, found: 239.90.This compound was known.[1]

S-(4-fluorophenyl) dimethylcarbamothioate (**1**)

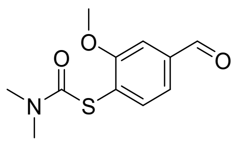

Colorless oil; yield 86%. UV λ_{max}(CH_2Cl_2/nm) 242; IR v_{max}/cm^{-1} 1671(C=O); ^1H NMR (300 MHz, $CDCl_3$) δ 7.49-7.42 (m, 2H), 7.11-7.03 (m, 2H), 3.05 (s, 6H); ^{19}F NMR (300 MHz, $CDCl_3$) δ -111.83 (s, 1F); LCMS (ESI) calcd. for $C_9H_{11}FNOS$ [M+H]$^+$: 200.1, found: 200.0. This compound was known.[2]

S-(4-fluorophenyl) ethyl(methyl)carbamothioate (**3**)

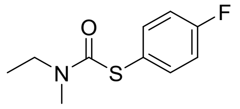

Colorless oil; yield 84%. UV λ_{max}(CH_2Cl_2/nm) 242; IR v_{max}/cm^{-1} 1667(C=O); ^1H NMR (300 MHz, $CDCl_3$) δ 7.48-7.44 (m, 2H), 7.10-7.04 (m, 2H), 3.44 (q, *J*= 7.1 Hz, 2H), 3.01 (s, 3H), 1.26-1.15 (m, 3H); ^{13}C NMR (101 MHz, DMSO-d_6) δ 164.41, 161.96, 138.38 (d, *J* = 8.7 Hz), 124.82, 116.58, 116.36, 44.47, 34.44, 13.30, 12.78. ^{19}F NMR (300 MHz, $CDCl_3$) δ -111.94 (s, 1F); LCMS (ESI) calcd. for $C_{10}H_{13}FNOS$ [M+H]$^+$: 214.1, found: 214.0.

S-(4-fluorophenyl) pyrrolidine-1-carbothioate (**4**)

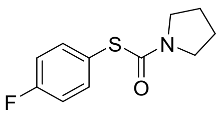

White solid; yield 85%; m. p.: 66.5~66.7 °C; UV λ_{max}(CH_2Cl_2/nm) 243; IR ν_{max}/cm^{-1} 1652(C=O); ^1H NMR (300 MHz, $CDCl_3$) δ7.51-7.44 (m, 2H), 7.11-7.02 (m, 2H), 3.53-3.45 (m, 4H), 2.00-1.89 (m, 4H).^{13}C NMR (101 MHz, DMSO-d_6) δ 164.36, 162.64, 161.91, 138.07 (d, J = 8.7 Hz), 124.78, 124.75, 116.61, 116.39, 47.80, 46.22, 25.67, 24.53. ^{19}F NMR (300 MHz, $CDCl_3$) δ -112.05 (s, 1F); LCMS (ESI) calcd. for $C_{11}H_{13}FNOS$ $[M+H]^+$: 226.1, found: 225.9.

S-(4-fluorophenyl) piperidine-1-carbothioate (**5**)

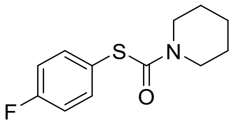

White solid; yield 86%; m. p.: 74.3~76.9 °C; UV λ_{max}(CH_2Cl_2/nm) 244; IR ν_{max}/cm^{-1} 1656(C=O); ^1H NMR (300 MHz, $CDCl_3$) δ 7.49-7.44 (m, 2H), 7.10-7.04 (m, 2H), 3.55-3.51 (m, 4H), 1.70-1.60 (m, 6H); ^{19}F NMR (300 MHz, $CDCl_3$) δ -111.97 (s, 1F); LCMS (ESI) calcd. for $C_{12}H_{15}FNOS$ $[M+H]^+$: 240.1, found: 239.9. This compound was known.[3]

S-(4-fluorophenyl) morpholine-4-carbothioate (**6**)

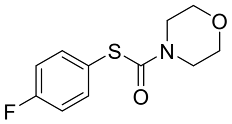

White solid; yield 83%; m. p.: 79.1~73.3 °C; UV λ_{max}(CH_2Cl_2/nm) 241; IR ν_{max}/cm^{-1} 1664(C=O); ^1H NMR (300 MHz, $CDCl_3$) δ 7.50-7.43 (m, 2H), 7.13-7.05 (m, 2H), 3.75-3.71 (m, 4H), 3.61-3.58 (m, 4H); ^{19}F NMR (300 MHz, $CDCl_3$) δ -111.32 (s, 1F); LCMS (ESI) calcd. for $C_{11}H_{13}FNO_2S$ $[M+H]^+$: 242.1, found: 241.90. This compound was known.[3]

S-(*p*-tolyl) dimethylcarbamothioate (**7**)

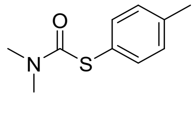

White solid; yield 85%; m. p.: 36.0~38.2°C; UV λ_{max}(CH_2Cl_2/nm) 244; IR ν_{max}/cm^{-1} 1670(C=O); ^1H NMR (300 MHz, $CDCl_3$) δ 7.39-7.35 (m, 2H), 7.19 (d, J= 7.9

Hz, 2H), 3.05 (s, 6H), 2.36 (s, 3H); LCMS (ESI) calcd. for $C_{10}H_{14}NOS$ $[M+H]^+$: 196.1, found:196.0. This compound was known.[2]

S-(*m*-tolyl) dimethylcarbamothioate (**8**)

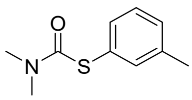

Colorless oil; yield 76%; UV λ_{max}(CH_2Cl_2/nm) 245; IR ν_{max}/cm^{-1} 1670(C=O); ^{1}H NMR (300 MHz, $CDCl_3$) δ 7.33-7.32 (m, 1H), 7.30-7.26 (m, 2H), 7.20-7.18 (m, 1H), 3.05-3.04 (m, 6H), 2.35 (s, 3H).; ^{13}C NMR (101 MHz, DMSO-*d*$_6$) δ 165.53, 138.74, 136.37, 132.96, 130.24, 129.20, 128.76, 36.94, 21.21. LCMS (ESI) calcd. for $C_{10}H_{14}NOS$ $[M+H]^+$: 196.1, found:196.0.

S-(*o*-tolyl) dimethylcarbamothioate (**9**)

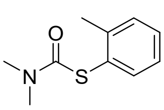

Colorless oil; yield 76%; UV λ_{max}(CH_2Cl_2/nm) 242; IR ν_{max}/cm^{-1} 1669(C=O); ^{1}H NMR (300 MHz, $CDCl_3$) δ 7.47 (d, *J*= 7.4 Hz, 1H), 7.34-7.29 (m, 2H), 7.22-7.16 (m, 1H), 3.07 (s, 6H), 2.41 (s, 3H); LCMS (ESI) calcd. for $C_{10}H_{14}NOS$ $[M+H]^+$: 196.1, found:196.0. This compound was known.[2]

S-(4-isopropylphenyl) dimethylcarbamothioate (**10**)

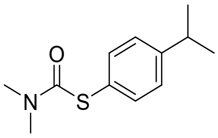

Yellow oil; yield 88%; UV λ_{max}(CH_2Cl_2/nm) 245; IR ν_{max}/cm^{-1} 1671(C=O); ^{1}H NMR (300 MHz, $CDCl_3$) δ 7.42-7.41 (m, 1H), 7.40-7.39 (m, 1H), 7.26-7.23(m, 2H), 3.05 (s, 6H), 2.94-2.89 (m, 1H), 1.26 (s, 3H), 1.23 (s, 3H); ^{13}C NMR (101 MHz, DMSO-*d*$_6$) δ 165.73, 150.01, 135.97, 127.43, 125.94, 36.92, 33.69, 24.19. LCMS (ESI) calcd. for $C_{12}H_{18}NOS$ $[M+H]^+$: 224.1, found: 224.0.

S-(4-(tert-butyl)phenyl) dimethylcarbamothioate (**11**)

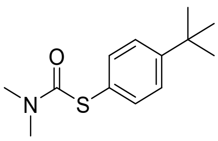

White solid; yield 77%; m. p.: 68.1~70.0°C; UV λ_{max}(CH_2Cl_2/nm) 242; IR ν_{max}/cm^{-1} 1662 (C=O); ^{1}H NMR (300 MHz, $CDCl_3$) δ 7.41 (s, 4H), 3.06 (s, 6H), 1.32 (*s*, 9H); LCMS (ESI) calcd. for $C_{13}H_{20}NOS$ $[M+H]^+$: 238.1, found: 238.0. This compound was known.[2]

S-(4-chlorophenyl) dimethylcarbamothioate (**12)**

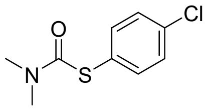

White solid; yield 79%; m.p. 76.5~79.7°C; UV λ_{max}(CH_2Cl_2/nm) 244; IR ν_{max}/cm^{-1} 1664(C=O); ^{1}H NMR (300 MHz, $CDCl_3$) δ 7.44-7.32 (m, 4H), 3.05 (s, 6H); LCMS (ESI) calcd. for $C_9H_{11}ClNOS$ $[M(^{35}Cl)+H]^+$: 216.0, found: 215.9. This compound was known.[2]

S-(3-chlorophenyl) dimethylcarbamothioate (**13)**

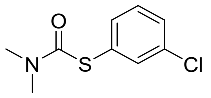

White solid; yield 78%; m.p. 46.0~48.3°C; UV λ_{max}(CH_2Cl_2/nm) 250; IR ν_{max}/cm^{-1} 1672(C=O); ^{1}H NMR (300 MHz, $CDCl_3$) δ 7.51-7.50 (m, 1H), 7.40-7.35 (m, 2H), 7.34-7.31 (m, 1H), 3.06 (s, 6H)·; ^{13}C NMR (101 MHz, DMSO-d_6) δ 164.63, 135.10, 134.33, 133.51, 131.35, 130.99, 129.58, 37.01.LCMS (ESI) calcd. for $C_9H_{11}ClNOS$ $[M(^{35}Cl)+H]^+$: 216.0, found: 215.9.

S-(2-chlorophenyl) dimethylcarbamothioate (**14)**

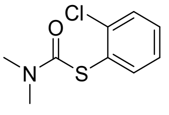

Colorless oil; yield 86%; UV λ_{max}(CH_2Cl_2/nm) 244; IR ν_{max}/cm^{-1} 1674 (C=O); ^{1}H NMR (300 MHz, $CDCl_3$) δ 7.60 (dd, *J*= 7.5, 1.9 Hz, 1H), 7.50 (dd, *J*=1.5, 8.0, Hz, 1H), 7.38-7.31(m, lH), 7.31-7.24 (m, 1H), 3.12 (s, 3H), 3.04 (s, 3H); LCMS (ESI) calcd. for $C_9H_{11}ClNOS$ $[M(^{35}Cl)+H]^+$: 216.0, found: 215.9. This compound was known.[3]

S-(2,4-dichlorophenyl) dimethylcarbamothioate (**15)**

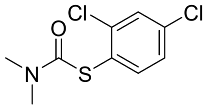

White solid; yield 86%; m.p. 65.7~66.9°C; UV λ_{max}(CH_2Cl_2/nm) 246; IR v_{max}/cm^{-1} 1667(C=O); ^{1}H NMR (300 MHz, $CDCl_3$) δ 7.54-7.52 (m, 2H), 7.52-7.50 (m, 1H), 7.28-7.24(m, 1H), 3.12 (s, 3H), 3.04 (s, 3H); ^{13}C NMR (101 MHz, DMSO-d_6) δ 163.42, 140.17, 139.85, 135.89, 129.93, 128.29, 127.76, 37.07.LCMS (ESI) calcd. for $C_9H_{10}Cl_2NOS$ [M(^{35}Cl, ^{35}Cl)+H]$^+$: 250.0, found: 249.90.

S-(4-cyanophenyl) dimethylcarbamothioate (**16**)

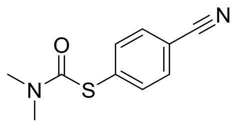

White solid; yield 75%; m.p. 101.0~102.4 °C; UV λ_{max}(CH_2Cl_2/nm) 244; IR v_{max}/cm^{-1} 1672(C=O); ^{1}H NMR (300 MHz, $CDCl_3$) δ 7.67-7.60 (m, 4H), 3.10 (s, 3H), 3.05 (s, 3H); LCMS (ESI) calcd. for $C_{13}H_{20}NOS$ [M+H]$^+$: 207.1, found: 207.0. This compound was known.[4]

S-(4-nitrophenyl) dimethylcarbamothioate (**17**)

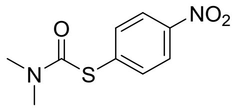

Pale yellow solid; yield 81%; m.p. 117.0~118.4 °C; UV λ_{max}(CH_2Cl_2/nm) 305; IR v_{max}/cm^{-1} 1675 (C=O); ^{1}H NMR (300 MHz, $CDCl_3$) δ 8.24-8.20 (m, 2H), 7.70-7.66 (m, 2H), 3.12 (s, 3H), 3.06 (s, 3H); LCMS (ESI) calcd. for $C_9H_{11}N_2O_3$ [M+H]$^+$: 227.0, found: 227.0. This compound was known.[5]

S-(4-methoxyphenyl) dimethylcarbamothioate (**18**)

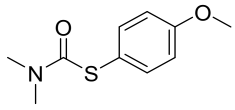

White solid; yield 80%; m.p. 92.1~94.2 °C; UV λ_{max}(CH_2Cl_2/nm) 243; IR v_{max}/cm^{-1}1670(C=O); ^{1}H NMR (300 MHz, $CDCl_3$) δ 7.42-7.37 (m, 2H), 6.94-6.89 (m, 2H), 3.82 (s, 3H), 3.05 (s, 6H); LCMS (ESI) calcd. for $C_{10}H_{14}NO_2S$ [M+H]$^+$: 212.1, found: 212.0. This compound was known.[2]

Methyl 4-((dimethylcarbamoyl)thio)benzoate (**19**)

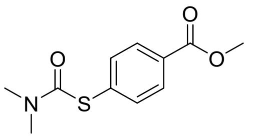

Pale yellow solid; yield 78%; m.p. 92.2~94.7 °C; UV $\lambda_{max}(CH_2Cl_2/nm)$ 244; IR ν_{max}/cm^{-1} 1667(C=O); ^1H NMR (300 MHz, $CDCl_3$) δ 8.05-8.02 (m, 2H), 7.59-7.56 (m, 2H), 3.92 (s, 3H), 3.10 (s, 3H), 3.04 (s, 3H); LCMS (ESI) calcd. for $C_{11}H_{14}NO_3S$ $[M+H]^+$: 240.1, found: 239.90. This compound was known.[5]

Compounds **2** and **20~32** were synthesized according to the procedure described for General method ii.

4-Fluorobenzenesulfonyl fluoride (**2**)

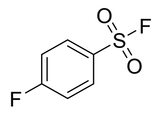

Colorless oil; yield 68%; UV $\lambda_{max}(CH_2Cl_2/nm)$ 232; IR ν_{max}/cm^{-1} 1417, 1214 (SO_2); ^1H NMR (300 MHz, $CDCl_3$) δ 8.10-8.03 (m, 2H), 7.35-7.29 (m, 2H); ^{19}F-NMR (300MHz, $CDCl_3$) δ 66.79 (s, 1F), -99.32 (s, 1F); LCMS (ESI) calcd. for $C_6H_5F_2O_2S$ $[M+H]^+$: 179.0, found: 179.1. This compound was known.[6]

4-Methylbenzenesulfonyl fluoride (**20**)

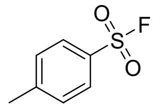

White solid; yield 68%; m.p. 40.1~42.0 °C; UV $\lambda_{max}(CH_2Cl_2/nm)$ 236; IR ν_{max}/cm^{-1} 1409,1216 (SO_2); ^1H NMR (300 MHz, $CDCl_3$) δ 7.92-7.89 (m, 2H), 7.45-7.41 (m, 2H), 2.50 (s, 3H); ^{19}F NMR (300 MHz, $CDCl_3$) δ 66.30 (s, 1F); LCMS (ESI) calcd. for $C_7H_8FO_2S$ $[M+H]^+$: 175.0, found: 174.9. This compound was known.[6]

3-Methylbenzenesulfonyl fluoride (**21**)

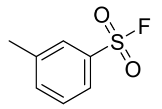

Colorless oil; yield 79%; UV $\lambda_{max}(CH_2Cl_2/nm)$ 231; IR ν_{max}/cm^{-1} 1461, 1261 (SO_2); ^1H NMR (300 MHz, $CDCl_3$) δ 7.82 -7.81 (m, 2H),7.60-7.57 (m, 2H), 7.54-7.49 (m, 1H), 2.48 (s,3H); ^{19}F NMR (300 MHz, $CDCl_3$) δ 65.83 (s, 1F); LCMS (ESI) calcd. for $C_7H_8FO_2S$ $[M+H]^+$: 175.0, found: 174.9. This compound was known.[7]

2-Methylbenzenesulfonyl fluoride (**22**)

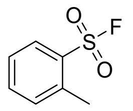

Colorless oil; yield 79%; UV λ_{max}(CH_2Cl_2/nm) 228; IR ν_{max}/cm^{-1} 1402, 1212 (SO_2); ^1H NMR (300 MHz, $CDCl_3$) δ 8.04 (d, *J*= 7.7 Hz, 1H), 7.66-7.60 (m, 1H), 7.45-7.38 (m, 2H), 2.70 (s, 3H); ^{19}F NMR (300 MHz, $CDCl_3$) δ 60.26 (s, 1F); LCMS (ESI) calcd. for $C_7H_8FO_2S$ $[M+H]^+$: 175.0, found: 174.9. This compound was known.[7]

4-Isopropylbenzenesulfonyl fluoride (**23)**

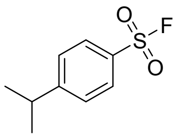

Colorless oil; yield 79%; UV λ_{max}(CH_2Cl_2/nm) 238; IR ν_{max}/cm^{-1} 1408, 1214 (SO_2); ^1H NMR (300 MHz, $CDCl_3$) δ 7.96-7.92 (m, 2H), 7.50-7.46 (m, 2H), 3.02-2.97 (m, 1H), 1.31 (s, 3H), 1.29 (s, 3H); ^{19}F NMR (300 MHz, $CDCl_3$) δ 66.26 (s, 1F); LCMS (ESI) calcd. for $C_9H_{12}FO_2S$ $[M+H]^+$: 203.1, found: 202.9. This compound was known.[8]

4-(Tert-butyl)benzenesulfonyl fluoride (**24)**

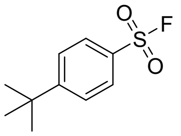

White solid; yield 77%; m.p. 63.9~65.7 °C; UV λ_{max}(CH_2Cl_2/nm) 240; IR ν_{max}/cm^{-1} 1400, 1214 (SO_2); ^1H NMR (300 MHz, $CDCl_3$) δ 7.96-7.92 (m, 2H), 7.66-7.62 (m, 2H), 1.37 (s, 9H); ^{19}F NMR (300 MHz, $CDCl_3$) δ 66.13 (s, 1F); LCMS (ESI) calcd. for $C_{10}H_{14}FO_2S$ $[M+H]^+$: 217.1, found: 216.8. This compound was known.[7]

4-Chlorobenzenesulfonyl fluoride (**25)**

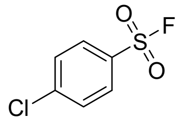

White solid; yield 90%; m.p. 47.5~49.1 °C; UV λ_{max}(CH_2Cl_2/nm) 239; IR ν_{max}/cm^{-1} 1413, 1214 (SO_2); ^1H NMR (300 MHz, $CDCl_3$) δ 7.99-7.95 (m, 2H), 7.65-7.61 (m, 2H); ^{19}F NMR (300 MHz, $CDCl_3$) δ 66.55 (s, 1F); LCMS (ESI) calcd. for C_6H_5ClFOS $[M(^{35}Cl)-O+H]^+$ 179.0, found: 179.0. This compound was known.[6]

3-Chlorobenzenesulfonyl fluoride (**26**)

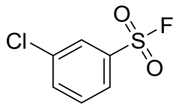

Colorless oil; yield 57%; UV λ_{max}(CH_2Cl_2/nm) 233; IR v_{max}/cm^{-1} 1416, 1214 (SO_2); ^{1}H NMR (300 MHz, $CDCl_3$) δ 8.02-7.99 (m, 1H), 7.94-7.90 (m, 1H), 7.78-7.74 (m, 1H), 7.63-7.57 (m, 1H); ^{19}F NMR (300 MHz, $CDCl_3$) δ 66.13 (s, 1F); LCMS (ESI) calcd. for C_6H_5ClFOS [M(^{35}Cl)-O+H]$^+$ 179.0, found: 178.8. This compound was known.[7]

2-Chlorobenzenesulfonyl fluoride (**27**)

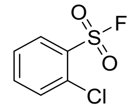

White solid; yield 68%; m.p. 36.4~38.1 °C; UV λ_{max}(CH_2Cl_2/nm) 230; IR v_{max}/cm^{-1} 1411, 1216 (SO_2); ^{1}H NMR (300 MHz, $CDCl_3$) δ 8.12 (dd, *J*= 8.0, 1.4 Hz, 1H), 7.70 (td, *J*= 8.0, 1.4 Hz, 1H), 7.67-7.63 (m, 1H), 7.55-7.48 (m, 1H); ^{19}F NMR (300 MHz, $CDCl_3$) δ 58.88 (s, 1F); LCMS (ESI) calcd. for C_6H_5ClFOS [M(^{35}Cl)-O+H]$^+$ 179.0, found: 179.0. This compound was known.[9]

2,4-Dichlorobenzenesulfonyl fluoride (**28**)

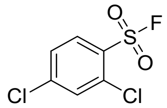

White solid; yield 89%; m.p. 55.8~58.4 °C; UV λ_{max}(CH_2Cl_2/nm) 240; IR v_{max}/cm^{-1} 1409, 1209 (SO_2); ^{1}H NMR (300 MHz, $CDCl_3$) δ 8.06 (d, *J*= 8.6 Hz, 1H), 7.66 (d, *J*= 2.0 Hz, 1H), 7.50 (dt, *J*= 8.6, 2.0 Hz, 1H); ^{19}F NMR (300 MHz, $CDCl_3$) δ 59.62 (s, 1F); LCMS (ESI) calcd. for C_6H_5ClFOS [M(^{35}Cl)-Cl-O+2H]$^+$ 179.0, found: 179.0. This compound was known.[10]

4-Cyanobenzenesulfonyl fluoride (**29**)

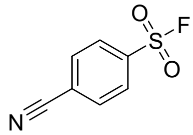

White solid; yield 73%; m.p. 55.8~58.4 °C; UV λ_{max}(CH_2Cl_2/nm) 239; IR v_{max}/cm^{-1} 1413, 1214 (SO_2); ^{1}H NMR (300 MHz, $CDCl_3$) δ 8.18-8.15 (m, 2H), 7.97-7.94 (m,

2H); ^{19}F NMR (300 MHz, $CDCl_3$) δ 66.02 (s, 1F); LCMS (ESI) calcd. for C_7H_5NOS $[M\text{-}F\text{-}O+H]^+$ 151.0, found: 151.0. This compound was known.[7]

4-Nitrobenzenesulfonyl fluoride (**30)**

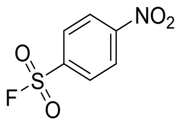

White solid; yield 79%; m.p. 76.7~77.7 °C; UV λ_{max}(CH_2Cl_2/nm) 245; IR ν_{max}/cm^{-1} 1419, 1216 (SO_2); ^{1}H NMR (300 MHz, $CDCl_3$) δ 8.50 (d, *J*= 8.9 Hz, 2H), 8.26 (d, *J*= 8.9 Hz, 2H); ^{19}F NMR (300 MHz, $CDCl_3$) δ 66.25 (s, 1F); LCMS (ESI) calcd. for $C_6H_4NO_2S$ $[M\text{-}F\text{-}2O]^+$ 154.0, found: 154.0. This compound was known.[6]

4-Methoxybenzenesulfonyl fluoride (**31)**

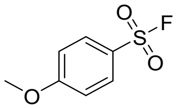

Colorless oil; yield 72%; UV λ_{max}(CH_2Cl_2/nm) 247; IR ν_{max}/cm^{-1} 1404, 1212 (SO_2); ^{1}H NMR (300 MHz, $CDCl_3$) δ 7.94 (d, *J*= 9.0 Hz, 2H), 7.07 (d, *J*= 9.0 Hz, 2H), 3.92 (s, 3H); ^{19}F NMR (300 MHz, $CDCl_3$) δ 67.32 (s, 1F); LCMS (ESI) calcd. for $C_7H_6FO_3S$ $[M\text{-}H]^-$: 189.0, found: 188.9. This compound was known.[6]

Methyl 4-(fluorosulfonyl)benzoate (**32)**

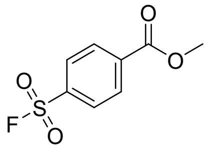

White solid; yield 81%; m.p. 82.2~84.3 °C; UV λ_{max}(CH_2Cl_2/nm) 239; IR ν_{max}/cm^{-1} 1411, 1212 (SO_2); ^{1}H NMR (300 MHz, $CDCl_3$) δ 8.30-8.27 (m, 2H), 8.12-8.09 (m, 2H), 4.00 (s, 3H); ^{19}F NMR (300 MHz, $CDCl_3$) δ 65.80 (s, 1F); LCMS (ESI) calcd. for $C_8H_8FO_4S$ $[M+H]^+$: 219.0, found: 219.0. This compound was known.[10]

Analytical Equipment

Melting points were measured using a Stuart automatic melting point SMP40 apparatus or a Shanghai ShenGuang WRR apparatus. Fourier Transform InfraRed (FTIR) spectra were measured using an Agilent Cary 630 FTIR or a Bruker TENSOR II FTIR. All solvents were used after appropriate distillation or purification. ^{1}H NMR and ^{13}C NMR spectra were recorded with Bruker Advance 300/400/500 MHz

spectrometer (Bruker Company, Germany) in the indicated sol vents ($CDCl_3$ or DMSO-d_6, tetramethylsilane (TMS) as internal stan dard): the values of the chemical shifts are expressed in δ values (ppm) and the coupling constants (*J*) in hertz. High-resolution mass spectra (HRMS) were measured with an Aglilent Technologies 6538 UHD Accurate-Mass Q-TOF MS spectrometer using ESI.

LC-MS analyses were conducted using a Waters Acquity UPLC system with photo diode array (PDA) and evaporating light scattering detector (ELSD) or using the ESI mass spectra which were performed by Zichao Ding on an Agilent Technologies 6120 Quadrupole LC-MS. When a 2 min gradient was used, the sample was eluted on an Acquity UPLC BEH C18, 1.7μm, 2.1 x 50mm, with a flow rate of 1 ml/min using 10-100% 0.1% trifluoroacetic acid in MeCN. Analytical purity of compounds was determined using Waters XTerra RP18, 5 μm (4.6 × 150 mm) column at 1 ml/min using either 0.1% aq. and MeCN or 0.1% aq. trifluoroacetic acid and MeCN with a gradient of 10-100% over 20 min. When a 12 min gradient was used, the sample was eluted on ZORBAX Eclipse XDB-C18, 3.5 μm, 4.6 x 100 mm, with a flow rate of 1 ml/min using 30-70% 0.1% trifluoroacetic acid in MeCN.

Compound **C1** was dissolved into MeCN and evaporated slowly under 4 ^{o}C to give colourless needle crystals. The grown crystals were collected and identified as No. **250116b**. A specimen of **250116b**, approximate dimensions 0.030 mm x 0.040 mm x 0.200 mm, was used for the X-ray crystallographic analysis. The X-ray intensity data were measured on a Bruker D8 VENTURE DUO PHOTON III system equipped with a Incoatec Ius 3.0 Microfocus sealed tube (Cu Kα, λ = 1.54178 Å) and a Helios MX Multilayer Optic monochromator. The structure was solved and refined using the Bruker SHELXTL Software Package. Data was shown in Table S1 and S2.

Table 1. Sample and crystal data for 250116b.

**Identification code**	250116b
**Chemical formula**	$C_{17}H_{12}F_2O_4S$
**Formula weight**	350.33 g/mol
**Temperature**	150(2) K
**Wavelength**	1.54178 Å
**Crystal size**	0.030 x 0.040 x 0.200 mm

**Crystal system**	monoclinic
**Space group**	P 1 21/c 1
**Unit cell dimensions**	a = 6.8190(8) Å α = 90°
	b = 14.7869(15) Å β = 91.279(6)°
	c = 14.7647(15) Å γ = 90°
**Volume**	1488.4(3) $Å^3$
**Z**	4
**Density (calculated)**	1.563 g/cm^3
**Absorption coefficient**	2.343 mm^{-1}
**F(000)**	720

Table 2. Data collection and structure refinement for 250116b.

**Diffractometer**	Bruker D8 VENTURE DUO PHOTON III	
**Radiation source**	Incoatec Ius 3.0 Microfocus sealed tube (Cu Kα, λ = 1.54178 Å)	
**Theta range for data collection**	4.23 to 66.96°	
**Index ranges**	-8<=h<=8, -17<=k<=17, -17<=l<=14	
**Reflections collected**	16460	
**Independent reflections**	2595 [R(int) = 0.0725]	
**Coverage of independent reflections**	97.9%	
**Absorption correction**	Multi-Scan	
**Max. and min. transmission**	0.9330 and 0.6520	
**Structure solution technique**	direct methods	
**Structure solution program**	XT, VERSION 2018/2	
**Refinement method**	Full-matrix least-squares on F^2	
**Refinement program**	SHELXL-2019/1 (Sheldrick, 2019)	
**Function minimized**	Σ w(F_o^2 - F_c^2)^2	
**Data / restraints / parameters**	2595 / 0 / 218	
**Goodness-of-fit on F^2**	1.076	
**Final R indices**	2316 data; I>2σ(I)	R1 = 0.1209, wR2 = 0.3603
	all data	R1 = 0.1261, wR2 = 0.3626
**Weighting scheme**	w=1/[$\sigma^2(F_o^2)$+(0.1940P)^2+11.1720P] where P=(F_o^2+2F_c^2)/3	
**Largest diff. peak and hole**	1.518 and -0.564 $eÅ^{-3}$	

**R.M.S. deviation from mean**	0.157 eÅ^{-3}

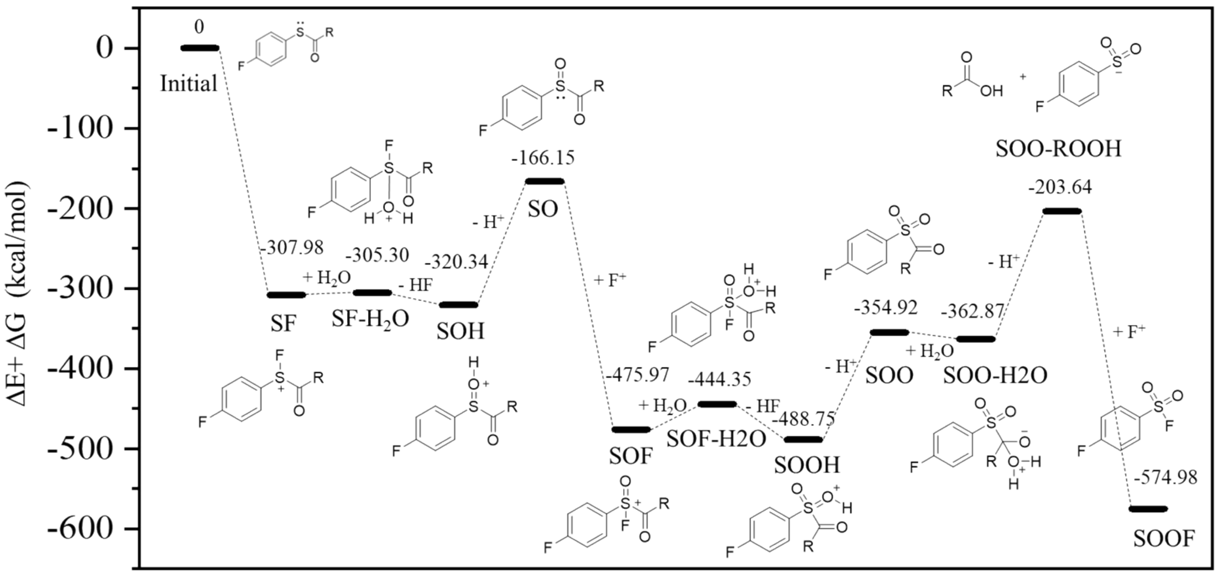

Figure S1 Free energy changes of the possible reaction pathways. Calculations were performed in the framework of the density functional theory with the B3LYP method. The 6-311g (d, p) basis set is used for C, H, O, N, F and S atoms. The optimal structure was calculated using PCM solvent model in water and acetonitrile solution (1:1). The sum of Gibbs free energy (ΔG) and Electronic Energy (ΔE) are used in reaction.

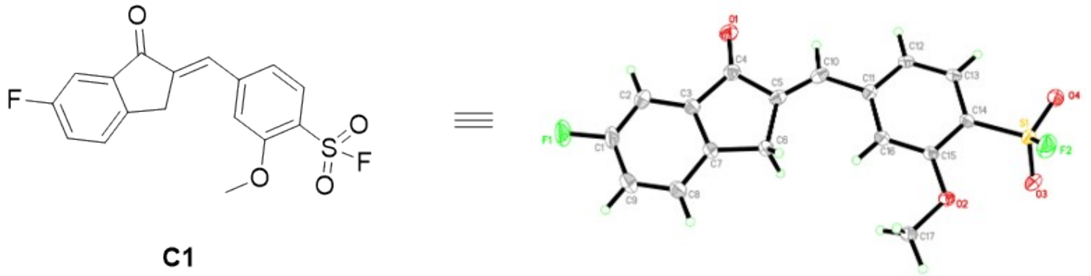

C1

Figure S2 X-ray crystal structure of compound **C1**. The stereochemistry reflects the E geometry of the double bond.

NMR, HRMS, HPLC spectra

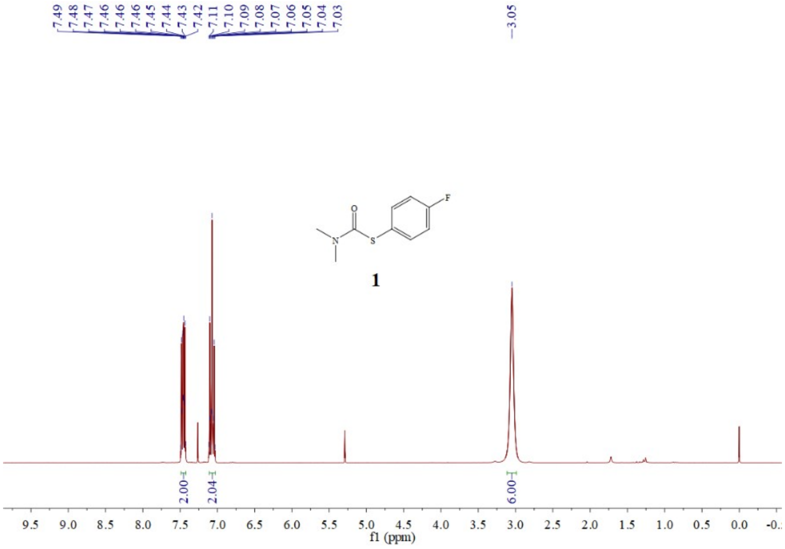

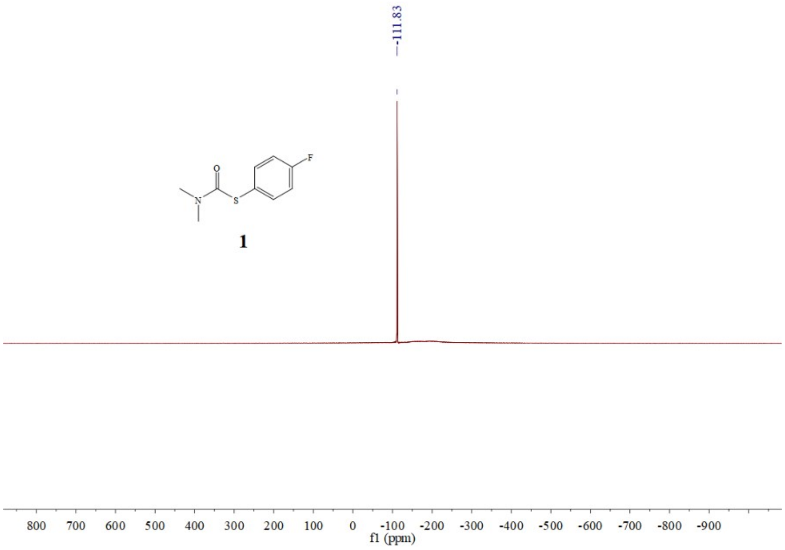

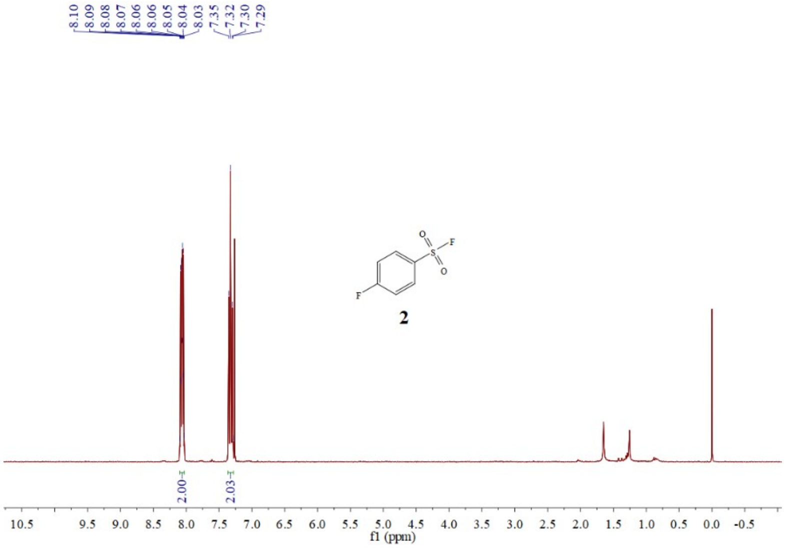

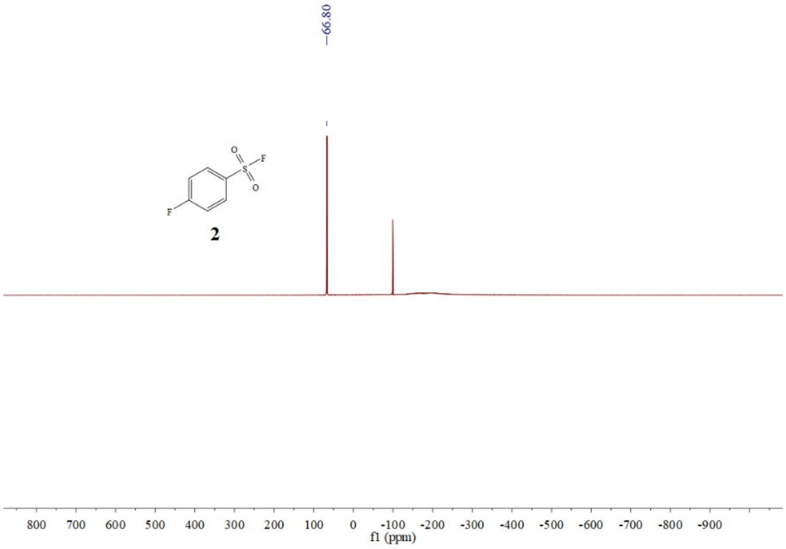

66.80
2
800 700 600 500 400 300 200 100 0 -100 -200 -300 -400 -500 -600 -700 -800 -900
f1 (ppm)

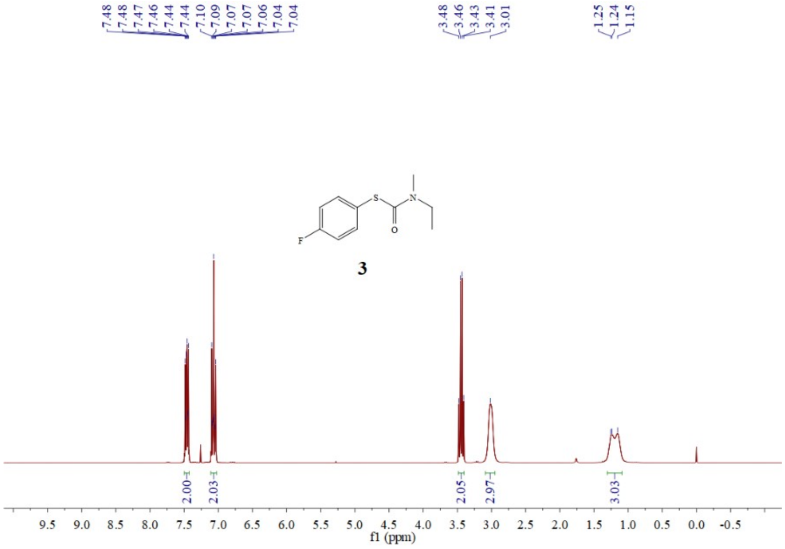

7.48 7.48 7.47 7.46 7.44 7.44 7.10 7.09 7.07 7.07 7.06 7.04 7.04
3.48 3.46 3.43 3.41 3.01
1.25 1.24 1.15
3
2.00 2.03 2.05 2.97 3.03
9.5 9.0 8.5 8.0 7.5 7.0 6.5 6.0 5.5 5.0 4.5 4.0 3.5 3.0 2.5 2.0 1.5 1.0 0.5 0.0 -0.5
f1 (ppm)

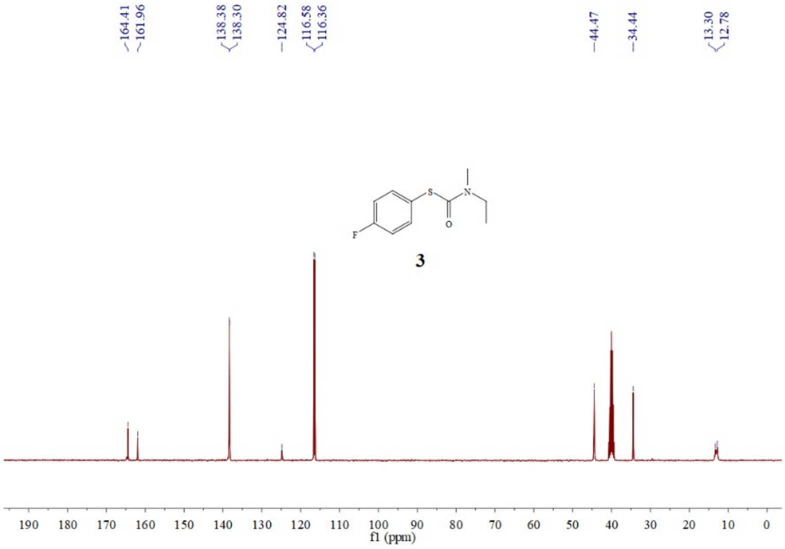

164.41 161.96
138.38 138.30
124.82
116.58 116.36
44.47
34.44
13.30 12.78
3
190 180 170 160 150 140 130 120 110 100 90 80 70 60 50 40 30 20 10 0
f1 (ppm)

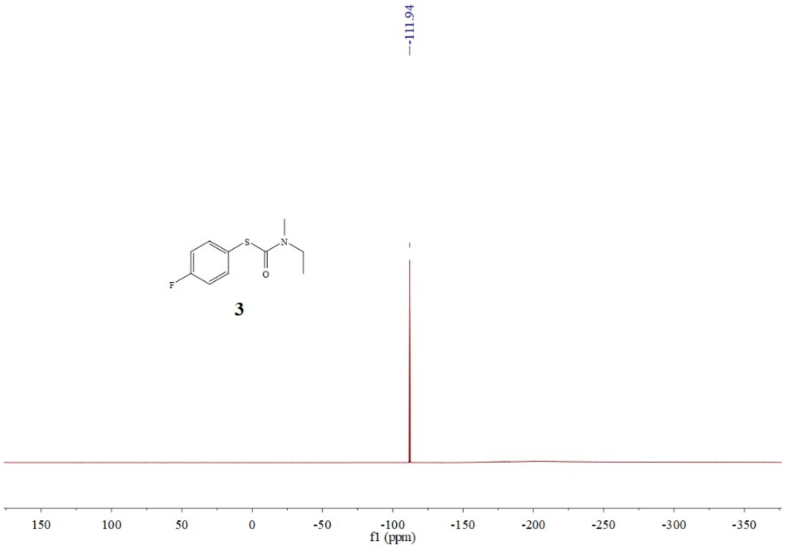
-111.94
3
150 100 50 0 -50 -100 -150 -200 -250 -300 -350
f1 (ppm)

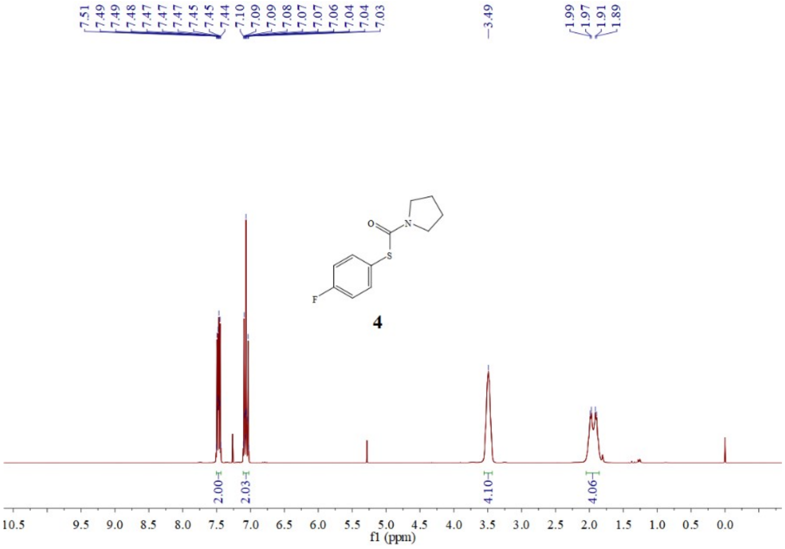
7.51 7.49 7.49 7.48 7.47 7.47 7.47 7.45 7.45 7.44 7.10 7.09 7.09 7.08 7.07 7.07 7.06 7.04 7.04 7.03
3.49
1.99 1.97 1.91 1.89
4
2.00 2.03 4.10 4.06
10.5 9.5 9.0 8.5 8.0 7.5 7.0 6.5 6.0 5.5 5.0 4.5 4.0 3.5 3.0 2.5 2.0 1.5 1.0 0.5 0.0
f1 (ppm)

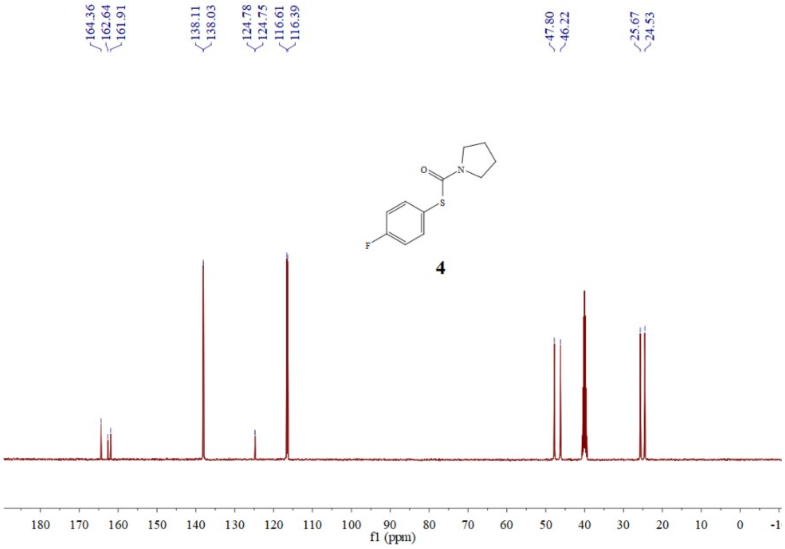
164.36 162.64 161.91
138.11 138.03
124.78 124.75 116.61 116.39
47.80 46.22
25.67 24.53
4
180 170 160 150 140 130 120 110 100 90 80 70 60 50 40 30 20 10 0 -1
f1 (ppm)

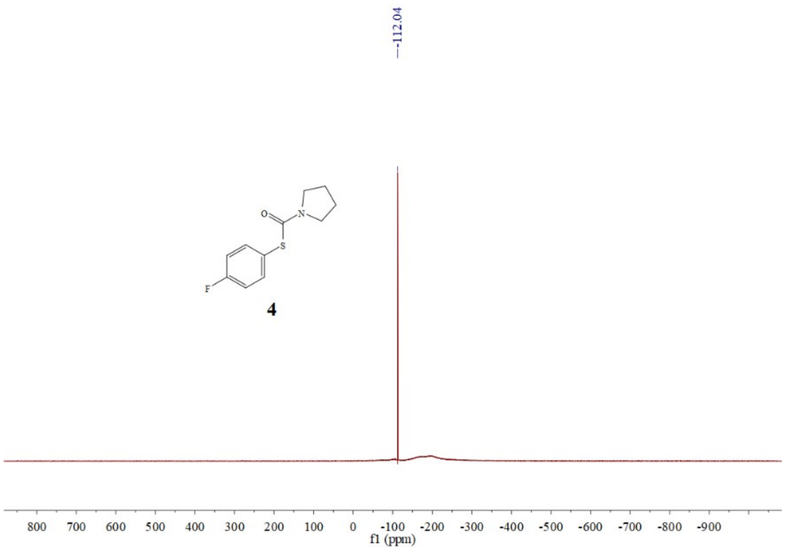
-112.04
O
N
S
F
4
800 700 600 500 400 300 200 100 0 -100 -200 -300 -400 -500 -600 -700 -800 -900
f1 (ppm)

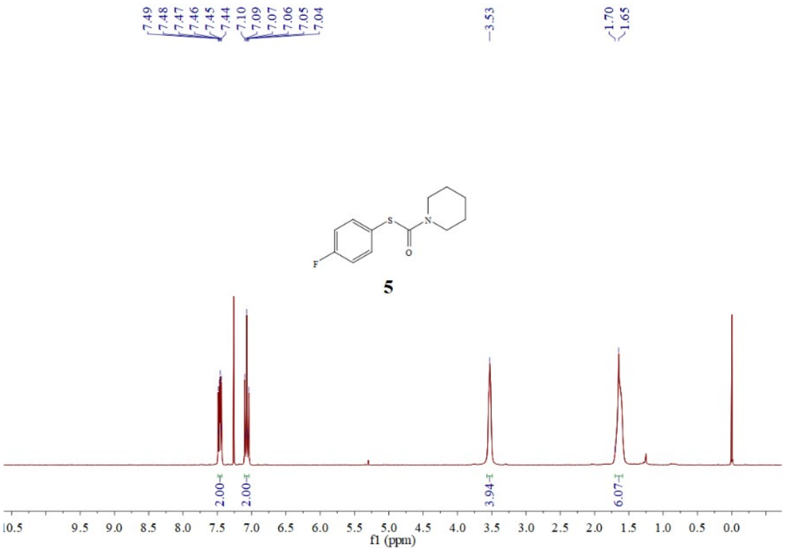
7.49 7.48 7.47 7.46 7.45 7.44 7.10 7.09 7.07 7.06 7.05 7.04
3.53
1.70 1.65
S
N
F
O
5
2.00 2.00 3.94 6.07
10.5 9.5 9.0 8.5 8.0 7.5 7.0 6.5 6.0 5.5 5.0 4.5 4.0 3.5 3.0 2.5 2.0 1.5 1.0 0.5 0.0
f1 (ppm)

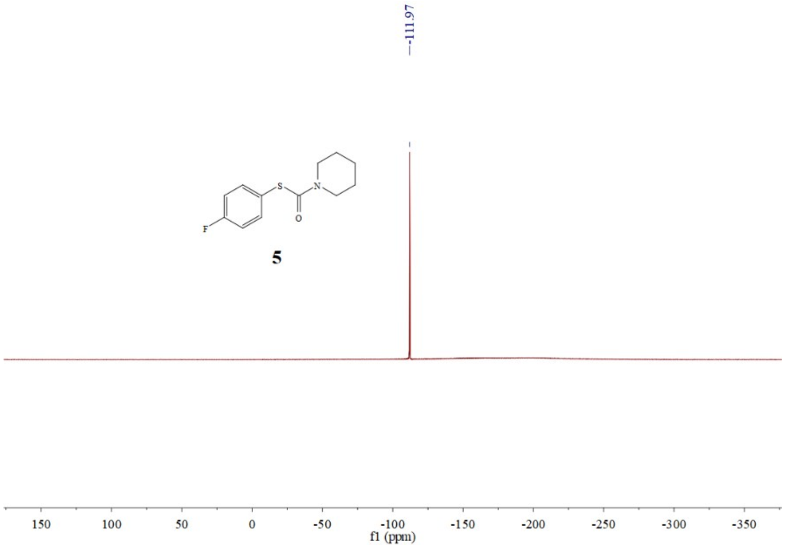
-111.97
S
N
F
O
5
150 100 50 0 -50 -100 -150 -200 -250 -300 -350
f1 (ppm)

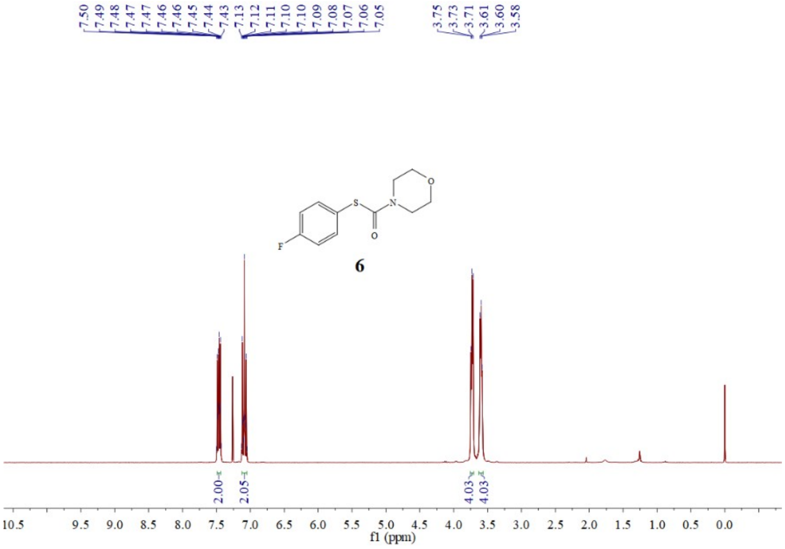
7.50
7.49
7.48
7.47
7.47
7.46
7.45
7.44
7.43
7.13
7.12
7.11
7.10
7.10
7.09
7.08
7.07
7.06
7.05
3.75
3.73
3.71
3.61
3.60
3.58
F
S
N
O
O
6
2.00
2.05
4.03
4.03
10.5 9.5 9.0 8.5 8.0 7.5 7.0 6.5 6.0 5.5 5.0 4.5 4.0 3.5 3.0 2.5 2.0 1.5 1.0 0.5 0.0
f1 (ppm)

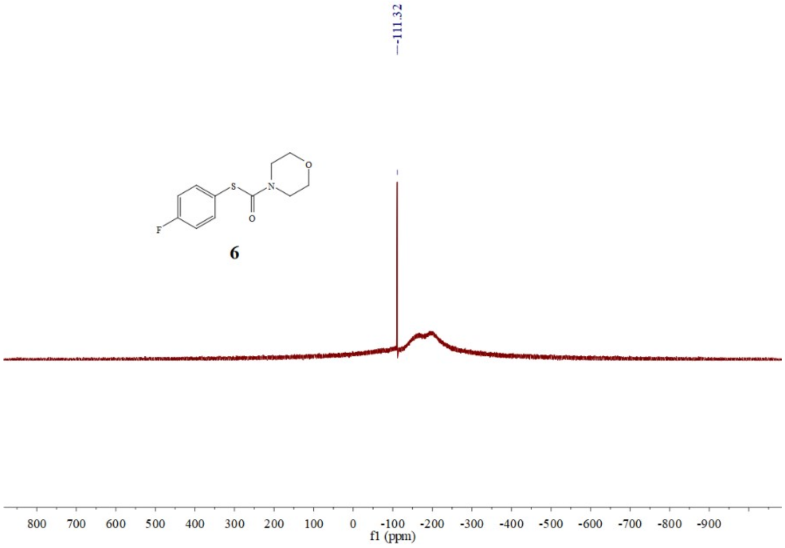
-111.32
F
S
N
O
O
6
800 700 600 500 400 300 200 100 0 -100 -200 -300 -400 -500 -600 -700 -800 -900
f1 (ppm)

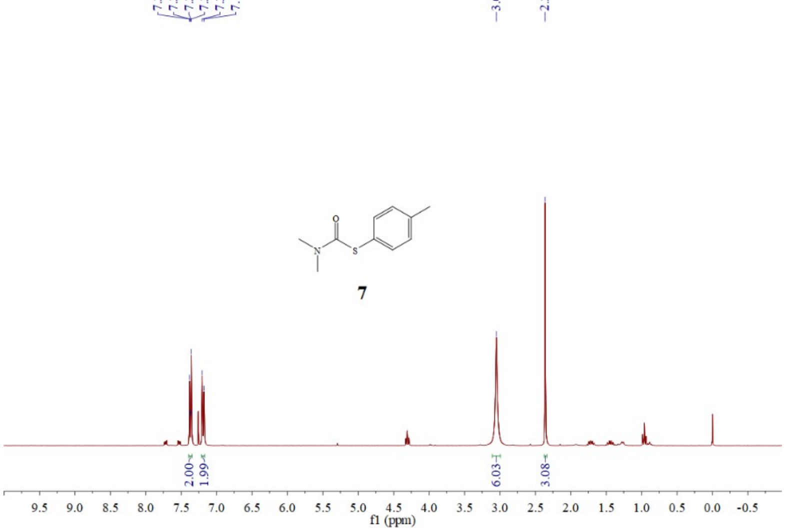
7.38
7.38
7.36
7.36
7.20
7.18
3.05
2.36
O
N
S
7
2.00
1.99
6.03
3.08
9.5 9.0 8.5 8.0 7.5 7.0 6.5 6.0 5.5 5.0 4.5 4.0 3.5 3.0 2.5 2.0 1.5 1.0 0.5 0.0 -0.5
f1 (ppm)

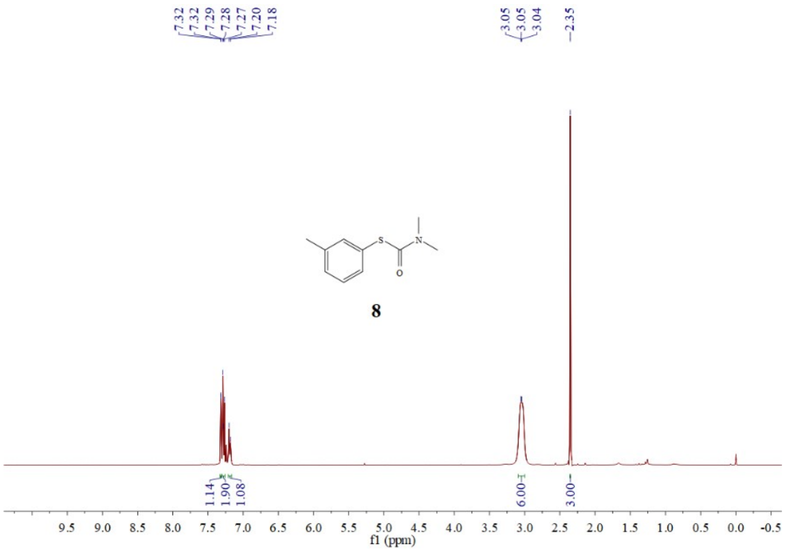
7.32
7.32
7.29
7.28
7.27
7.20
7.18
3.05
3.05
3.04
2.35
S
N
O
8
1.14
1.90
1.08
6.00
3.00
9.5 9.0 8.5 8.0 7.5 7.0 6.5 6.0 5.5 5.0 4.5 4.0 3.5 3.0 2.5 2.0 1.5 1.0 0.5 0.0 -0.5
f1 (ppm)

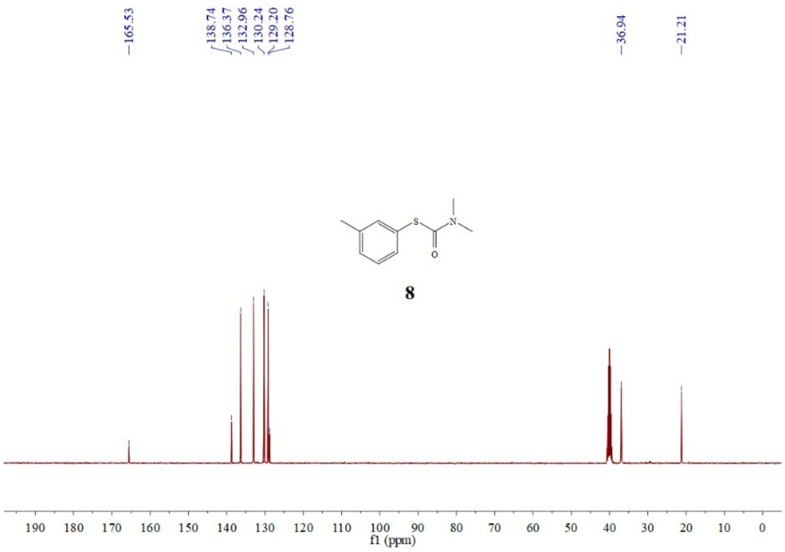
165.53
138.74
136.37
132.96
130.24
129.20
128.76
36.94
21.21
S
N
O
8
190 180 170 160 150 140 130 120 110 100 90 80 70 60 50 40 30 20 10 0
f1 (ppm)

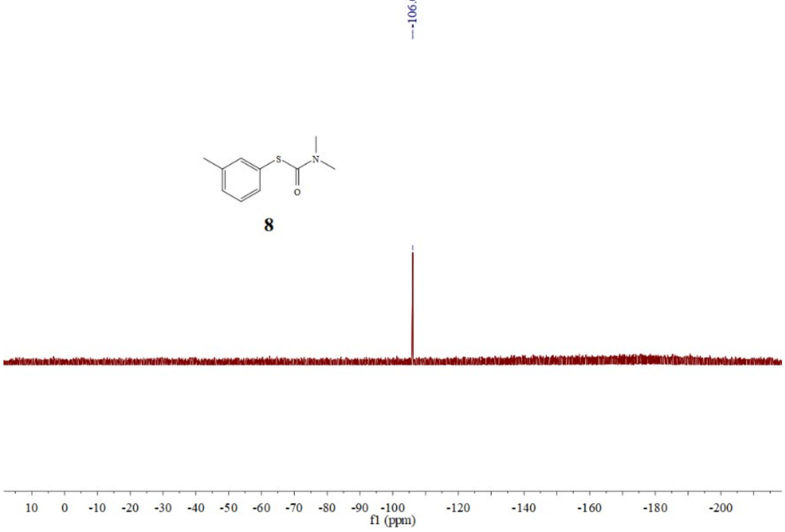
-106.09
S
N
O
8
10 0 -10 -20 -30 -40 -50 -60 -70 -80 -90 -100 -120 -140 -160 -180 -200
f1 (ppm)

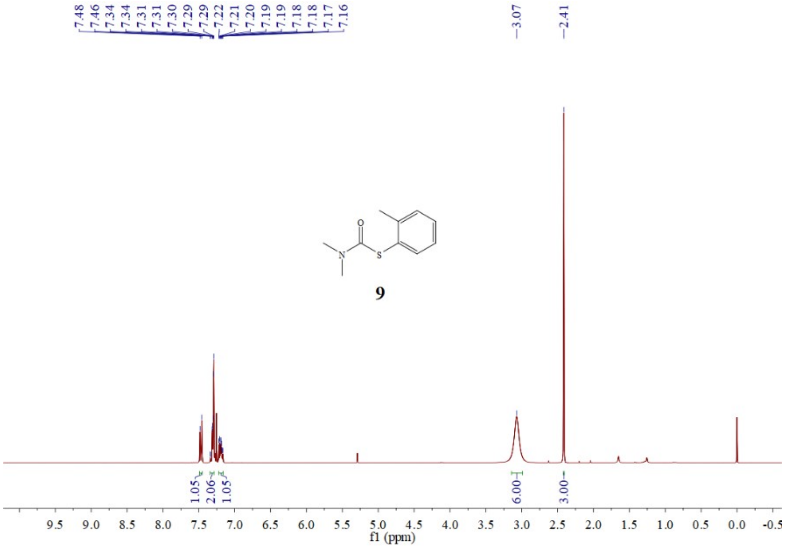
7.48
7.46
7.34
7.34
7.31
7.31
7.30
7.29
7.29
7.22
7.21
7.20
7.19
7.19
7.18
7.18
7.17
7.16
3.07
2.41
9
1.05
2.06
1.05
6.00
3.00
9.5 9.0 8.5 8.0 7.5 7.0 6.5 6.0 5.5 5.0 4.5 4.0 3.5 3.0 2.5 2.0 1.5 1.0 0.5 0.0 -0.5
f1 (ppm)

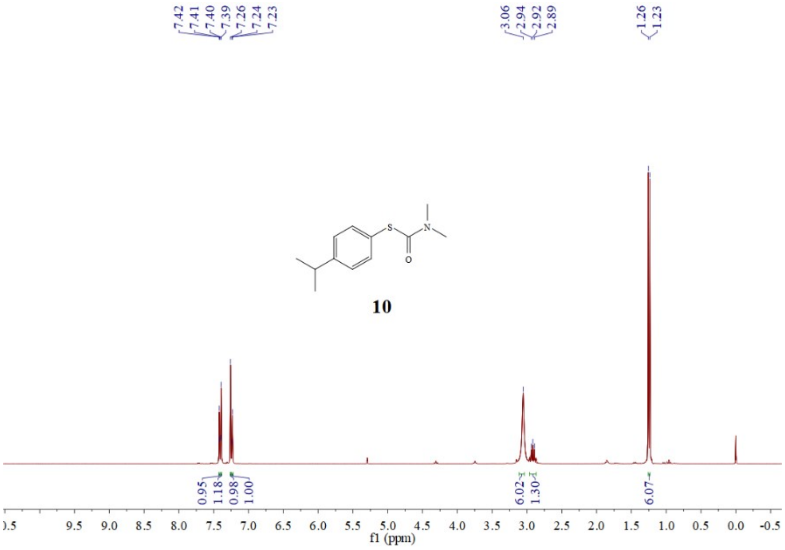
7.42
7.41
7.40
7.39
7.26
7.24
7.23
3.06
2.94
2.92
2.89
1.26
1.23
10
0.95
1.18
0.98
1.00
6.02
1.30
6.07
9.5 9.0 8.5 8.0 7.5 7.0 6.5 6.0 5.5 5.0 4.5 4.0 3.5 3.0 2.5 2.0 1.5 1.0 0.5 0.0 -0.5
f1 (ppm)

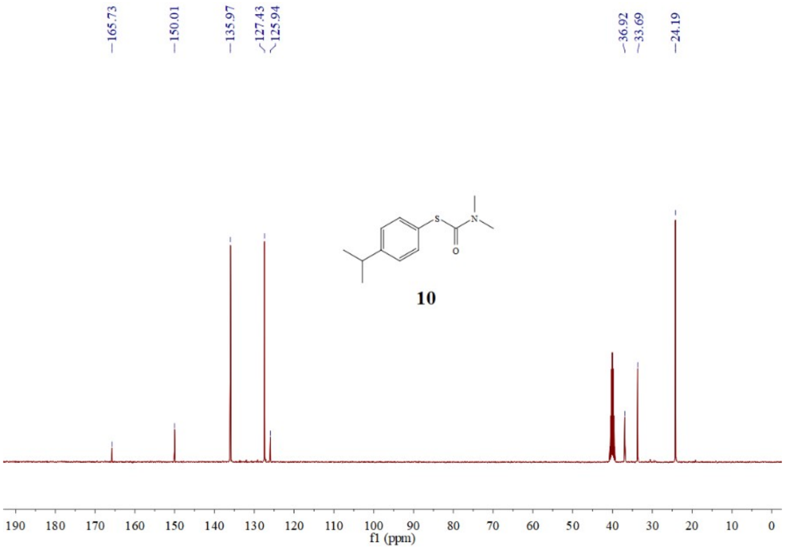
165.73
150.01
135.97
127.43
125.94
36.92
33.69
24.19
10
190 180 170 160 150 140 130 120 110 100 90 80 70 60 50 40 30 20 10 0
f1 (ppm)

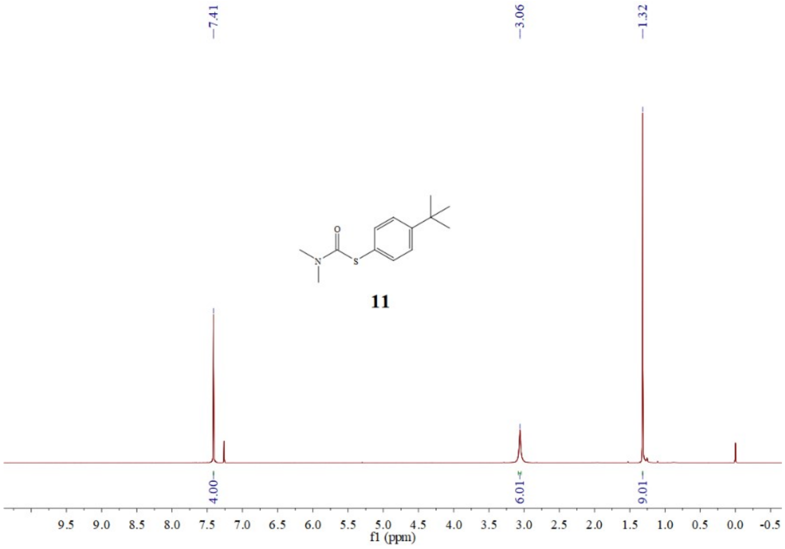

7.41
3.06
1.32
11
4.00
6.01
9.01
f1 (ppm)

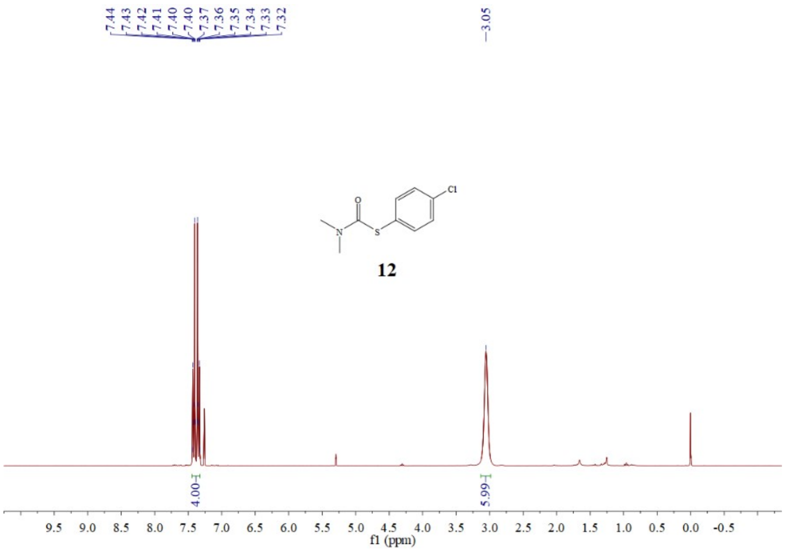

7.44 7.43 7.42 7.41 7.40 7.40 7.37 7.36 7.35 7.34 7.33 7.32
3.05
Cl
12
4.00
5.99
f1 (ppm)

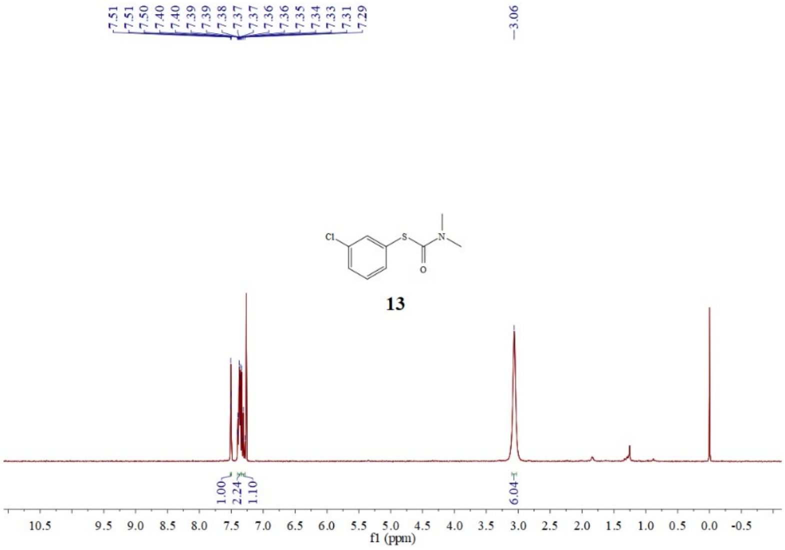

7.51 7.51 7.50 7.40 7.40 7.39 7.39 7.38 7.37 7.37 7.36 7.36 7.35 7.34 7.33 7.31 7.29
3.06
Cl
13
1.00
2.24
1.10
6.04
f1 (ppm)

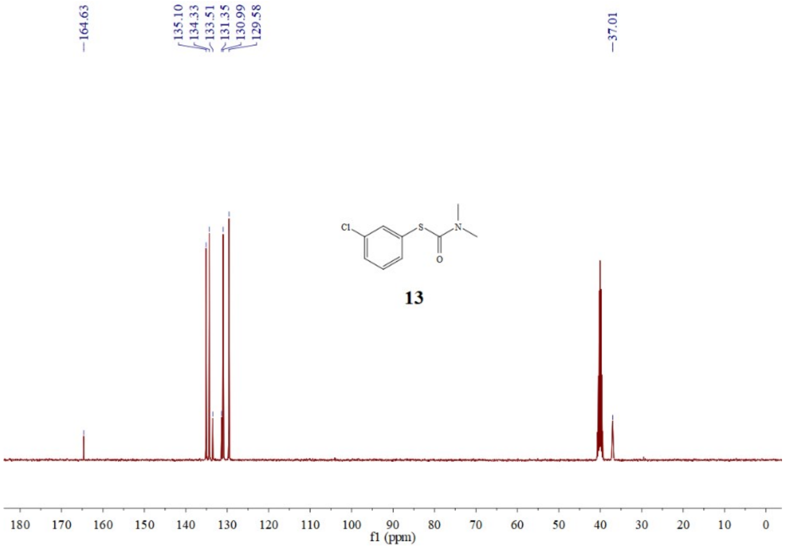
164.63
135.10
134.33
133.51
131.35
130.99
129.58
37.01
Cl
S
N
O
180 170 160 150 140 130 120 110 100 90 80 70 60 50 40 30 20 10 0
f1 (ppm)

13

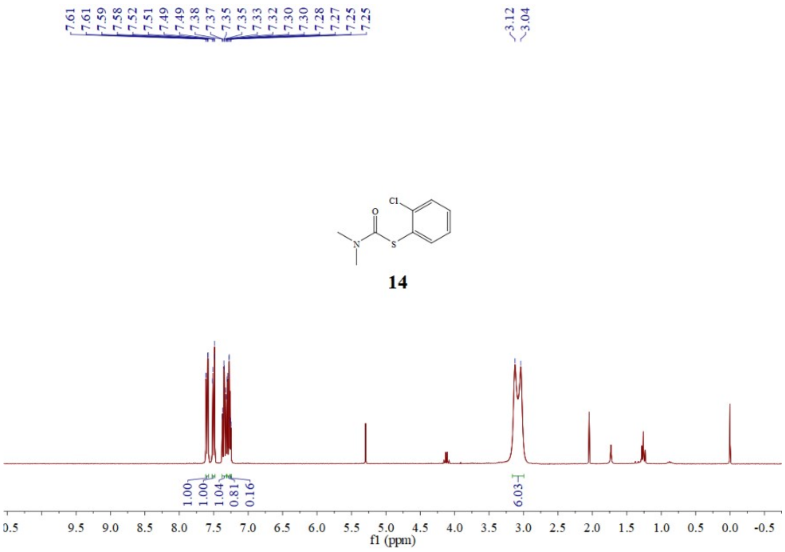
7.61
7.61
7.59
7.58
7.52
7.51
7.49
7.49
7.38
7.37
7.35
7.35
7.33
7.32
7.30
7.30
7.28
7.27
7.25
7.25
3.12
3.04
Cl
O
N
S
1.00
1.00
1.04
0.81
0.16
6.03
0.5 9.5 9.0 8.5 8.0 7.5 7.0 6.5 6.0 5.5 5.0 4.5 4.0 3.5 3.0 2.5 2.0 1.5 1.0 0.5 0.0 -0.5
f1 (ppm)

14

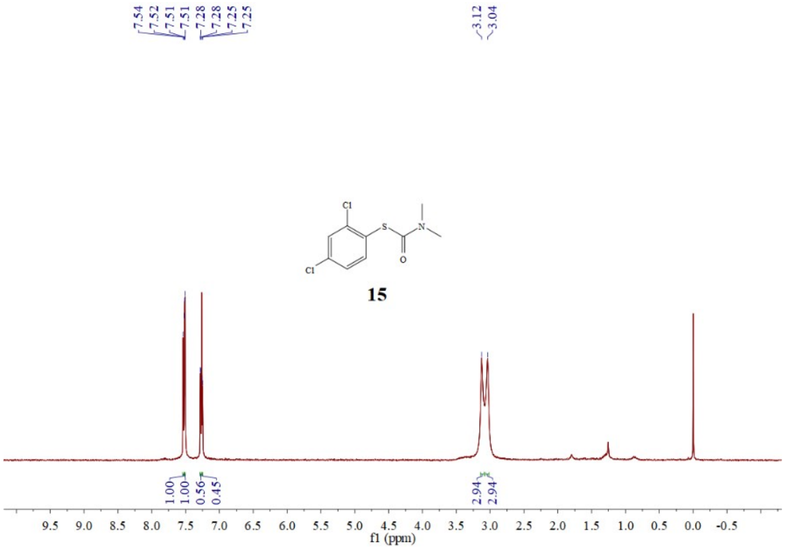
7.54
7.52
7.51
7.51
7.28
7.28
7.25
7.25
3.12
3.04
Cl
S
N
O
Cl
1.00
1.00
0.56
0.45
2.94
2.94
9.5 9.0 8.5 8.0 7.5 7.0 6.5 6.0 5.5 5.0 4.5 4.0 3.5 3.0 2.5 2.0 1.5 1.0 0.5 0.0 -0.5
f1 (ppm)

15

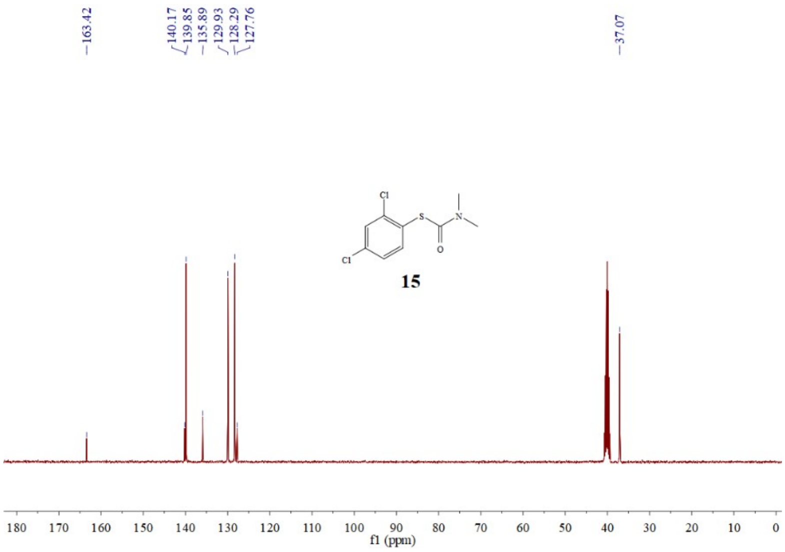
163.42
140.17
139.85
135.89
129.93
128.29
127.76
37.07
Cl
S
N
O
Cl
15
180 170 160 150 140 130 120 110 100 90 80 70 60 50 40 30 20 10 0
f1 (ppm)

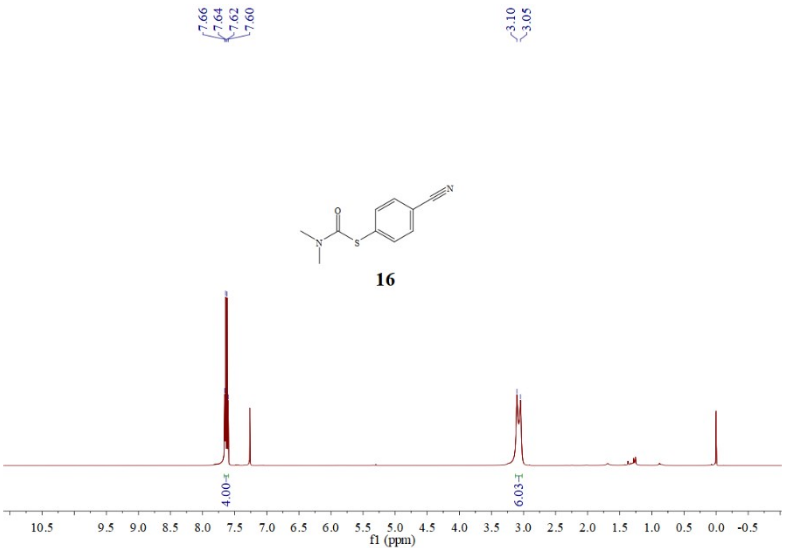
7.66
7.64
7.62
7.60
3.10
3.05
O
N
S
N
16
4.00
6.03
10.5 9.5 9.0 8.5 8.0 7.5 7.0 6.5 6.0 5.5 5.0 4.5 4.0 3.5 3.0 2.5 2.0 1.5 1.0 0.5 0.0 -0.5
f1 (ppm)

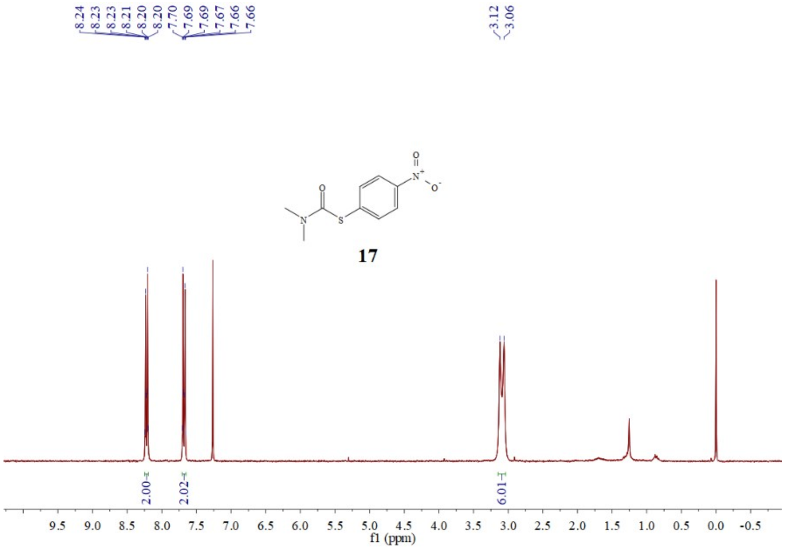
8.24
8.23
8.23
8.21
8.20
8.20
7.70
7.69
7.69
7.67
7.66
7.66
3.12
3.06
O
N
O
N
S
17
2.00
2.02
6.01
9.5 9.0 8.5 8.0 7.5 7.0 6.5 6.0 5.5 5.0 4.5 4.0 3.5 3.0 2.5 2.0 1.5 1.0 0.5 0.0 -0.5
f1 (ppm)

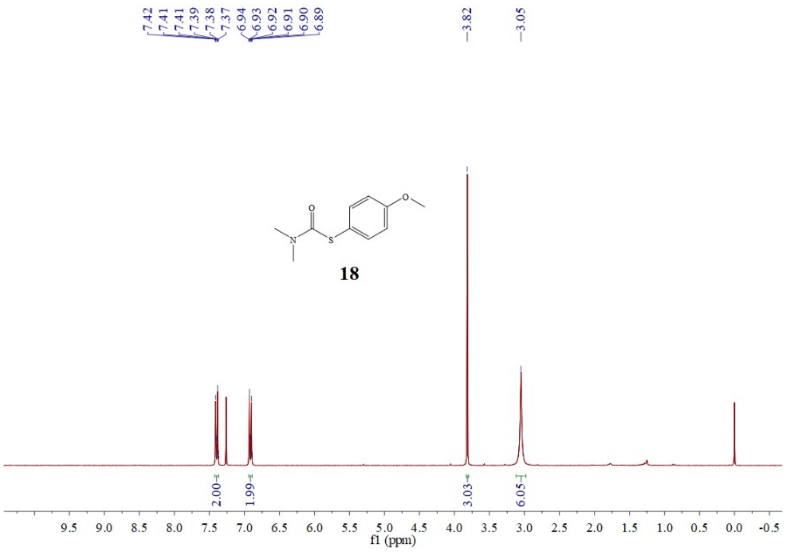
7.42
7.41
7.41
7.39
7.38
7.37
6.94
6.93
6.92
6.91
6.90
6.89
3.82
3.05
2.00
1.99
3.03
6.05
9.5 9.0 8.5 8.0 7.5 7.0 6.5 6.0 5.5 5.0 4.5 4.0 3.5 3.0 2.5 2.0 1.5 1.0 0.5 0.0 -0.5
f1 (ppm)

18

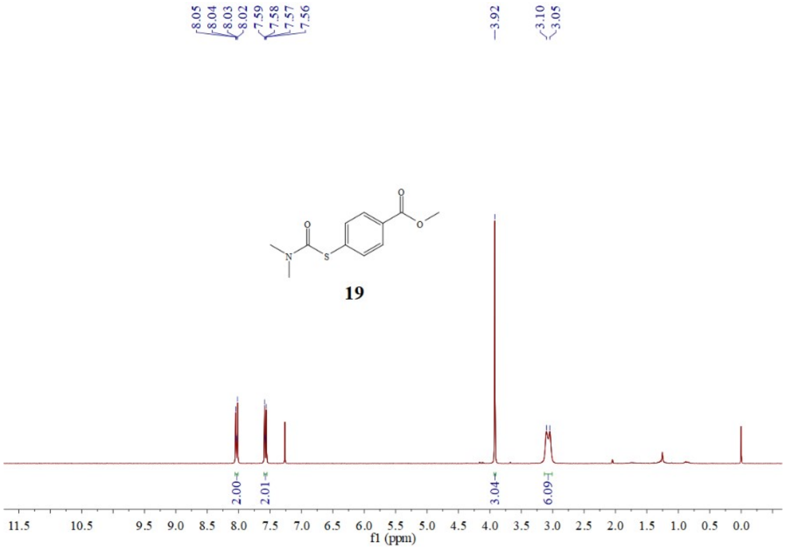
8.05
8.04
8.03
8.02
7.59
7.58
7.57
7.56
3.92
3.10
3.05
2.00
2.01
3.04
6.09
11.5 10.5 9.5 9.0 8.5 8.0 7.5 7.0 6.5 6.0 5.5 5.0 4.5 4.0 3.5 3.0 2.5 2.0 1.5 1.0 0.5 0.0
f1 (ppm)

19

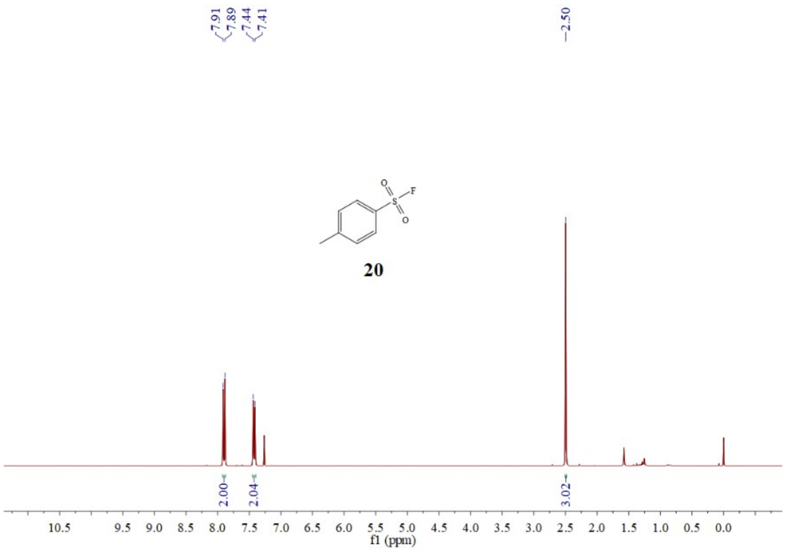
7.91
7.89
7.44
7.41
2.50
2.00
2.04
3.02
10.5 9.5 9.0 8.5 8.0 7.5 7.0 6.5 6.0 5.5 5.0 4.5 4.0 3.5 3.0 2.5 2.0 1.5 1.0 0.5 0.0
f1 (ppm)

20

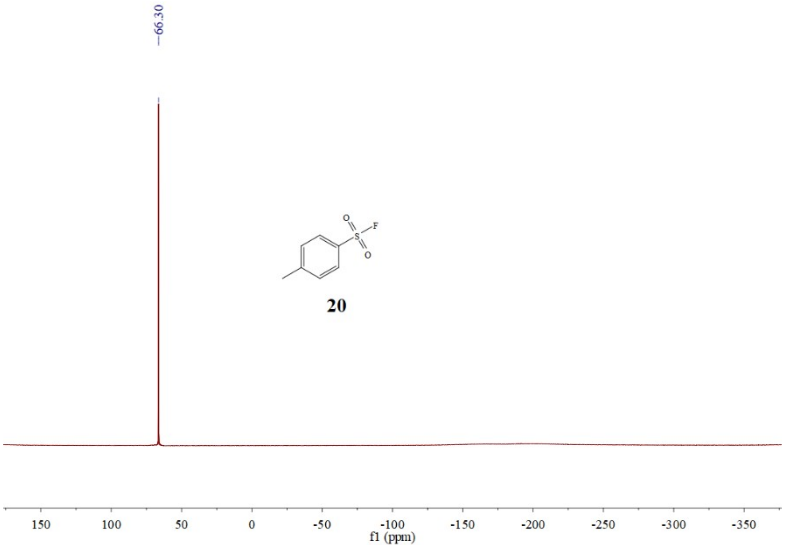

66.30
O
S
F
O
20
150 100 50 0 -50 -100 -150 -200 -250 -300 -350
f1 (ppm)

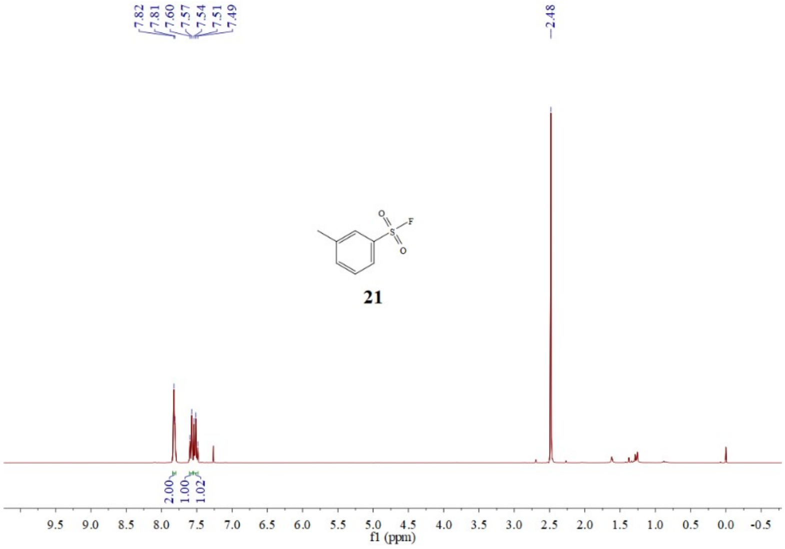

7.82
7.81
7.60
7.57
7.54
7.51
7.49
2.48
O
S
F
O
21
2.00
1.00
1.02
9.5 9.0 8.5 8.0 7.5 7.0 6.5 6.0 5.5 5.0 4.5 4.0 3.5 3.0 2.5 2.0 1.5 1.0 0.5 0.0 -0.5
f1 (ppm)

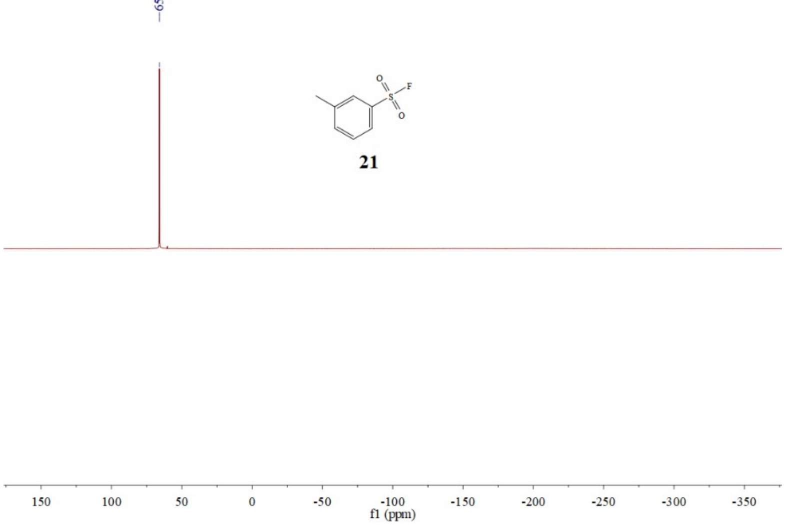

65.83
O
S
F
O
21
150 100 50 0 -50 -100 -150 -200 -250 -300 -350
f1 (ppm)

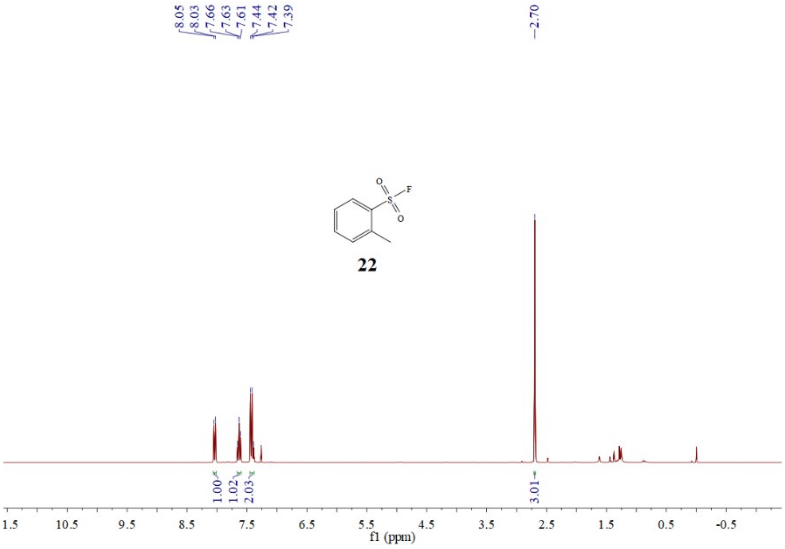
8.05
8.03
7.66
7.63
7.61
7.44
7.42
7.39
2.70
22
1.00
1.02
2.03
3.01
1.5 10.5 9.5 8.5 7.5 6.5 5.5 4.5 3.5 2.5 1.5 0.5 -0.5
f1 (ppm)

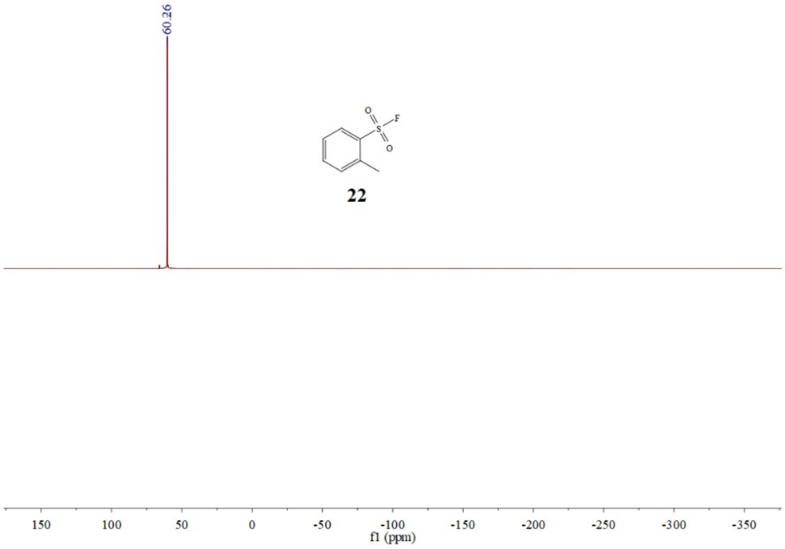
60.26
22
150 100 50 0 -50 -100 -150 -200 -250 -300 -350
f1 (ppm)

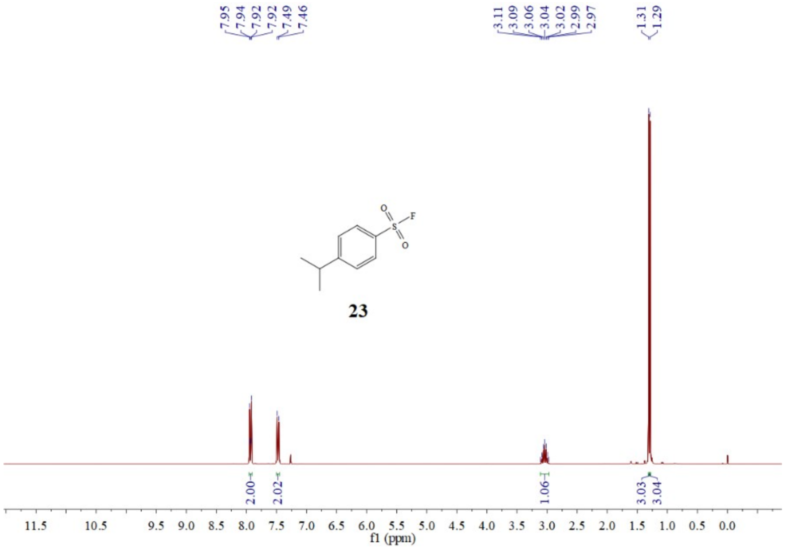
7.95
7.94
7.92
7.49
7.46
3.11
3.09
3.06
3.04
3.02
2.99
2.97
1.31
1.29
23
2.00
2.02
1.06
3.03
3.04
11.5 10.5 9.5 9.0 8.5 8.0 7.5 7.0 6.5 6.0 5.5 5.0 4.5 4.0 3.5 3.0 2.5 2.0 1.5 1.0 0.5 0.0
f1 (ppm)

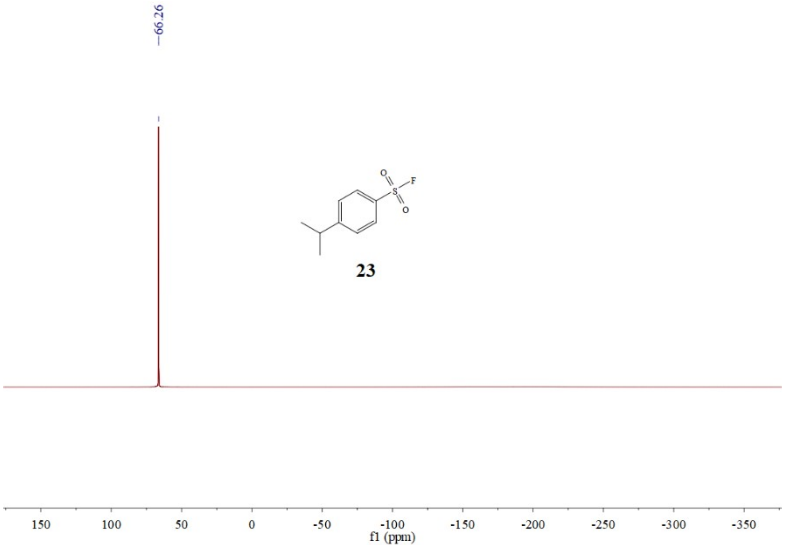

66.26
F
23
150 100 50 0 -50 -100 -150 -200 -250 -300 -350
f1 (ppm)

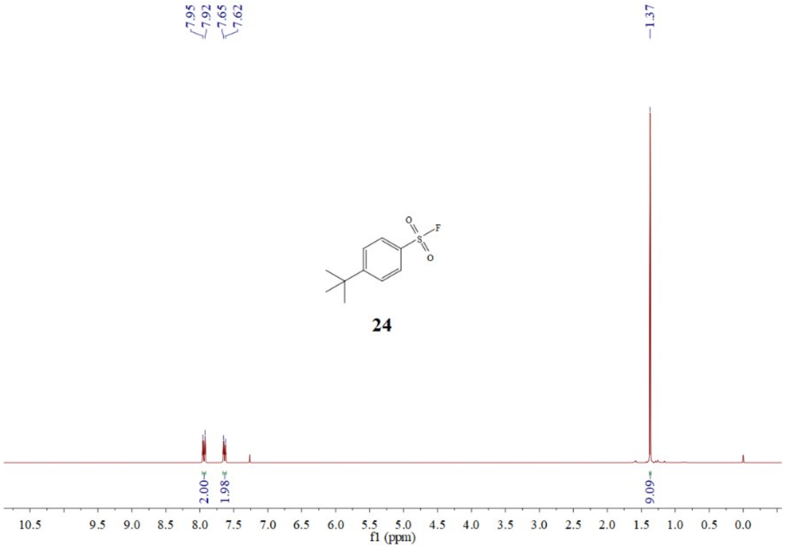

7.95
7.92
7.65
7.62
1.37
F
24
2.00
1.98
9.09
10.5 9.5 9.0 8.5 8.0 7.5 7.0 6.5 6.0 5.5 5.0 4.5 4.0 3.5 3.0 2.5 2.0 1.5 1.0 0.5 0.0
f1 (ppm)

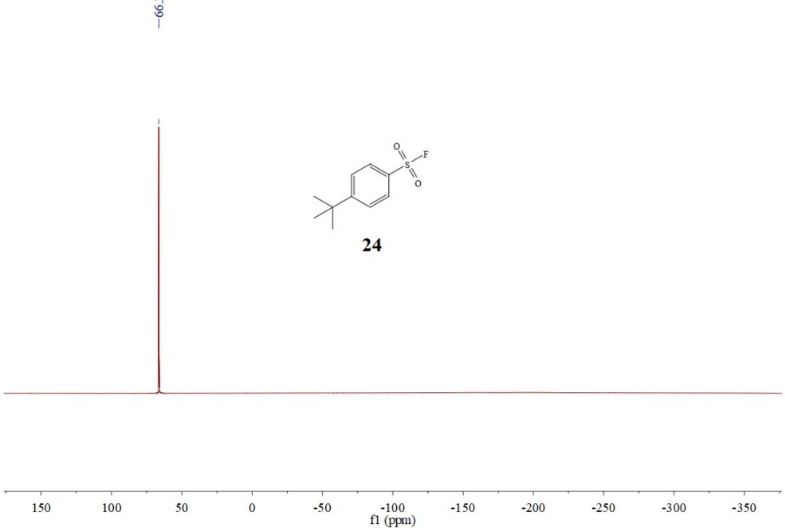

66.23
F
24
150 100 50 0 -50 -100 -150 -200 -250 -300 -350
f1 (ppm)

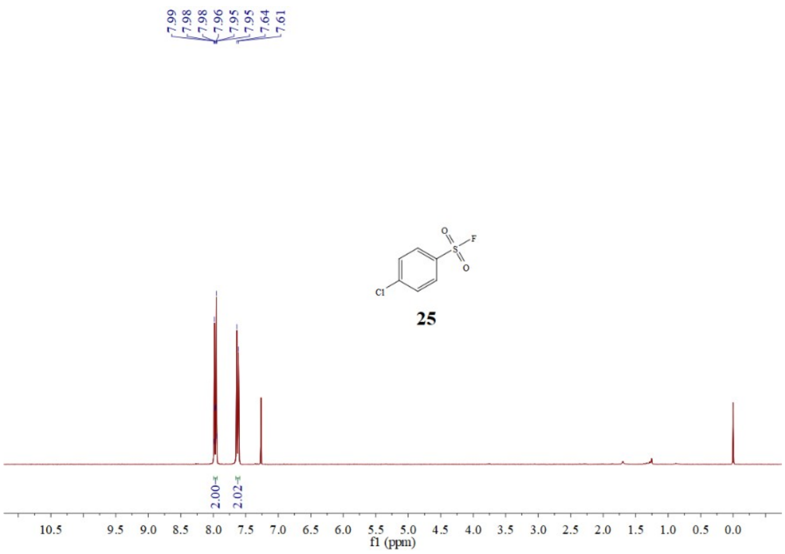
7.99
7.98
7.98
7.96
7.95
7.95
7.64
7.61
2.00
2.02
Cl
S
O
O
F
25
10.5 9.5 9.0 8.5 8.0 7.5 7.0 6.5 6.0 5.5 5.0 4.5 4.0 3.5 3.0 2.5 2.0 1.5 1.0 0.5 0.0
f1 (ppm)

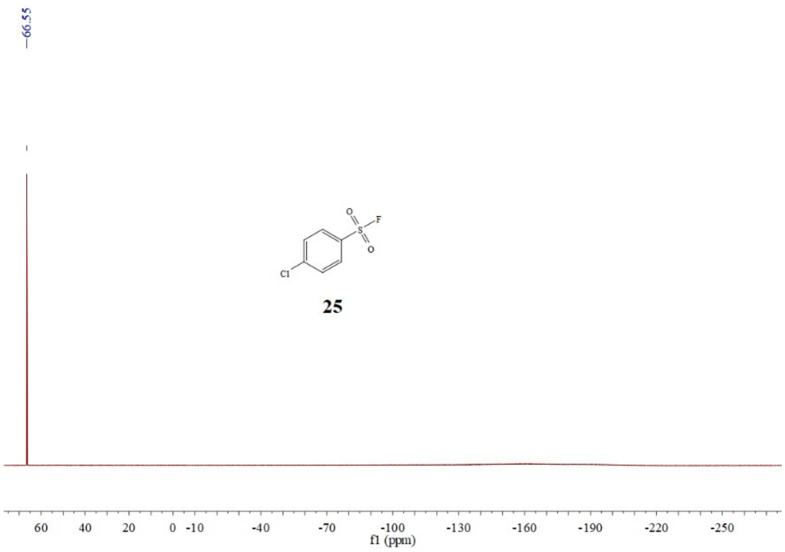
66.55
Cl
S
O
O
F
25
60 40 20 0 -10 -40 -70 -100 -130 -160 -190 -220 -250
f1 (ppm)

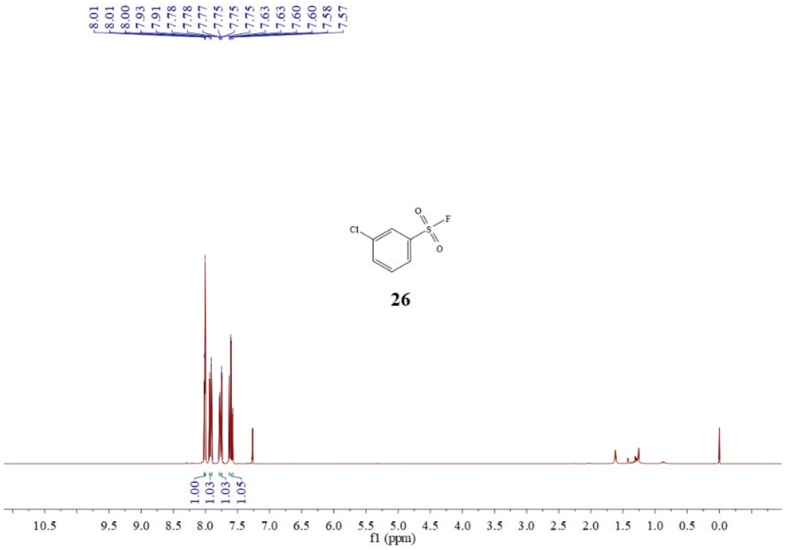
8.01
8.01
8.00
7.93
7.91
7.78
7.78
7.77
7.75
7.75
7.75
7.63
7.63
7.60
7.60
7.58
7.57
1.00
1.03
1.03
1.05
Cl
S
O
O
F
26
10.5 9.5 9.0 8.5 8.0 7.5 7.0 6.5 6.0 5.5 5.0 4.5 4.0 3.5 3.0 2.5 2.0 1.5 1.0 0.5 0.0
f1 (ppm)

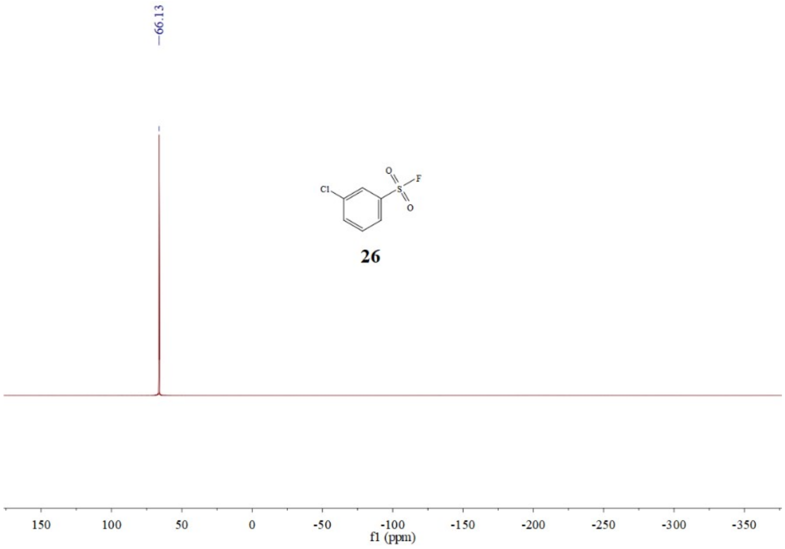

—66.13
Cl
O
S
F
O
26
150 100 50 0 -50 -100 -150 -200 -250 -300 -350
f1 (ppm)

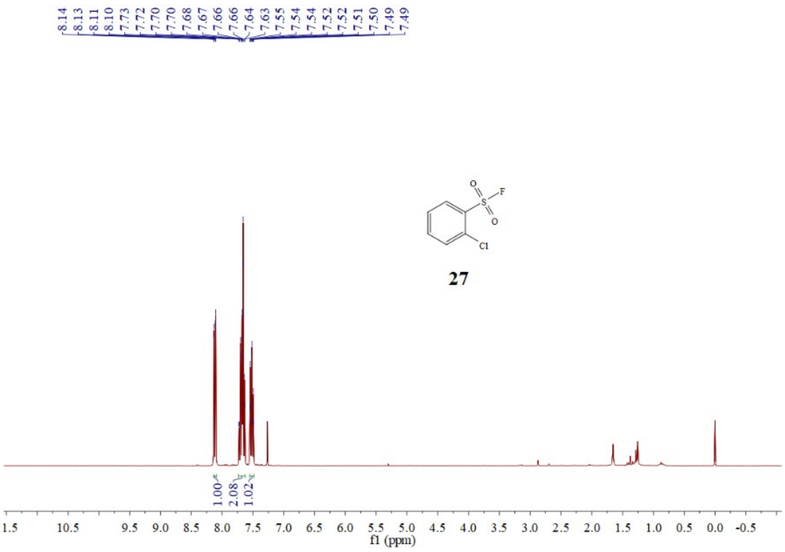

8.14 8.13 8.11 8.10 7.73 7.72 7.70 7.70 7.68 7.67 7.66 7.66 7.64 7.63 7.55 7.54 7.54 7.52 7.52 7.51 7.50 7.49 7.49
O
S
F
O
Cl
27
1.00 2.08 1.02
1.5 10.5 9.5 9.0 8.5 8.0 7.5 7.0 6.5 6.0 5.5 5.0 4.5 4.0 3.5 3.0 2.5 2.0 1.5 1.0 0.5 0.0 -0.5
f1 (ppm)

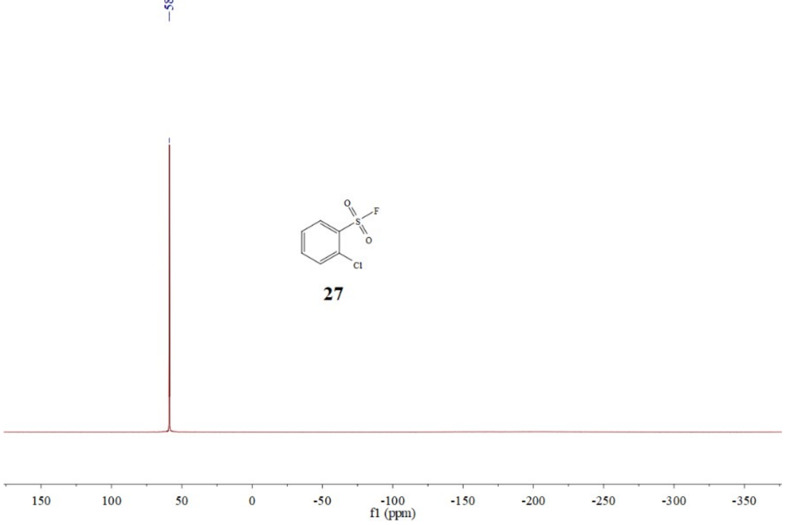

—58.88
O
S
F
O
Cl
27
150 100 50 0 -50 -100 -150 -200 -250 -300 -350
f1 (ppm)

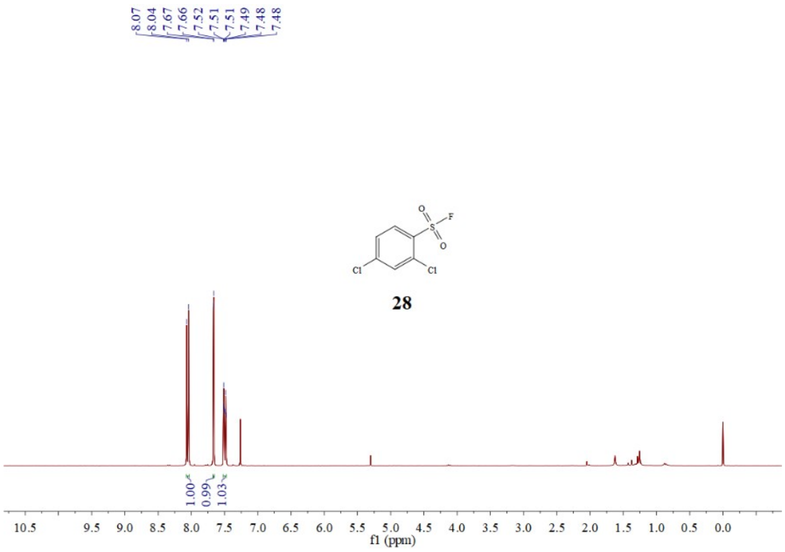
8.07
8.04
7.67
7.66
7.52
7.51
7.51
7.49
7.48
7.48
Cl
Cl
S
O
O
F
28
1.00
0.99
1.03
10.5 9.5 9.0 8.5 8.0 7.5 7.0 6.5 6.0 5.5 5.0 4.5 4.0 3.5 3.0 2.5 2.0 1.5 1.0 0.5 0.0
f1 (ppm)

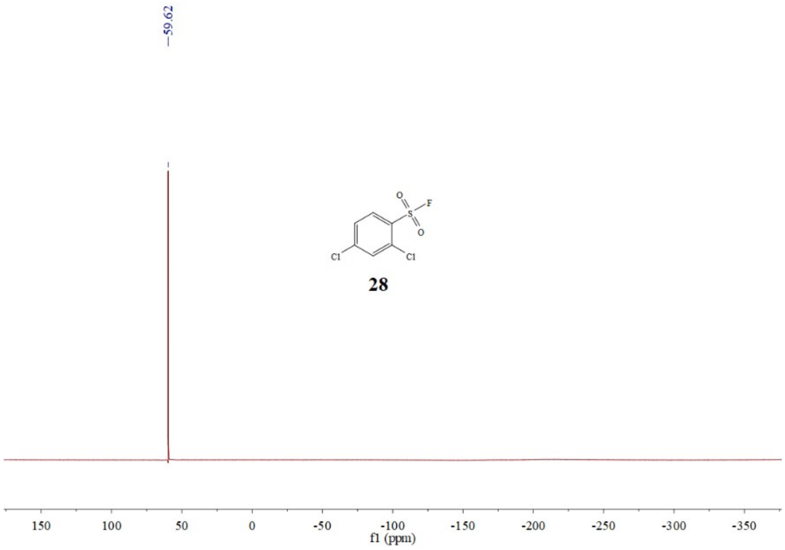
59.62
Cl
Cl
S
O
O
F
28
150 100 50 0 -50 -100 -150 -200 -250 -300 -350
f1 (ppm)

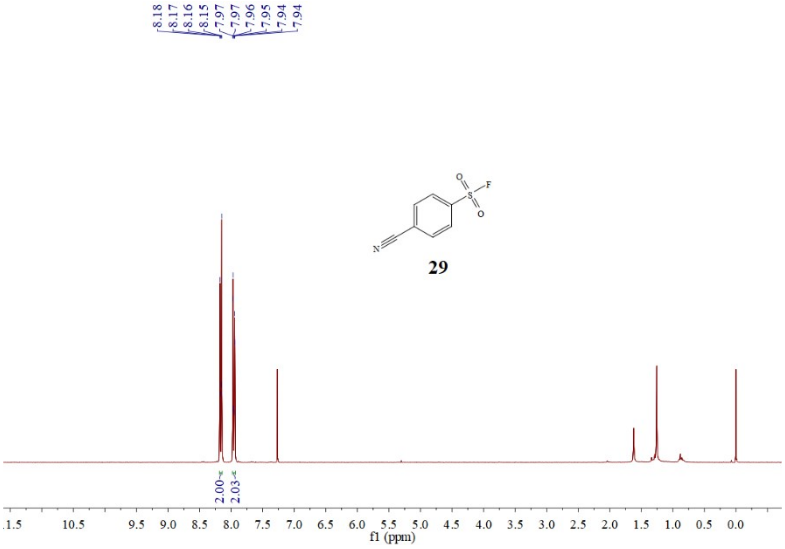
8.18
8.17
8.16
8.15
7.97
7.97
7.96
7.95
7.94
7.94
N
S
O
O
F
29
2.00
2.03
.5 10.5 9.5 9.0 8.5 8.0 7.5 7.0 6.5 6.0 5.5 5.0 4.5 4.0 3.5 3.0 2.5 2.0 1.5 1.0 0.5 0.0
f1 (ppm)

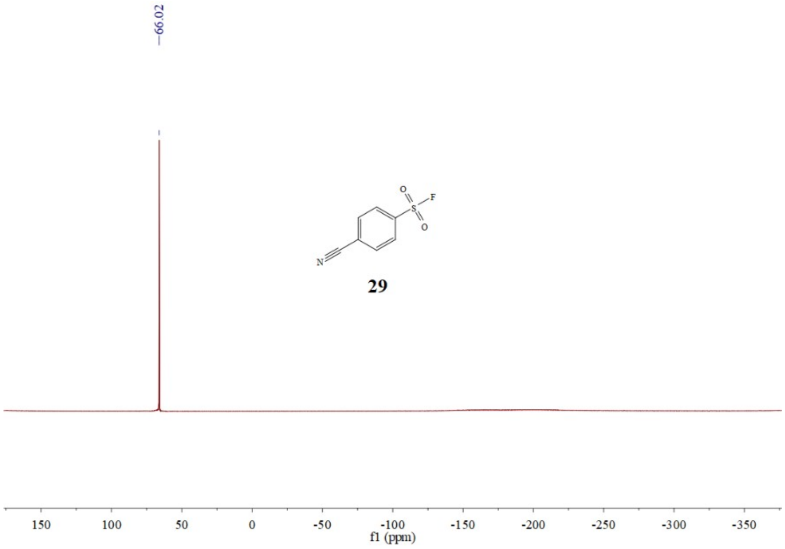

66.02
29
150 100 50 0 -50 -100 -150 -200 -250 -300 -350
f1 (ppm)

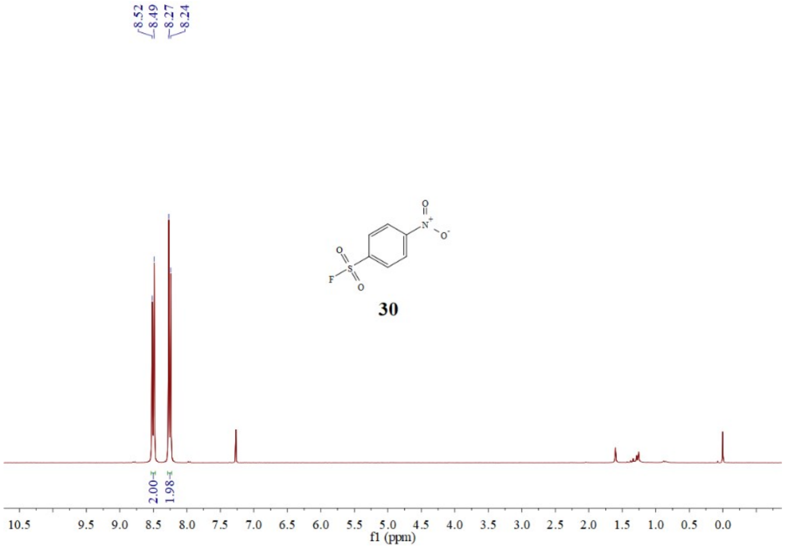

8.52
8.49
8.27
8.24
30
2.00
1.98
10.5 9.5 9.0 8.5 8.0 7.5 7.0 6.5 6.0 5.5 5.0 4.5 4.0 3.5 3.0 2.5 2.0 1.5 1.0 0.5 0.0
f1 (ppm)

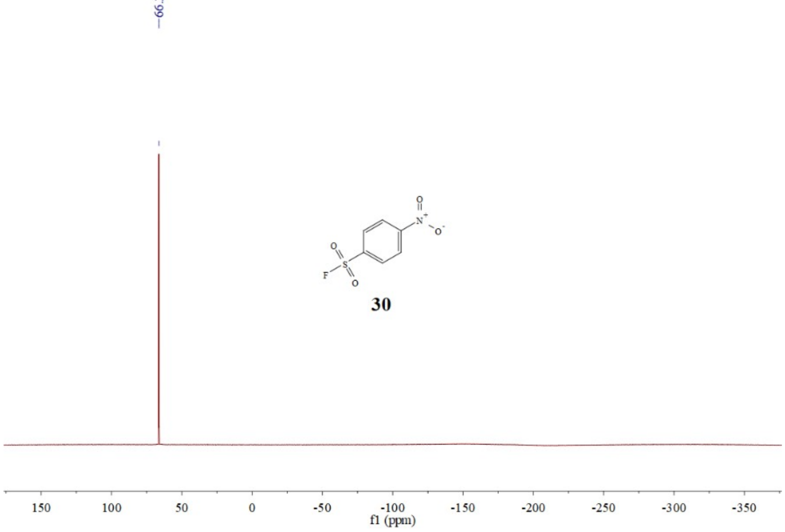

66.25
30
150 100 50 0 -50 -100 -150 -200 -250 -300 -350
f1 (ppm)

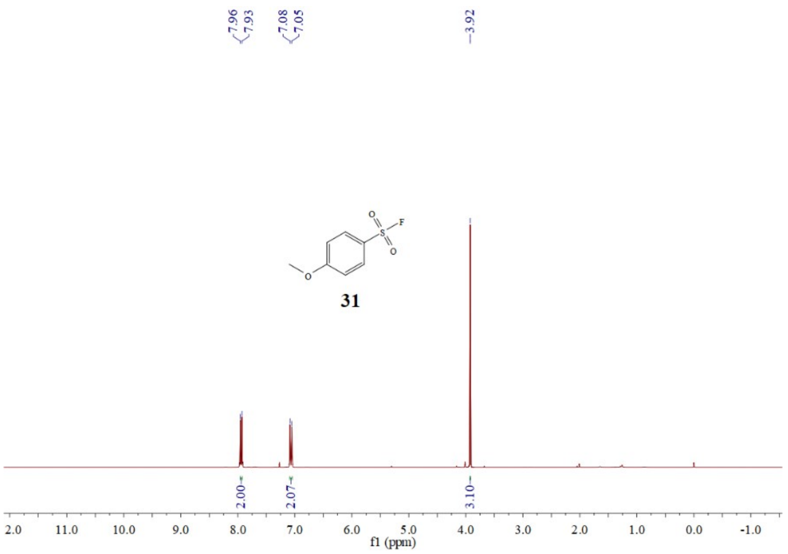
7.96
7.93
7.08
7.05
3.92
31
2.00
2.07
3.10
2.0 11.0 10.0 9.0 8.0 7.0 6.0 5.0 4.0 3.0 2.0 1.0 0.0 -1.0
f1 (ppm)

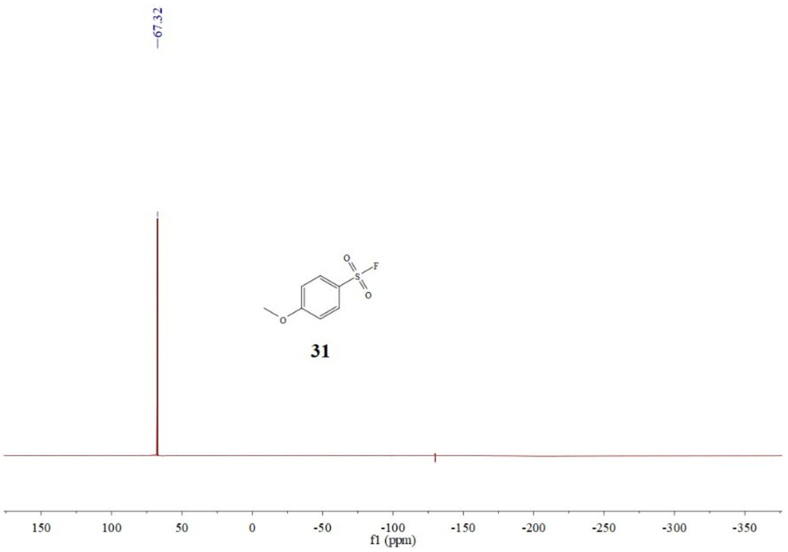
-67.32
31
150 100 50 0 -50 -100 -150 -200 -250 -300 -350
f1 (ppm)

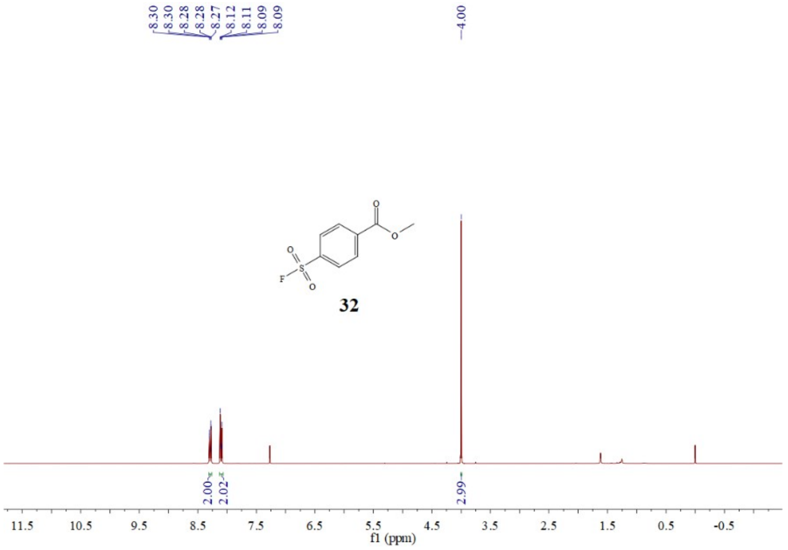
8.30
8.30
8.28
8.27
8.12
8.11
8.09
8.09
4.00
32
2.00
2.02
2.99
11.5 10.5 9.5 8.5 7.5 6.5 5.5 4.5 3.5 2.5 1.5 0.5 -0.5
f1 (ppm)

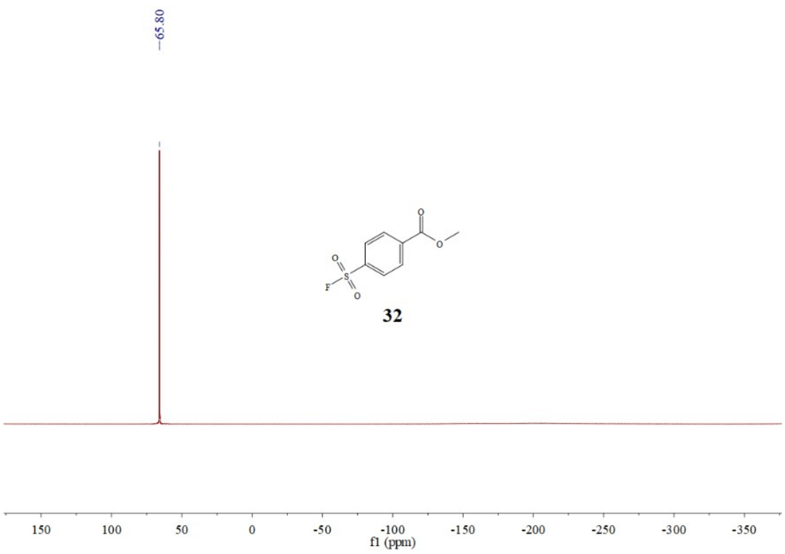

65.80
150 100 50 0 -50 -100 -150 -200 -250 -300 -350
f1 (ppm)

32

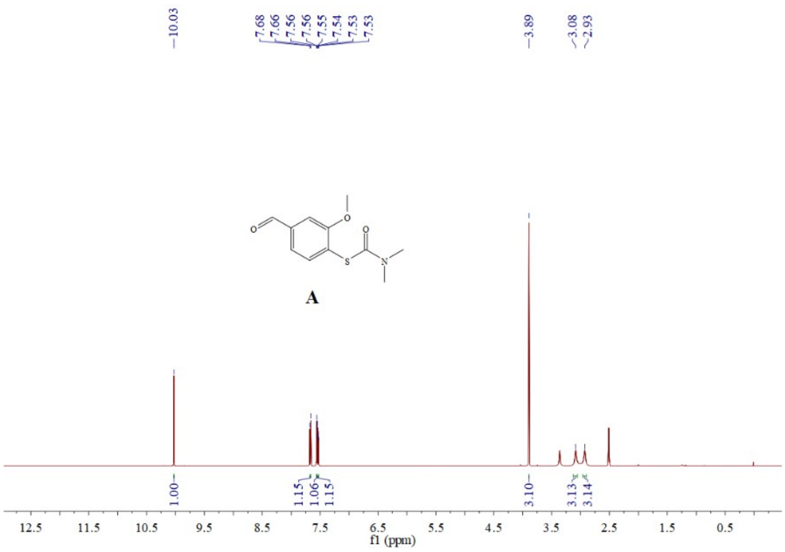

10.03
7.68 7.66 7.56 7.56 7.55 7.54 7.53 7.53
3.89
3.08 2.93
1.00 1.15 1.06 1.15 3.10 3.13 3.14
12.5 11.5 10.5 9.5 8.5 7.5 6.5 5.5 4.5 3.5 2.5 1.5 0.5
f1 (ppm)

A

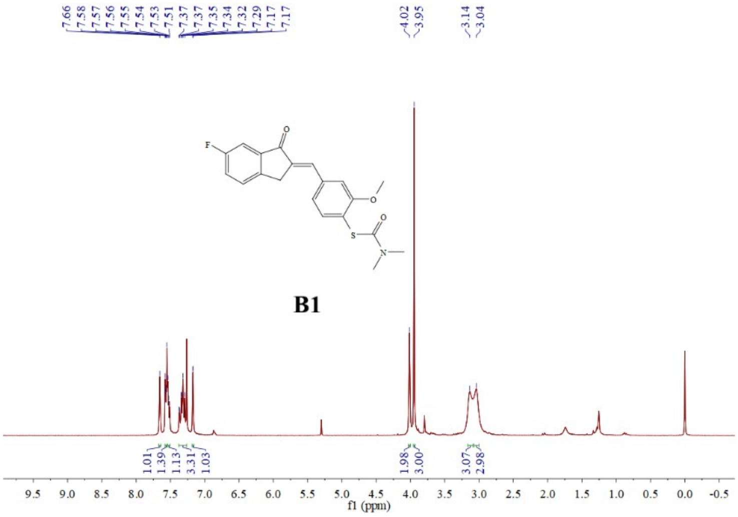

7.66 7.58 7.57 7.56 7.55 7.54 7.53 7.51 7.37 7.37 7.35 7.34 7.32 7.29 7.17 7.17
4.02 3.95
3.14 3.04
1.01 1.39 1.13 3.31 1.03 1.98 3.00 3.07 2.98
9.5 9.0 8.5 8.0 7.5 7.0 6.5 6.0 5.5 5.0 4.5 4.0 3.5 3.0 2.5 2.0 1.5 1.0 0.5 0.0 -0.5
f1 (ppm)

B1

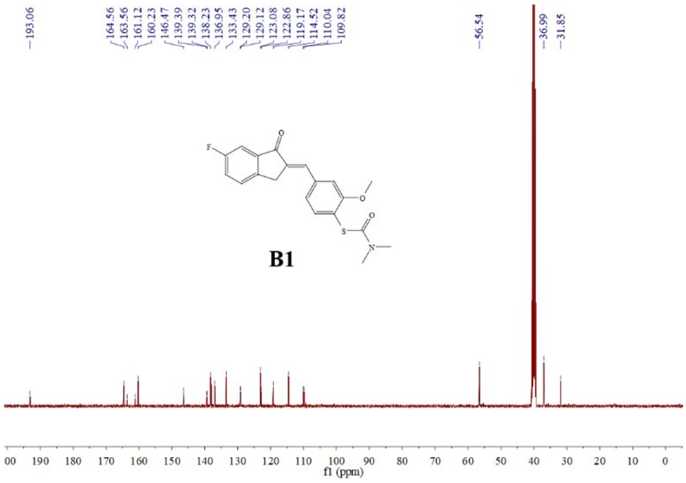
193.06
164.56
163.56
161.12
160.23
146.47
139.39
139.32
138.23
136.95
133.43
129.20
129.12
123.08
122.86
119.17
114.52
110.04
109.82
56.54
36.99
31.85
B1
f1 (ppm)

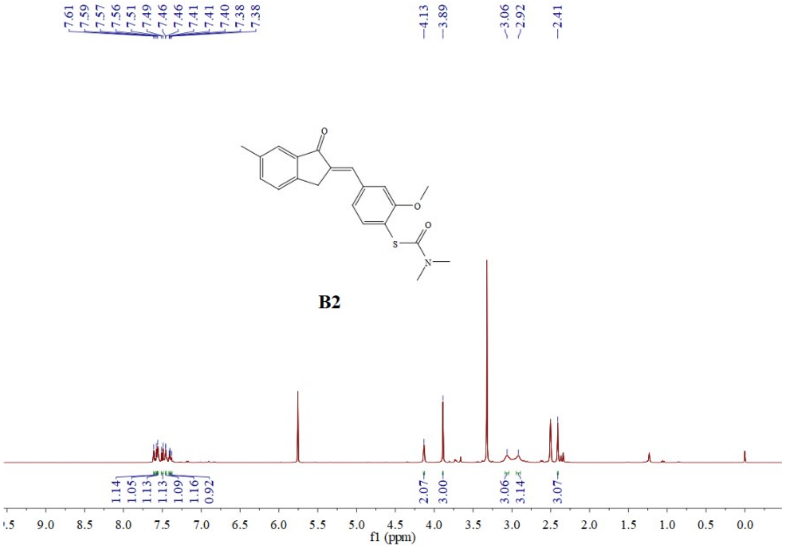
7.61
7.59
7.57
7.56
7.51
7.49
7.46
7.46
7.41
7.41
7.40
7.38
7.38
4.13
3.89
3.06
2.92
2.41
B2
1.14
1.05
1.13
1.13
1.09
1.16
0.92
2.07
3.00
3.06
3.14
3.07
f1 (ppm)

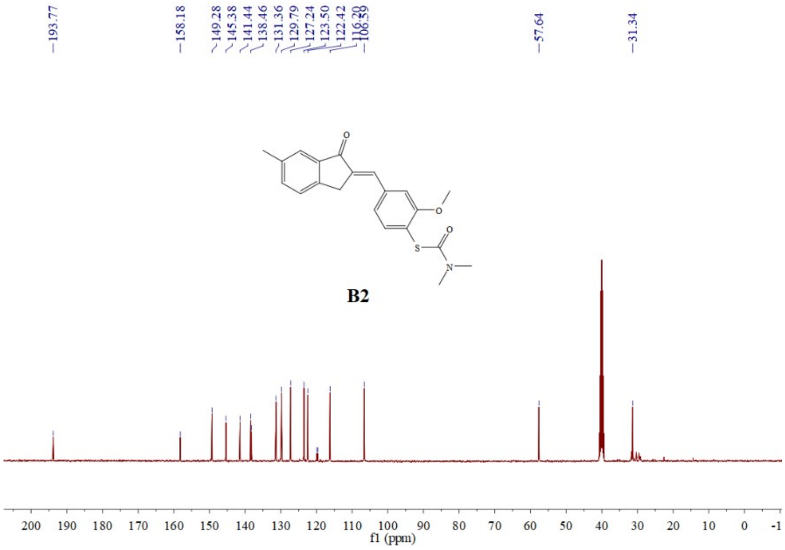
193.77
158.18
149.28
145.38
141.44
138.46
131.36
129.79
127.24
123.50
122.42
57.64
31.34
B2
f1 (ppm)

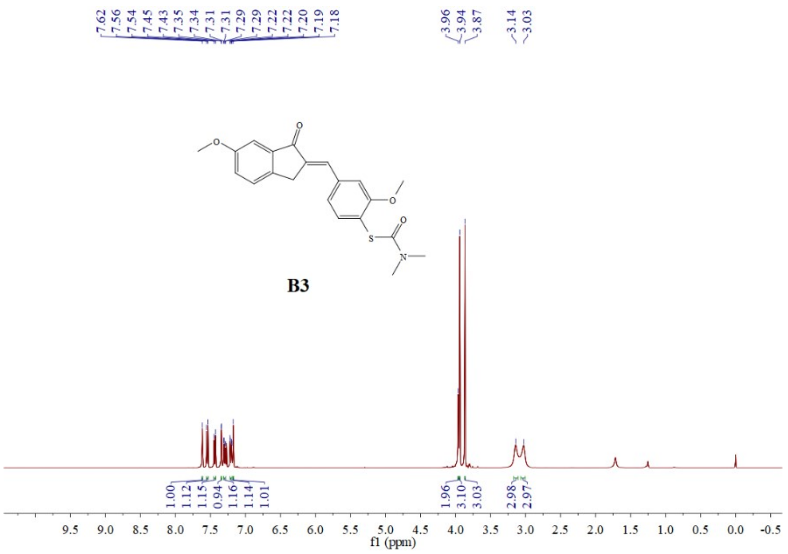
7.62
7.56
7.54
7.45
7.43
7.35
7.34
7.31
7.31
7.29
7.22
7.22
7.20
7.19
7.18
3.96
3.94
3.87
3.14
3.03
B3
1.00
1.12
1.15
0.94
1.16
1.14
1.01
1.96
3.10
3.03
2.98
2.97
9.5 9.0 8.5 8.0 7.5 7.0 6.5 6.0 5.5 5.0 4.5 4.0 3.5 3.0 2.5 2.0 1.5 1.0 0.5 0.0 -0.5
f1 (ppm)

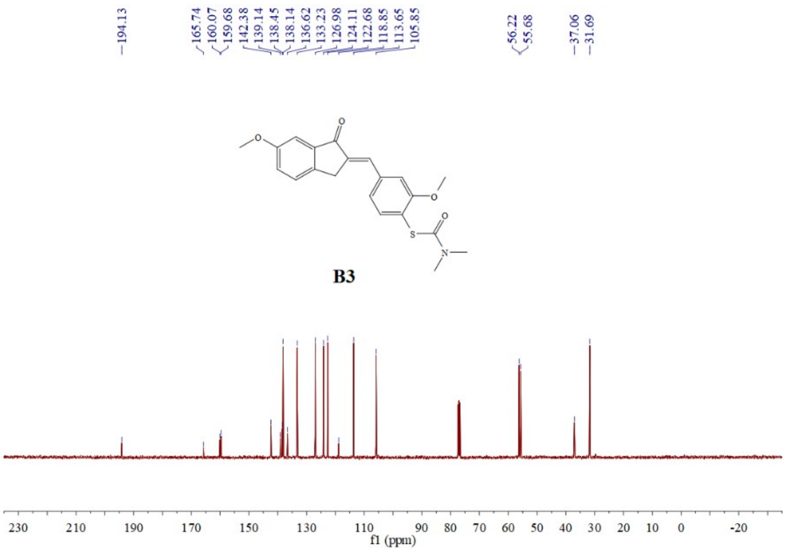
194.13
165.74
160.07
159.68
142.38
139.14
138.45
138.14
136.62
133.23
126.98
124.11
122.68
118.85
113.65
105.85
56.22
55.68
37.06
31.69
B3
230 210 190 170 150 130 110 90 80 70 60 50 40 30 20 10 0 -20
f1 (ppm)

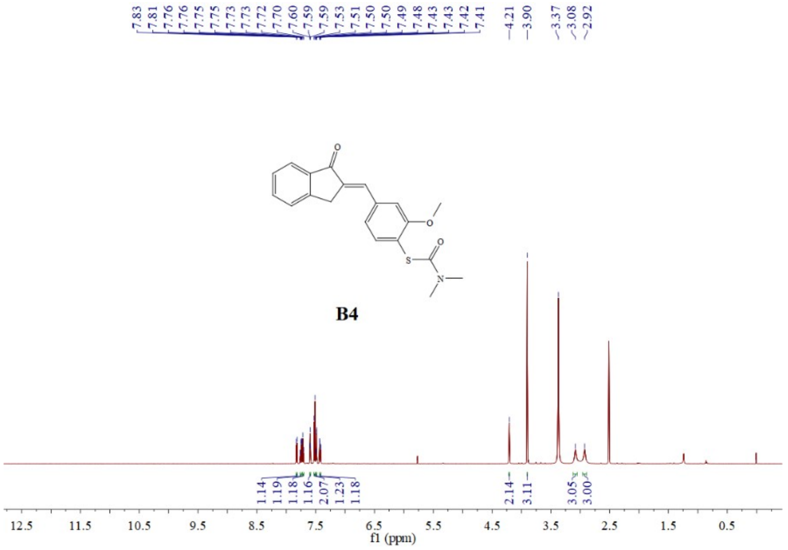
7.83
7.81
7.76
7.75
7.75
7.73
7.73
7.72
7.70
7.60
7.59
7.59
7.53
7.51
7.50
7.49
7.48
7.43
7.43
7.42
7.41
4.21
3.90
3.37
3.08
2.92
B4
1.14
1.19
1.18
1.16
2.07
1.23
1.18
2.14
3.11
3.05
3.00
12.5 11.5 10.5 9.5 8.5 7.5 6.5 5.5 4.5 3.5 2.5 1.5 0.5
f1 (ppm)

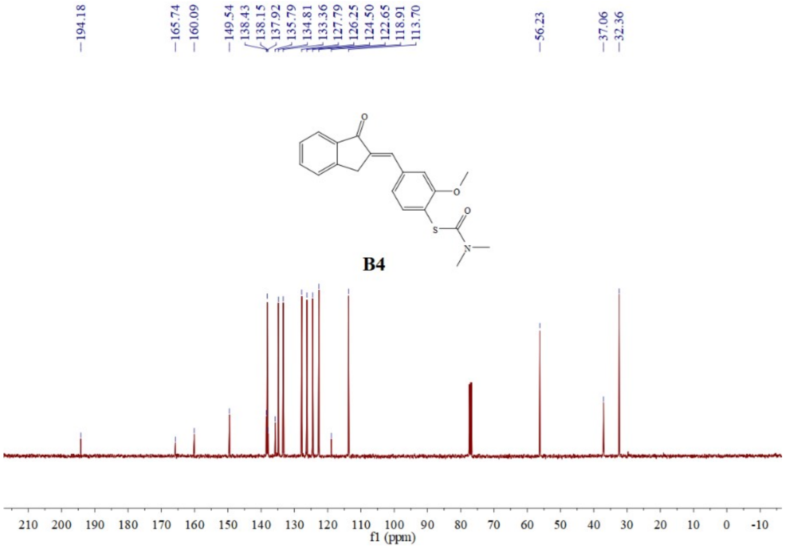
194.18
165.74
160.09
149.54
138.43
138.15
137.92
135.79
134.81
133.36
127.79
126.25
124.50
122.65
118.91
113.70
56.23
37.06
32.36
B4
210 200 190 180 170 160 150 140 130 120 110 100 90 80 70 60 50 40 30 20 10 0 -10
f1 (ppm)

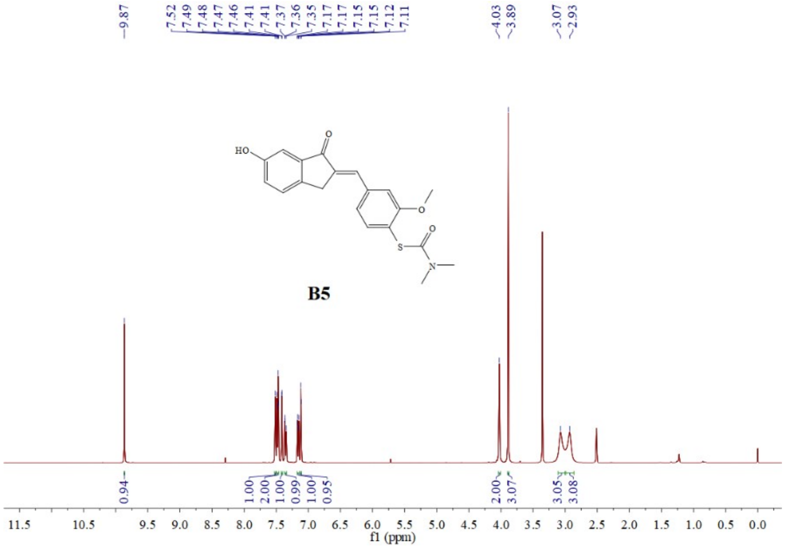
9.87
7.52
7.49
7.48
7.47
7.46
7.41
7.37
7.36
7.35
7.17
7.17
7.15
7.12
7.11
4.03
3.89
3.07
2.93
B5
0.94
1.00
2.00
1.00
0.99
1.00
0.95
2.00
3.07
3.05
3.08
11.5 10.5 9.5 9.0 8.5 8.0 7.5 7.0 6.5 6.0 5.5 5.0 4.5 4.0 3.5 3.0 2.5 2.0 1.5 1.0 0.5 0.0
f1 (ppm)

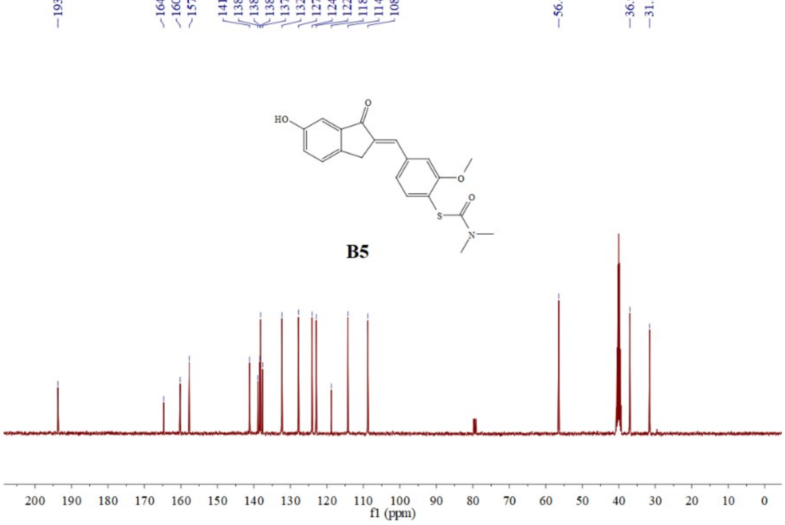
193.70
164.69
160.21
157.74
141.18
138.88
138.39
138.18
137.59
132.30
127.79
124.06
122.91
118.79
114.23
108.76
56.46
36.98
31.58
B5
200 190 180 170 160 150 140 130 120 110 100 90 80 70 60 50 40 30 20 10 0
f1 (ppm)

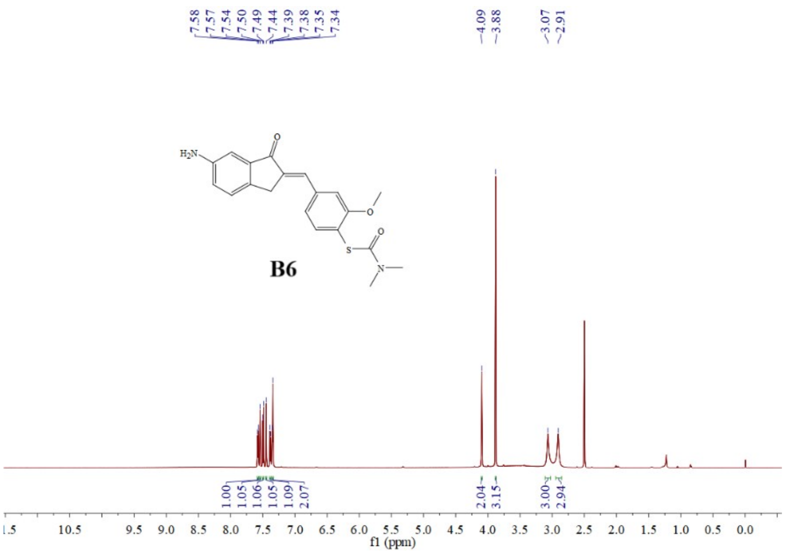

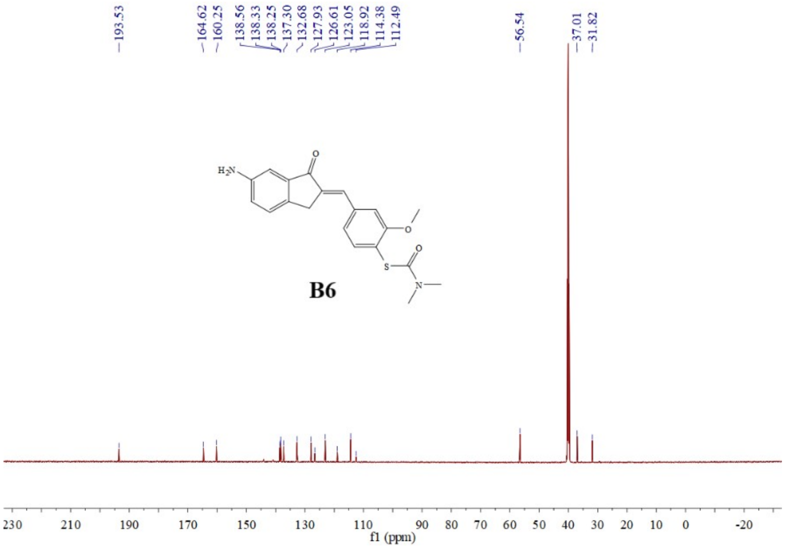

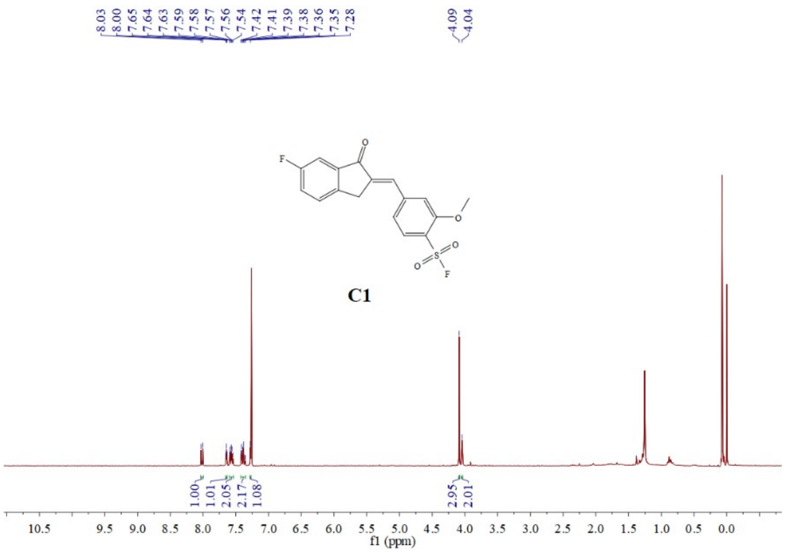

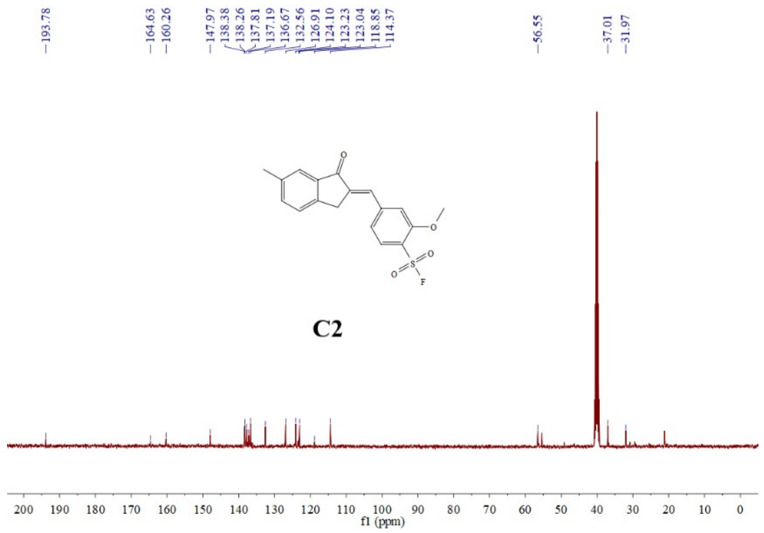
193.78
164.63
160.26
147.97
138.38
138.26
137.81
137.19
136.67
132.56
126.91
124.10
123.23
123.04
118.85
114.37
56.55
37.01
31.97
C2
200 190 180 170 160 150 140 130 120 110 100 90 80 70 60 50 40 30 20 10 0
f1 (ppm)

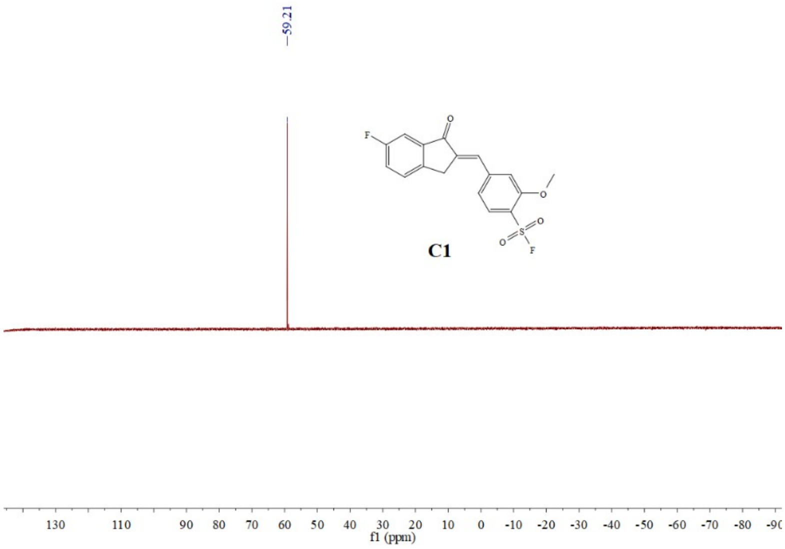
-59.21
F
O
C1
130 110 90 80 70 60 50 40 30 20 10 0 -10 -20 -30 -40 -50 -60 -70 -80
f1 (ppm)

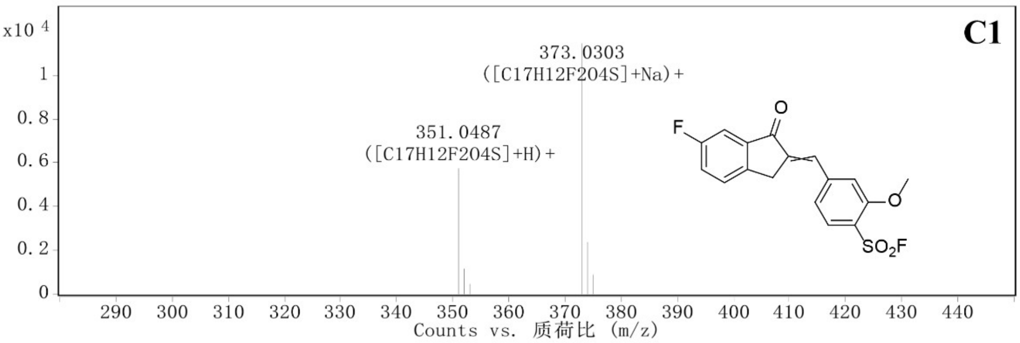
x10 4
C1
373.0303
([C17H12F2O4S]+Na)+
351.0487
([C17H12F2O4S]+H)+
F
O
SO2F
0 0.2 0.4 0.6 0.8 1
290 300 310 320 330 340 350 360 370 380 390 400 410 420 430 440
Counts vs. 质荷比 (m/z)

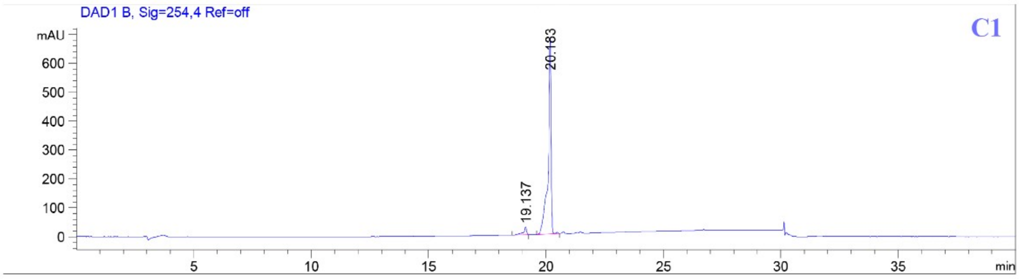

Signal 2: DAD1 B, Sig=254,4 Ref=off

Peak #	Retention time [min]	Type	Peak width [min]	Peak area [mAU*s]	Peak height [mAU]	Peak area %
1	19.137	BB	0.1250	245.13756	27.23357	3.9725
2	20.183	BV R	0.1214	5925.67725	678.72198	96.0275

Total amount : 6170.81480 705.95556

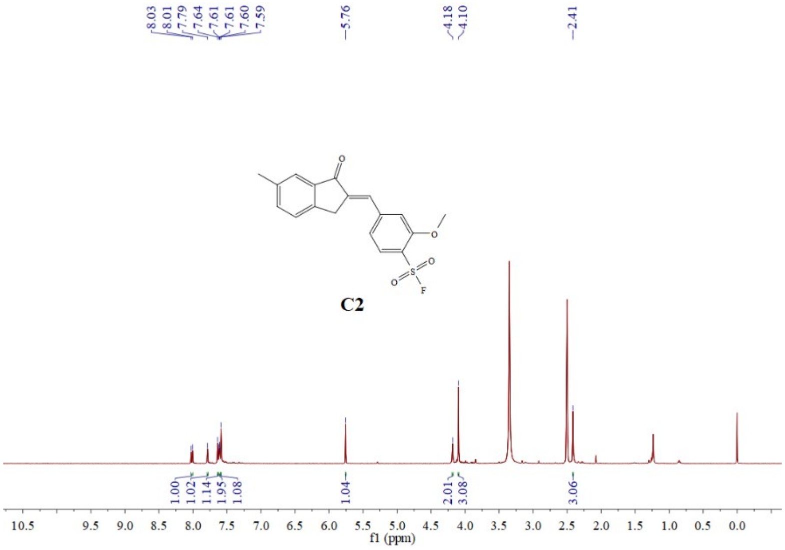

C2

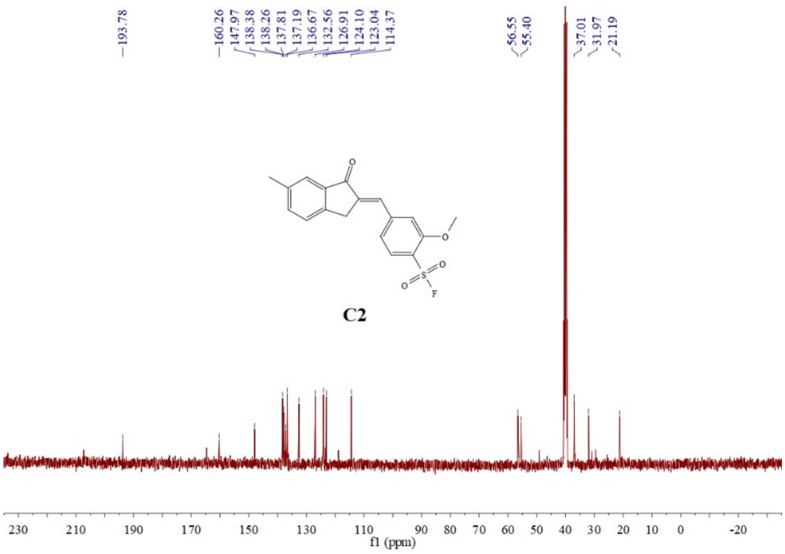
193.78
160.26
147.97
138.38
138.26
137.81
137.19
136.67
132.56
126.91
124.10
123.04
114.37
56.55
55.40
37.01
31.97
21.19
C2
230 210 190 170 150 130 110 90 80 70 60 50 40 30 20 10 0 -20
f1 (ppm)

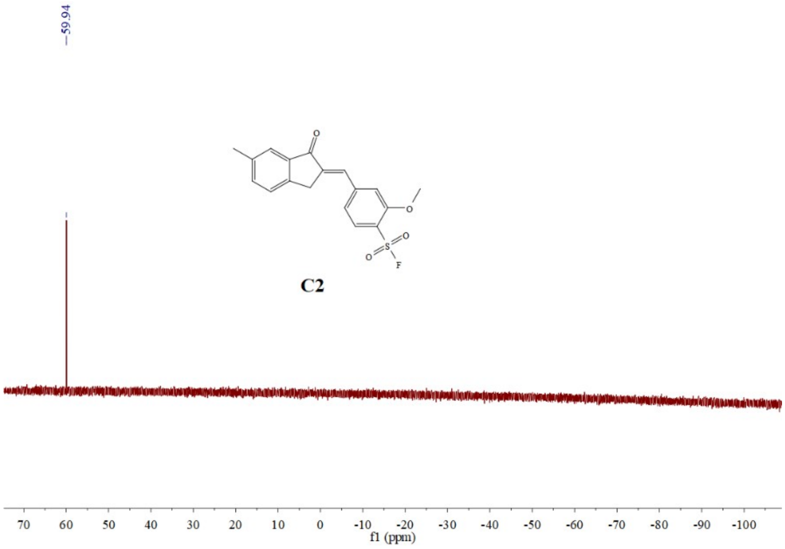
-59.94
C2
70 60 50 40 30 20 10 0 -10 -20 -30 -40 -50 -60 -70 -80 -90 -100
f1 (ppm)

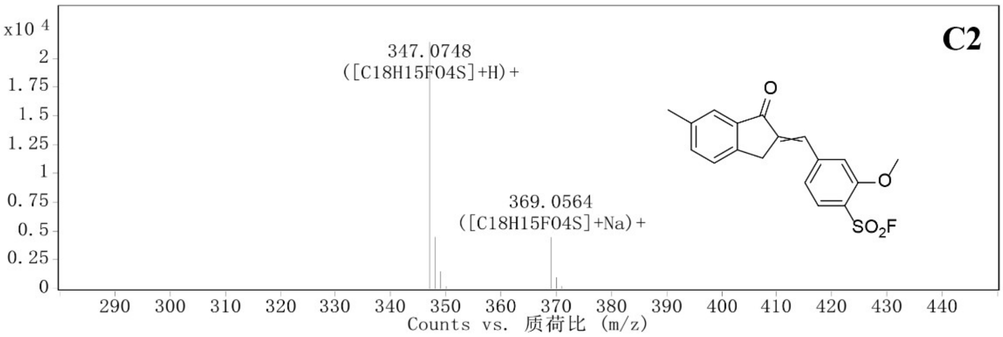
x10 4
C2
347.0748
([C18H15F04S]+H)+
369.0564
([C18H15F04S]+Na)+
SO2F
Counts vs. 质荷比 (m/z)

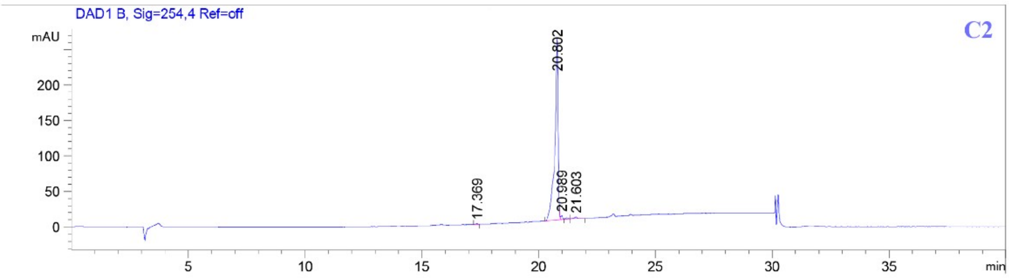

Signal 2: DAD1 B, Sig=254,4 Ref=off

Peak #	Retention time [min]	Type	Peak width [min]	Peak area [mAU*s]	Peak height [mAU]	Peak area %
1	17.369	BB	0.0852	5.93756	1.06267	0.2352
2	20.802	BV R	0.1313	2450.38867	254.77086	97.0786
3	20.989	VV E	0.0987	37.94141	5.62358	1.5031
4	21.603	BV R	0.1462	29.86185	2.89859	1.1831

Total amount : 2524.12949 264.35569

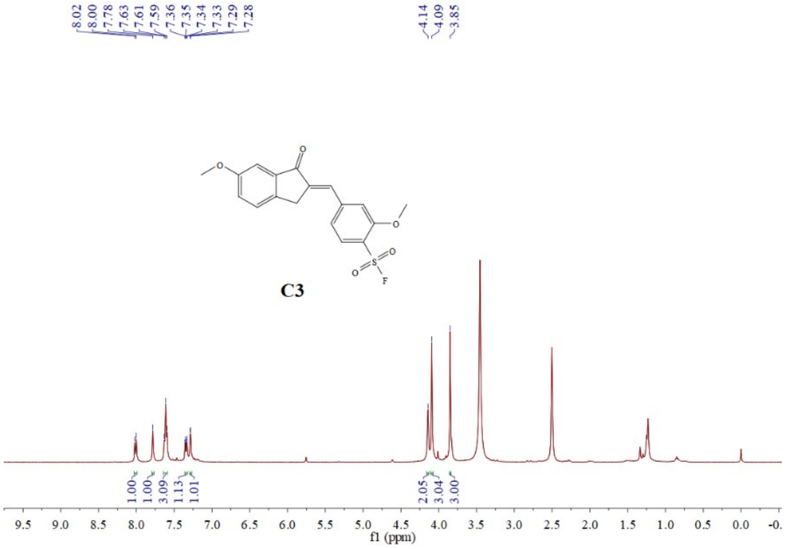

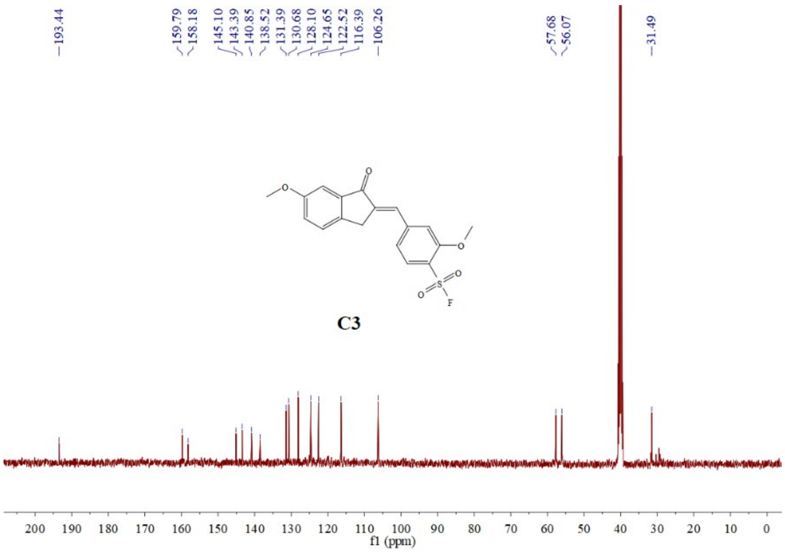

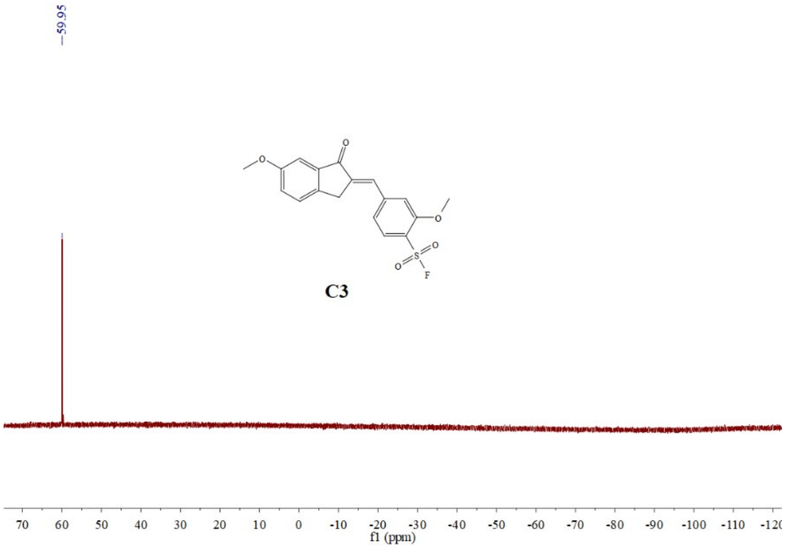

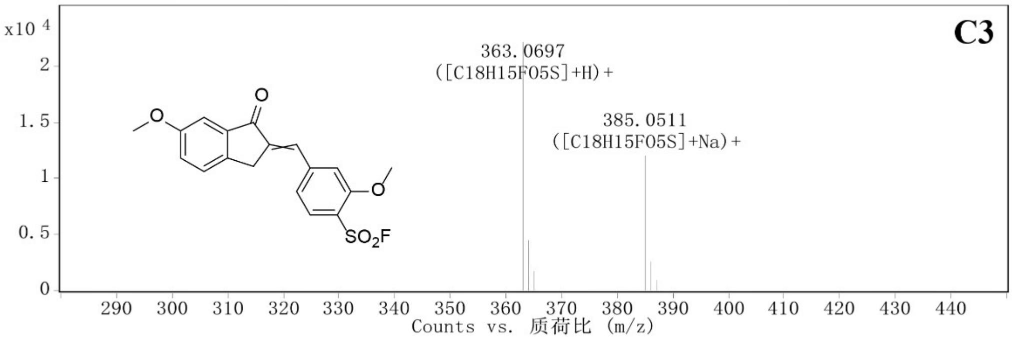

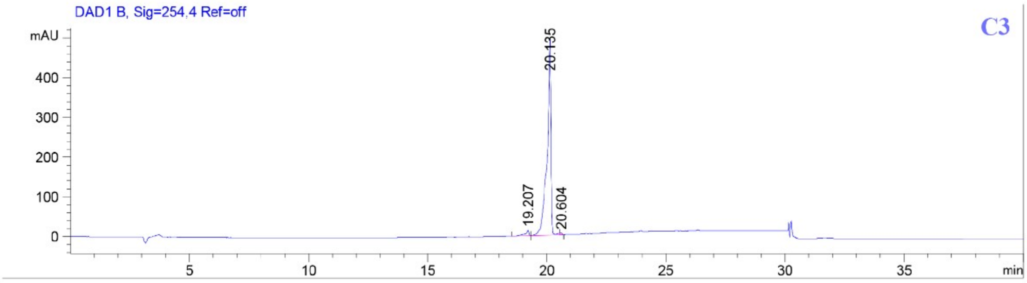

Signal 2: DAD1 B, Sig=254,4 Ref=off

Peak #	Retention time [min]	Type	Peak width [min]	Peak area [mAU*s]	Peak height [mAU]	Peak area %
1	19.207	BB	0.1455	140.46809	12.85713	2.5325
2	20.135	BV R	0.1454	5382.36816	497.23108	97.0406
3	20.604	VB E	0.0793	23.67564	4.36094	0.4269

Total amount : 5546.51190 514.44915

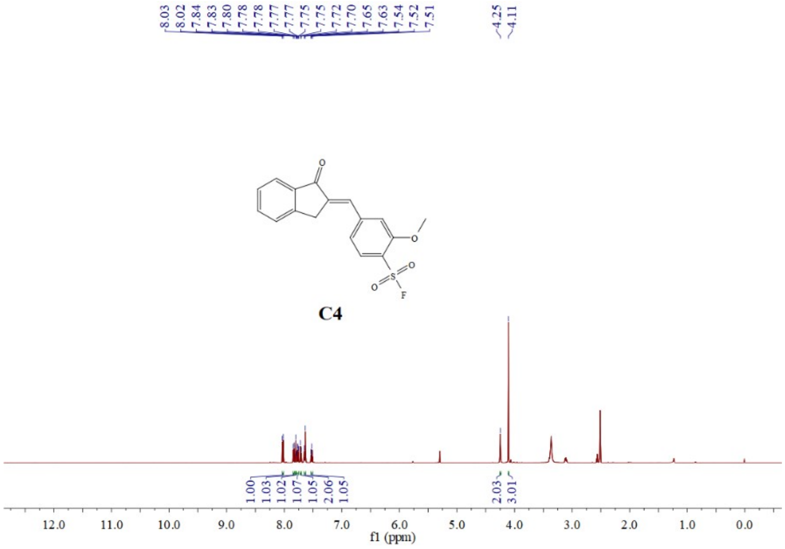
8.03
8.02
7.84
7.83
7.80
7.78
7.78
7.77
7.77
7.75
7.75
7.72
7.70
7.65
7.63
7.54
7.52
7.51
4.25
4.11
O
O
S
O
O
F
C4
1.00
1.03
1.02
1.07
1.05
2.06
1.05
2.03
3.01
12.0 11.0 10.0 9.0 8.0 7.0 6.0 5.0 4.0 3.0 2.0 1.0 0.0
f1 (ppm)

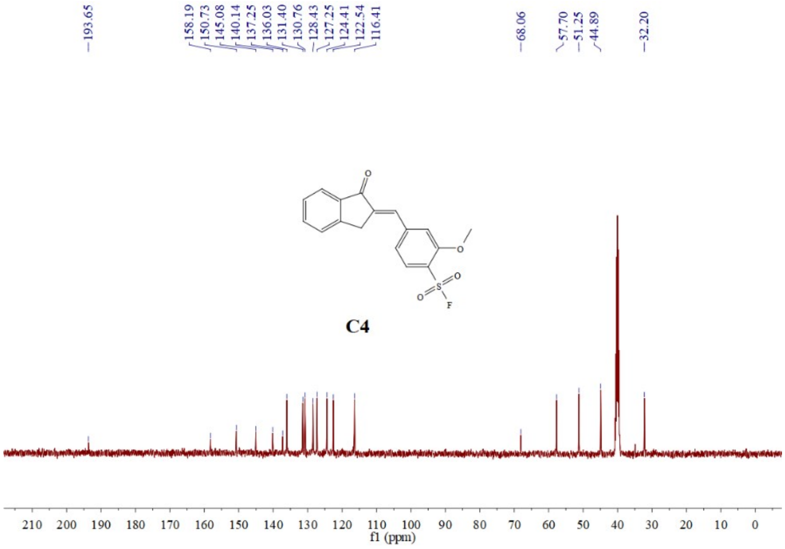
193.65
158.19
150.73
145.08
140.14
137.25
136.03
131.40
130.76
128.43
127.25
124.41
122.54
116.41
68.06
57.70
51.25
44.89
32.20
O
O
S
O
O
F
C4
210 200 190 180 170 160 150 140 130 120 110 100 90 80 70 60 50 40 30 20 10 0
f1 (ppm)

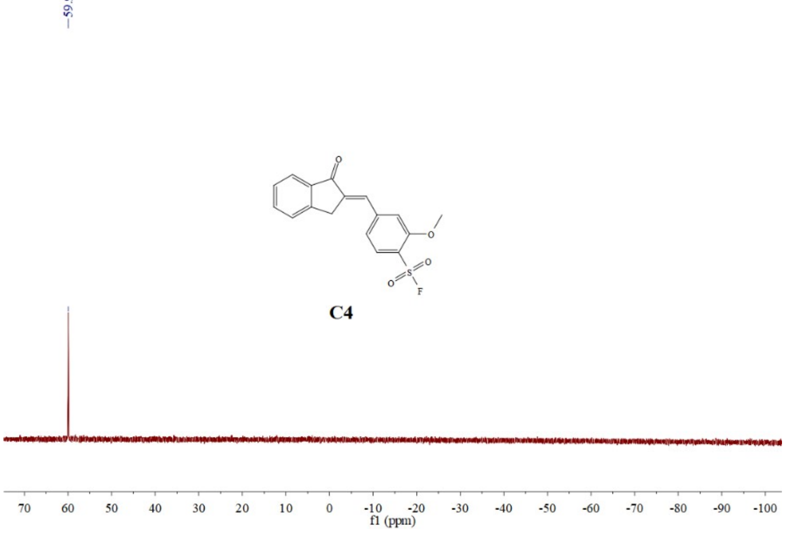
59.94
O
O
S
O
O
F
C4
70 60 50 40 30 20 10 0 -10 -20 -30 -40 -50 -60 -70 -80 -90 -100
f1 (ppm)

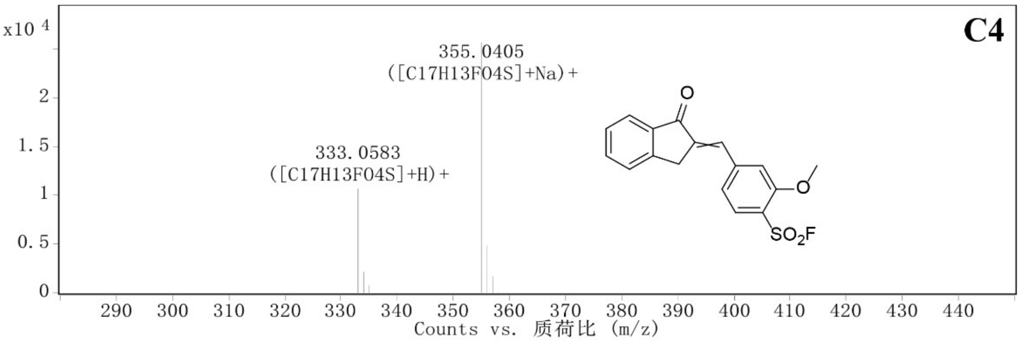

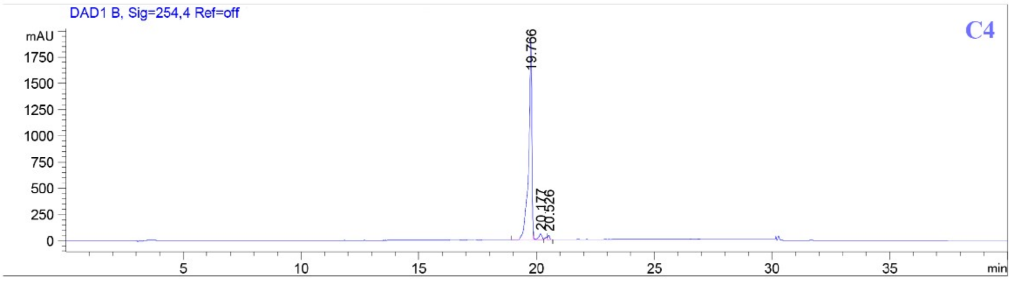

Signal 2: DAD1 B, Sig=254,4 Ref=off

Peak #	Retention time [min]	Type	Peak width [min]	Peak area [mAU*s]	Peak height [mAU]	Peak area %
1	19.766	BV R	0.1316	1.82192e4	1923.03247	95.6119
2	20.177	VV E	0.1545	582.54578	54.60762	3.0571
3	20.526	VB E	0.0914	253.62495	41.41702	1.3310
Total amount :				1.90554e4	2019.05710	

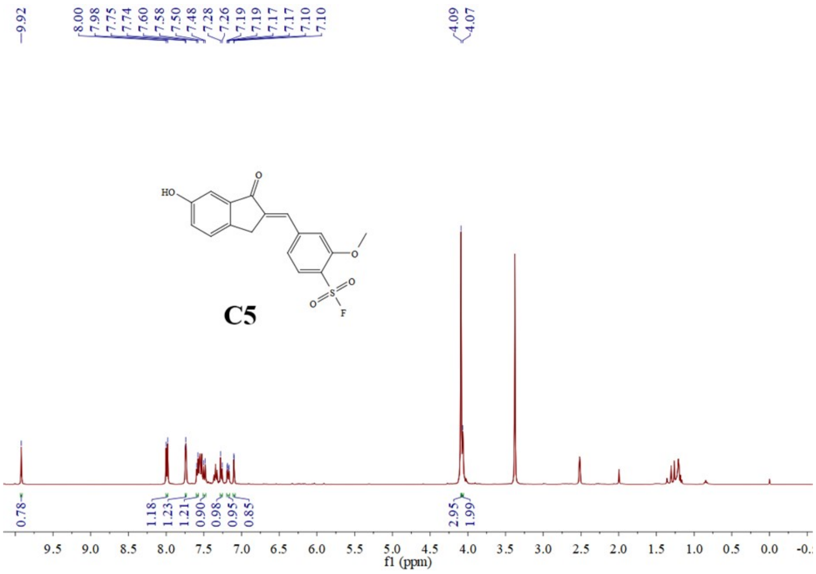

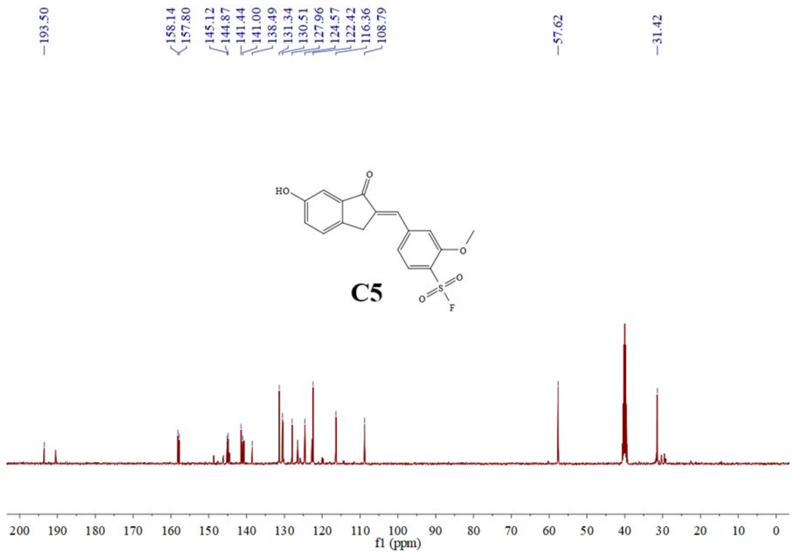
193.50
158.14
157.80
145.12
144.87
141.44
141.00
138.49
131.34
130.51
127.96
124.57
122.42
116.36
108.79
57.62
31.42
HO
O
C5
F
f1 (ppm)

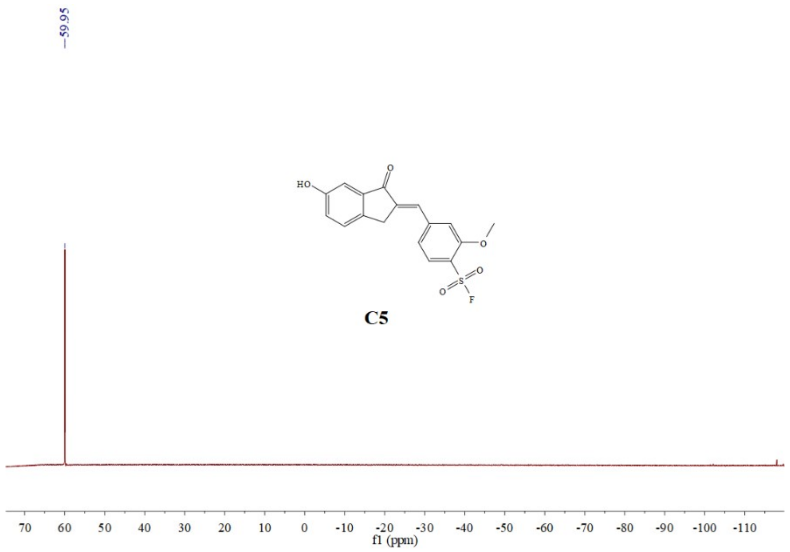
-59.95
HO
O
C5
F
f1 (ppm)

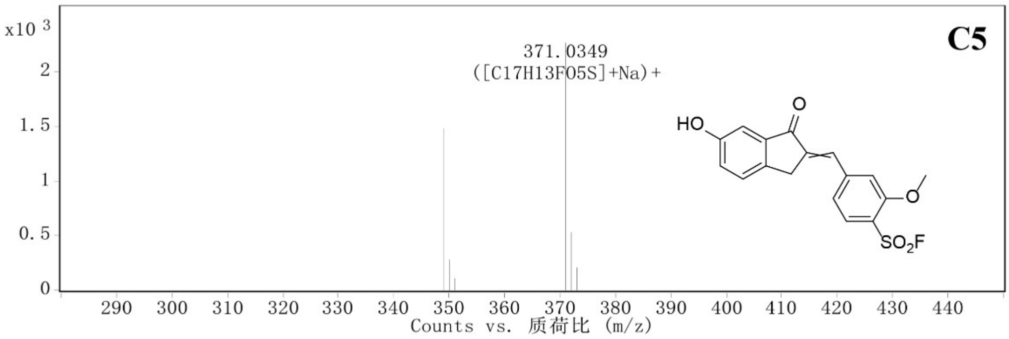
x10 3
371.0349
([C17H13FO5S]+Na)+
C5
HO
O
SO2F
Counts vs. 质荷比 (m/z)

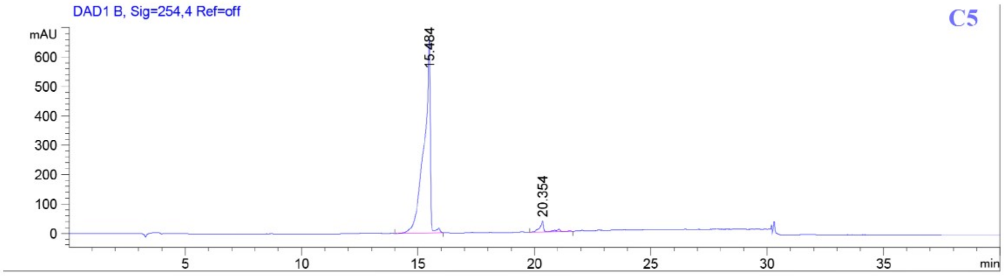

Signal 2: DAD1 B, Sig=254,4 Ref=off

Peak #	Retention time [min]	Type	Peak width [min]	Peak area [mAU*s]	Peak height [mAU]	Peak area %
59	30.275	VB	0.1684	7127.85156	552.82898	10.5808
60	32.555	BV	1.0609	2449.64893	27.19567	3.6363
61	32.678	VV	0.1159	145.06548	15.97883	0.2153
62	32.818	VB	0.1852	58.10279	3.95993	0.0862
63	34.916	BV	0.0648	5.22159	1.11146	7.751e-3

Total amount : 6.73659e4 4611.16629

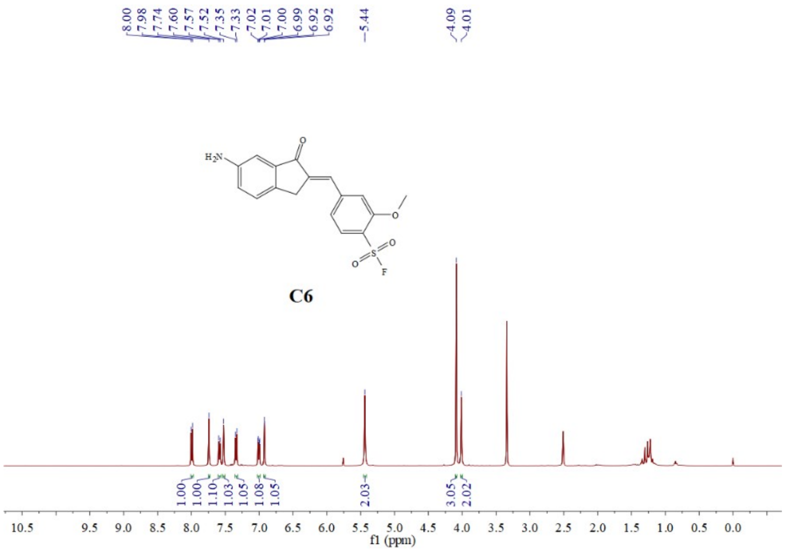

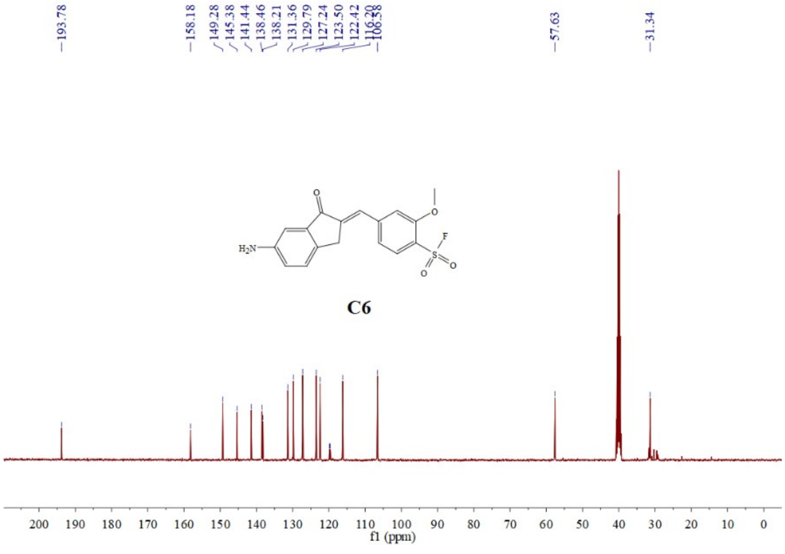

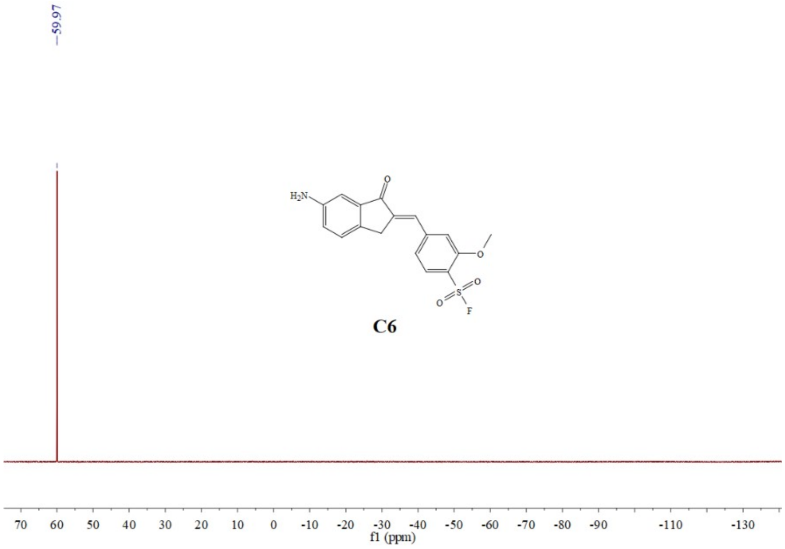

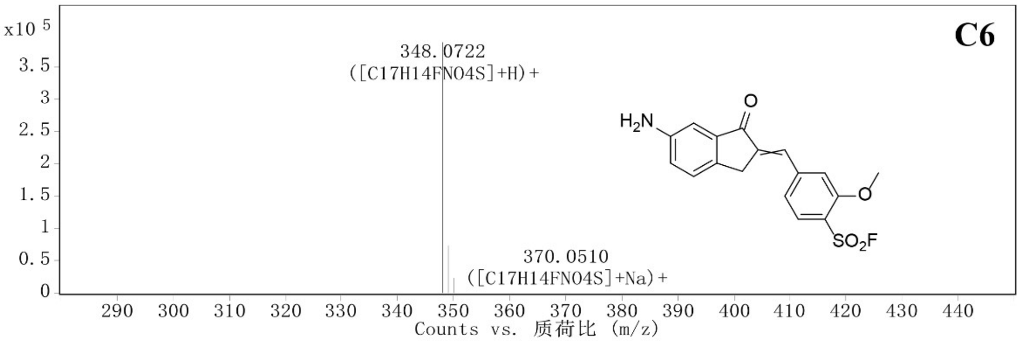

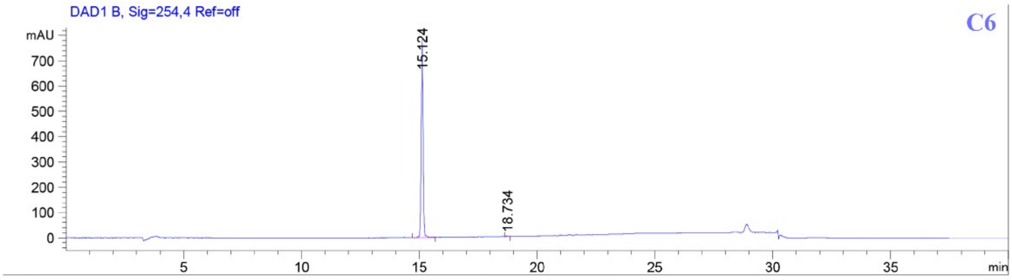

Signal 2: DAD1 B, Sig=254,4 Ref=off

Peak #	Retention time [min]	Type	Peak width [min]	Peak area [mAU*s]	Peak height [mAU]	Peak area %
1	15.124	BV R	0.0978	4886.37354	790.93787	99.6991
2	18.734	VB	0.0911	14.74926	2.42044	0.3009

Total amount : 4901.12280 793.35830

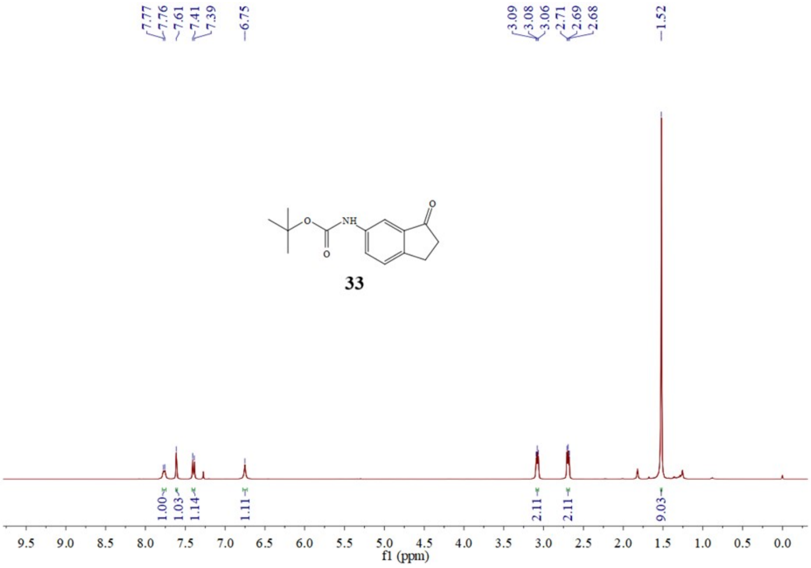
7.77
7.76
7.61
7.41
7.39
6.75
3.09
3.08
3.06
2.71
2.69
2.68
1.52
NH
O
33
1.00
1.03
1.14
1.11
2.11
2.11
9.03
9.5 9.0 8.5 8.0 7.5 7.0 6.5 6.0 5.5 5.0 4.5 4.0 3.5 3.0 2.5 2.0 1.5 1.0 0.5 0.0
f1 (ppm)

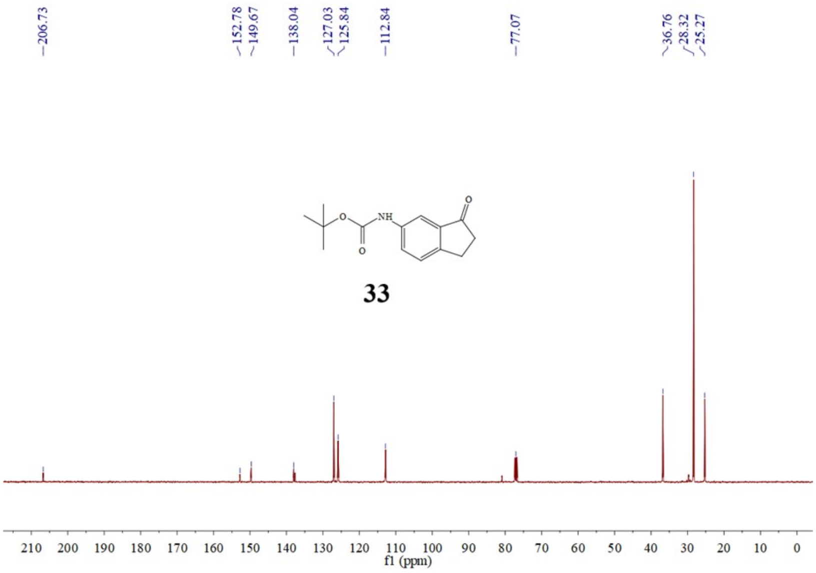
206.73
152.78
149.67
138.04
127.03
125.84
112.84
77.07
36.76
28.32
25.27
NH
O
33
210 200 190 180 170 160 150 140 130 120 110 100 90 80 70 60 50 40 30 20 10 0
f1 (ppm)

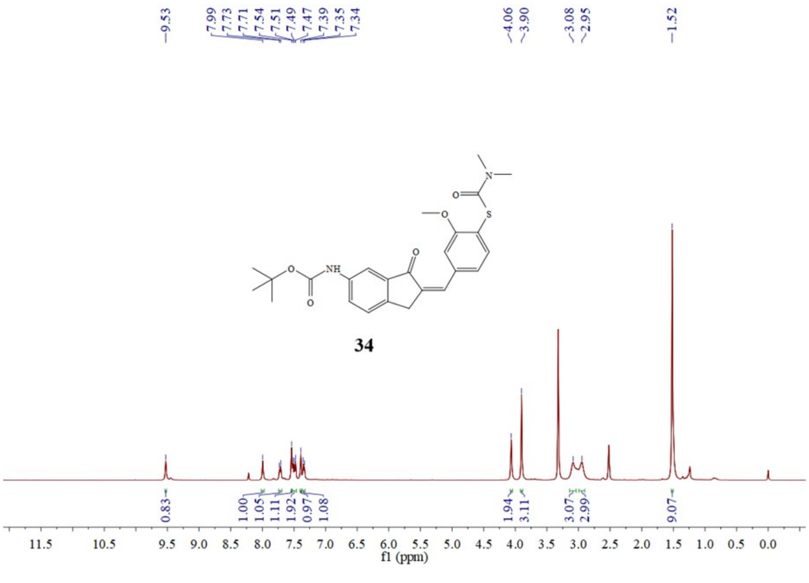
9.53
7.99
7.73
7.71
7.54
7.51
7.49
7.47
7.39
7.35
7.34
4.06
3.90
3.08
2.95
1.52
N
O
S
NH
34
0.83
1.00
1.05
1.11
1.92
0.97
1.08
1.94
3.11
3.07
2.99
9.07
11.5 10.5 9.5 9.0 8.5 8.0 7.5 7.0 6.5 6.0 5.5 5.0 4.5 4.0 3.5 3.0 2.5 2.0 1.5 1.0 0.5 0.0
f1 (ppm)

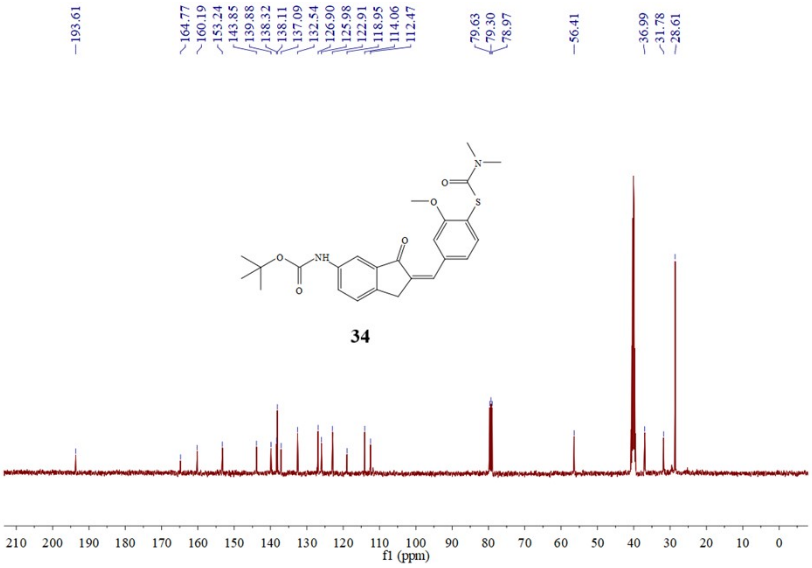
193.61
164.77
160.19
153.24
143.85
139.88
138.32
138.11
137.09
132.54
126.90
125.98
122.91
118.95
114.06
112.47
79.63
79.30
78.97
56.41
36.99
31.78
28.61
34
210 200 190 180 170 160 150 140 130 120 110 100 90 80 70 60 50 40 30 20 10 0
f1 (ppm)

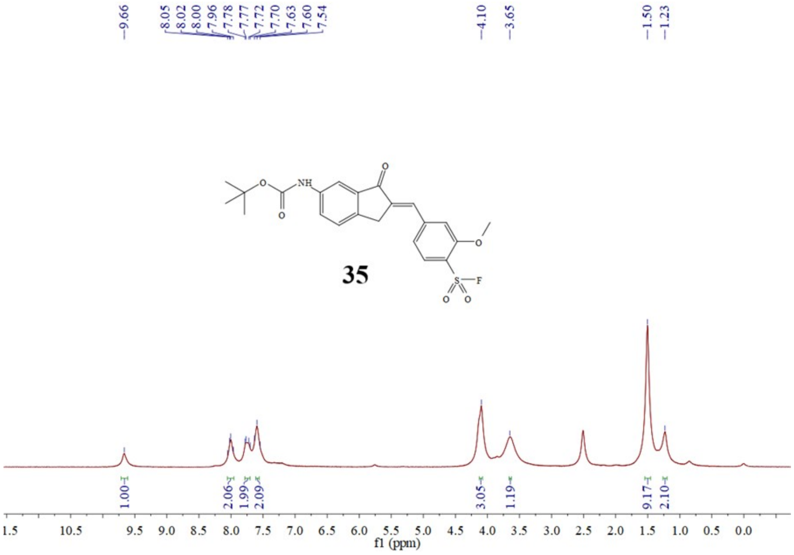
9.66
8.05
8.02
8.00
7.96
7.78
7.77
7.72
7.70
7.63
7.60
7.54
4.10
3.65
1.50
1.23
35
1.00
2.06
1.99
2.09
3.05
1.19
9.17
2.10
1.5 10.5 9.5 9.0 8.5 8.0 7.5 7.0 6.5 6.0 5.5 5.0 4.5 4.0 3.5 3.0 2.5 2.0 1.5 1.0 0.5 0.0
f1 (ppm)

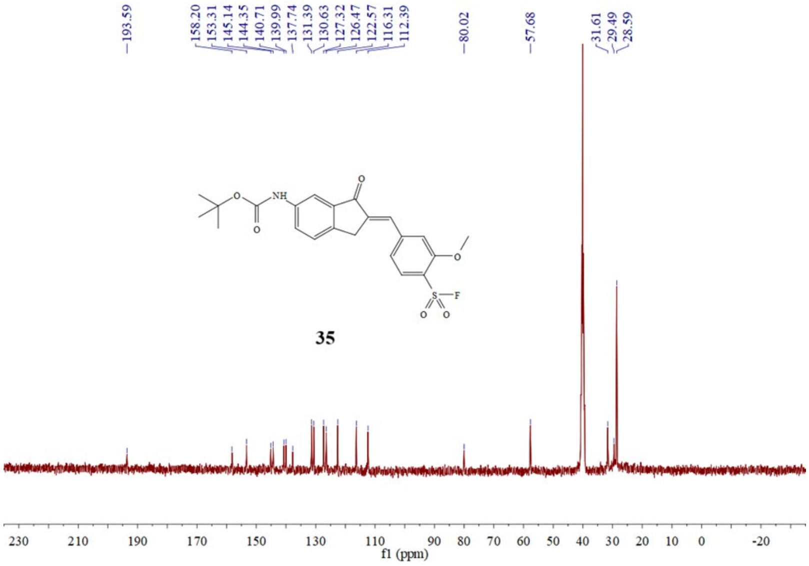
193.59
158.20
153.31
145.14
144.35
140.71
139.99
137.74
131.39
130.63
127.32
126.47
122.57
116.31
112.39
80.02
57.68
31.61
29.49
28.59
35
230 210 190 170 150 130 110 90 80 70 60 50 40 30 20 10 0 -20
f1 (ppm)

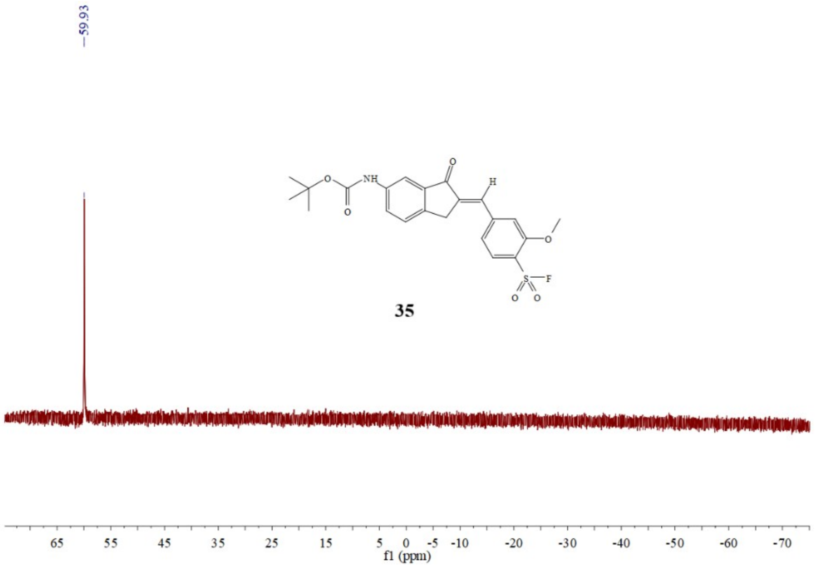

References

1. A. J. Perkowski, C. L. Cruz and D. A. Nicewicz, *Journal of the American Chemical Society*, 2015, **137**, 15684-15687.
2. J. Chen, J. Mao, Y. He, D. Shi, B. Zou and G. Zhang, *Tetrahedron*, 2015, **71**, 9496-9500.
3. Y. q. Yuan, S. Guo and J. Xiang, *Synlett*, 2013, **24**, 443 - 448.
4. C. N. Njiojob, E. A. Owens, L. Narayana, H. Hyun, H. S. Choi and M. Henary, *Journal of Medicinal Chemistry*, 2015, **58**, 2845-2854.
5. M. E. Matter, L. Čamdžić and E. E. Stache, *Angewandte Chemie International Edition*, 2023, **62**, e202308648.
6. L. Matesic, N. A. Wyatt, B. H. Fraser, M. P. Roberts, T. Q. Pham and I. Greguric, *The Journal of Organic Chemistry*, 2013, **78**, 11262-11270.
7. J. K. Park, J. Oh and S. Lee, *Organic Chemistry Frontiers*, 2022, **9**, 3407-3413.
8. Y. Ma, Q. Pan, C. Ou, Y. Cai, X. Ma and C. Liu, *Organic & Biomolecular Chemistry*, 2023, **21**, 7597-7601.
9. X. Wu and B. Gao, *Organic Letters*, 2023, **25**, 8722-8726.
10. T. T. Bui, V. H. Tran and H.-K. Kim, *Advanced Synthesis & Catalysis*, 2022, **364**, 341-347.
